# Supplementary material for: Unraveling the Drifting Larval Fish Community in a Large Spawning Ground in the Middle Pearl River Using DNA Barcoding
Source: Animals (Basel). 2022 Sep 24;12(19):2555. doi: 10.3390/ani12192555 (PMC9559676; doi:10.3390/ani12192555)
Supplement: Supplementary file 1 [file animals-12-02555-s001.zip › Table S1.pdf]

Sample date	Sample ID	Species	Genbank Accession
2018/5/8	SZ1	*Coptodon zillii*	OP050522
2018/5/8	SZ2	*Hyporhamphus intermedius*	OP050552
2018/5/8	SZ3	*Siniperca* sp.	NA
2018/5/8	SZ4	*Hyporhamphus intermedius*	OP050554
2018/5/8	SZ5	*Hyporhamphus intermedius*	OP050555
2018/5/8	SZ6	*Hemiculter leucisculus*	OP050541
2018/5/8	SZ7	*Rhinogobius* sp. 4	NA
2018/5/8	SZ8	*Pseudohemiculter dispar*	OP050700
2018/5/8	SZ9	*Siniperca* sp.	NA
2018/5/8	SZ10	*Siniperca scherzeri*	OP050803
2018/5/8	SZ11	*Zacco platypus*	OP051037
2018/5/8	SZ12	*Siniperca* sp.	NA
2018/5/8	SZ13	*Siniperca* sp.	NA
2018/5/8	SZ14	*Siniperca* sp.	NA
2018/5/8	SZ15	*Opsariichthys bidens*	OP050570
2018/5/8	SZ16	*Zacco platypus*	OP051040
2018/5/8	SZ17	*Pseudohemiculter dispar*	OP050593
2018/5/8	SZ18	*Zacco platypus*	OP051043
2018/5/8	SZ19	*Zacco platypus*	OP051044
2018/5/8	SZ20	*Pseudohemiculter dispar*	OP050599
2018/5/8	SZ21	*Siniperca* sp.	NA
2018/5/8	SZ22	*Siniperca* sp.	NA
2018/5/8	SZ24	*Zacco platypus*	OP051051
2018/5/8	SZ25	*Pseudohemiculter dispar*	OP050606
2018/5/8	SZ26	*Siniperca* sp.	NA
2018/5/8	SZ27	*Siniperca* sp.	NA
2018/5/8	SZ28	*Pseudohemiculter dispar*	OP050616
2018/5/8	SZ29	*Rhinogobius* sp. 3	NA
2018/5/8	SZ30	*Sinibrama macrops*	OP050795
2018/5/8	SZ31	*Siniperca scherzeri*	OP050831
2018/5/8	SZ32	*Siniperca* sp.	NA
2018/5/8	SZ33	*Siniperca scherzeri*	OP050832
2018/5/8	SZ34	*Siniperca* sp.	NA
2018/5/8	SZ35	*Siniperca scherzeri*	OP050837
2018/5/8	SZ36	*Siniperca* sp.	NA
2018/5/8	SZ37	*Siniperca* sp.	NA
2018/5/8	SZ38	*Siniperca* sp.	NA
2018/5/8	SZ39	*Siniperca* sp.	NA
2018/5/8	SZ40	*Siniperca* sp.	NA
2018/5/8	SZ41	*Siniperca* sp.	NA
2018/5/8	SZ42	*Pseudohemiculter dispar*	OP050636
2018/5/8	SZ43	*Siniperca* sp.	NA

2018/5/8	SZ44	*Zacco platypus*	OP051055
2018/5/8	SZ45	*Hemiculter leucisculus*	OP050539
2018/5/8	SZ46	*Pseudolaubuca engraulis*	OP050725
2018/5/8	SZ47	*Pseudohemiculter dispar*	OP050644
2018/5/8	SZ48	*Pseudohemiculter dispar*	OP050645
2018/5/8	SZ49	*Pseudohemiculter dispar*	OP050646
2018/5/8	SZ50	*Rhinogobius* sp. 1	NA
2018/5/8	SZ51	*Pseudohemiculter dispar*	OP050647
2018/5/8	SZ52	*Rhinogobius* sp. 4	NA
2018/5/8	SZ53	*Pseudohemiculter dispar*	OP050653
2018/5/8	SZ54	*Pseudohemiculter dispar*	OP050655
2018/5/8	SZ55	*Pseudohemiculter dispar*	OP050657
2018/5/8	SZ56	*Pseudohemiculter dispar*	OP050660
2018/5/8	SZ57	*Pseudohemiculter dispar*	OP050664
2018/5/8	SZ58	*Pseudohemiculter dispar*	OP050669
2018/5/8	SZ59	*Pseudohemiculter dispar*	OP050673
2018/5/8	SZ60	*Pseudohemiculter dispar*	OP050677
2018/5/8	SZ61	*Siniperca* sp.	NA
2018/5/8	SZ62	*Pseudohemiculter dispar*	OP050680
2018/5/8	SZ63	*Pseudohemiculter dispar*	OP050681
2018/5/8	SZ64	*Hemiculter leucisculus*	OP050542
2018/5/8	SZ65	*Pseudohemiculter dispar*	OP050682
2018/5/8	SZ66	*Pseudohemiculter dispar*	OP050683
2018/5/8	SZ67	*Pseudohemiculter dispar*	OP050686
2018/5/8	SZ68	*Pseudohemiculter dispar*	OP050687
2018/5/8	SZ69	*Pseudohemiculter dispar*	OP050688
2018/5/8	SZ70	*Rhinogobius* sp. 1	NA
2018/5/8	SZ71	*Pseudohemiculter dispar*	OP050692
2018/5/8	SZ72	*Hemiculter leucisculus*	OP050543
2018/5/14	SZ102	*Pseudohemiculter dispar*	OP050574
2018/5/14	SZ73	*Pseudohemiculter dispar*	OP050697
2018/5/14	SZ74	*Siniperca scherzeri*	OP050851
2018/5/14	SZ75	*Coilia grayii*	OP050515
2018/5/14	SZ76	*Pseudolaubuca engraulis*	OP050729
2018/5/14	SZ77	*Pseudohemiculter dispar*	OP050698
2018/5/14	SZ78	*Siniperca scherzeri*	OP050852
2018/5/14	SZ79	*Pseudohemiculter dispar*	OP050699
2018/5/14	SZ80	*Pseudohemiculter dispar*	OP050701
2018/5/14	SZ81	*Hemiculterella sauvagei*	OP050546
2018/5/14	SZ82	*Zacco platypus*	OP051056
2018/5/14	SZ83	*Pseudohemiculter dispar*	OP050702
2018/5/14	SZ84	*Pseudohemiculter dispar*	OP050703
2018/5/14	SZ85	*Pseudohemiculter dispar*	OP050704
2018/5/14	SZ86	*Pseudohemiculter dispar*	OP050705

2018/5/14	SZ87	*Pseudohemiculter dispar*	OP050706
2018/5/14	SZ88	*Pseudohemiculter dispar*	OP050707
2018/5/14	SZ89	*Pseudohemiculter dispar*	OP050708
2018/5/14	SZ90	*Zacco platypus*	OP051057
2018/5/14	SZ91	*Pseudohemiculter dispar*	OP050709
2018/5/14	SZ92	*Rhinogobius* sp. 1	NA
2018/5/14	SZ93	*Pseudohemiculter dispar*	OP050710
2018/5/14	SZ94	*Pseudohemiculter dispar*	OP050711
2018/5/14	SZ95	*Pseudohemiculter dispar*	OP050712
2018/5/14	SZ96	*Pseudohemiculter dispar*	OP050713
2018/5/14	SZ97	*Pseudohemiculter dispar*	OP050714
2018/5/14	SZ98	*Pseudohemiculter dispar*	OP050715
2018/5/14	SZ99	*Pseudohemiculter dispar*	OP050716
2018/5/14	SZ100	*Pseudohemiculter dispar*	OP050572
2018/5/14	SZ101	*Pseudohemiculter dispar*	OP050573
2018/5/18	SZ103	*Coilia grayii*	OP050516
2018/5/18	SZ104	*Rhinogobius* sp. 1	NA
2018/5/18	SZ105	*Rhinogobius* sp. 1	NA
2018/5/18	SZ106	*Mugilogobius myxodermus*	OP050558
2018/5/18	SZ107	*Rhinogobius* sp. 1	NA
2018/5/18	SZ108	*Coilia grayii*	OP050517
2018/5/18	SZ109	*Siniperca scherzeri*	OP050804
2018/5/18	SZ110	*Chanodichthys recurviceps*	OP050463
2018/5/18	SZ111	*Mugilogobius myxodermus*	OP050559
2018/5/18	SZ113	*Chanodichthys recurviceps*	OP050464
2018/5/18	SZ114	*Coilia grayii*	OP050518
2018/5/18	SZ115	*Rhinogobius* sp. 2	NA
2018/5/18	SZ116	*Mugilogobius myxodermus*	OP050560
2018/5/18	SZ117	*Hyporhamphus intermedius*	OP050550
2018/5/18	SZ118	*Zacco platypus*	OP051038
2018/5/18	SZ119	*Chanodichthys recurviceps*	OP050465
2018/5/18	SZ120	*Pseudohemiculter dispar*	OP050575
2018/5/18	SZ121	*Siniperca scherzeri*	OP050805
2018/5/18	SZ122	*Chanodichthys recurviceps*	OP050466
2018/5/18	SZ123	*Megalobrama terminalis*	OP050557
2018/5/18	SZ124	*Sicyopterus* sp.	NA
2018/5/18	SZ125	*Pseudohemiculter dispar*	OP050576
2018/5/18	SZ126	*Siniperca* sp.	NA
2018/5/18	SZ127	*Coilia grayii*	OP050519
2018/5/18	SZ128	*Chanodichthys recurviceps*	OP050467
2018/5/18	SZ129	*Rhinogobius* sp. 3	NA
2018/5/18	SZ130	*Squalidus argentatus*	OP050853
2018/5/18	SZ131	*Squalidus argentatus*	OP050854
2018/5/18	SZ132	*Squalidus argentatus*	OP050855

2018/5/18	SZ133	*Chanodichthys recurviceps*	OP050468
2018/5/18	SZ134	*Squalidus argentatus*	OP050856
2018/5/18	SZ135	*Rhinogobius* sp. 1	NA
2018/5/18	SZ136	*Rhinogobius* sp. 4	NA
2018/5/18	SZ137	*Pseudohemiculter dispar*	OP050577
2018/5/18	SZ138	*Siniperca scherzeri*	OP050806
2018/5/18	SZ139	*Chanodichthys recurviceps*	OP050469
2018/5/18	SZ140	*Rhinogobius* sp. 1	NA
2018/5/23	SZ141	*Oreochromis* sp. 1	NA
2018/5/23	SZ142	*Oreochromis* sp. 1	NA
2018/5/23	SZ143	*Siniperca scherzeri*	OP050807
2018/5/23	SZ144	*Siniperca scherzeri*	OP050808
2018/5/23	SZ145	*Siniperca scherzeri*	OP050809
2018/5/23	SZ146	*Siniperca scherzeri*	OP050810
2018/5/23	SZ147	*Hyporhamphus intermedius*	OP050551
2018/5/23	SZ148	*Pseudohemiculter dispar*	OP050578
2018/5/23	SZ149	*Siniperca scherzeri*	OP050811
2018/5/23	SZ150	*Siniperca scherzeri*	OP050812
2018/5/23	SZ151	*Zacco platypus*	OP051039
2018/5/23	SZ153	*Hemiculter leucisculus*	OP050526
2018/5/23	SZ154	*Pseudohemiculter dispar*	OP050580
2018/5/23	SZ155	*Pseudohemiculter dispar*	OP050581
2018/5/23	SZ156	*Pseudohemiculter dispar*	OP050582
2018/5/23	SZ157	*Pseudohemiculter dispar*	OP050583
2018/5/23	SZ158	*Hemiculter leucisculus*	OP050527
2018/5/23	SZ159	*Pseudohemiculter dispar*	OP050584
2018/5/23	SZ160	*Zacco platypus*	OP051041
2018/5/23	SZ161	*Pseudohemiculter dispar*	OP050585
2018/5/23	SZ162	*Pseudohemiculter dispar*	OP050586
2018/5/23	SZ163	*Pseudohemiculter dispar*	OP050587
2018/5/23	SZ164	*Hemiculter leucisculus*	OP050528
2018/5/23	SZ165	*Pseudohemiculter dispar*	OP050588
2018/5/23	SZ166	*Pseudohemiculter dispar*	OP050589
2018/5/23	SZ167	*Pseudohemiculter dispar*	OP050590
2018/5/23	SZ168	*Pseudohemiculter dispar*	OP050591
2018/5/23	SZ169	*Pseudohemiculter dispar*	OP050592
2018/5/23	SZ170	*Pseudohemiculter dispar*	OP050594
2018/5/24	SZ152	*Pseudohemiculter dispar*	OP050579
2018/5/29	SZ171	*Siniperca scherzeri*	OP050813
2018/5/29	SZ172	*Pseudolaubuca sinensis*	OP050731
2018/5/29	SZ173	*Zacco platypus*	OP051042
2018/5/29	SZ174	*Siniperca scherzeri*	OP050814
2018/5/29	SZ175	*Hemiculter leucisculus*	OP050529
2018/5/29	SZ176	*Pseudohemiculter dispar*	OP050595

2018/5/29	SZ177	*Rhinogobius* sp. 6	NA
2018/5/29	SZ178	*Pseudohemiculter dispar*	OP050596
2018/5/29	SZ179	*Rhinogobius* sp. 2	NA
2018/5/29	SZ180	*Pseudohemiculter dispar*	OP050597
2018/6/1	SZ181	*Neosalanx* sp.	NA
2018/6/1	SZ182	*Coilia grayii*	OP050520
2018/6/1	SZ183	*Oreochromis* sp. 1	NA
2018/6/1	SZ185	Acheilognathinae	NA
2018/6/1	SZ186	*Siniperca* sp.	NA
2018/6/1	SZ187	*Coilia grayii*	OP050521
2018/6/1	SZ188	*Sinibrama macrops*	OP050787
2018/6/1	SZ189	*Rhinogobius* sp. 5	NA
2018/6/1	SZ190	*Sinibrama macrops*	OP050788
2018/6/1	SZ191	*Zacco platypus*	OP051045
2018/6/1	SZ193	*Chanodichthys recurviceps*	OP050470
2018/6/1	SZ194	*Zacco platypus*	OP051046
2018/6/1	SZ195	*Siniperca scherzeri*	OP050815
2018/6/1	SZ196	*Rhinogobius* sp. 4	NA
2018/6/1	SZ197	*Oreochromis* sp. 1	NA
2018/6/1	SZ198	*Sinibotia robusta*	OP050742
2018/6/1	SZ200	*Zacco platypus*	OP051047
2018/6/1	SZ201	*Pseudohemiculter dispar*	OP050600
2018/6/1	SZ202	*Sinibrama macrops*	OP050789
2018/6/1	SZ203	*Siniperca* sp.	NA
2018/6/1	SZ204	*Sinibrama macrops*	OP050790
2018/6/1	SZ205	*Rhinogobius* sp. 3	NA
2018/6/1	SZ206	*Mugilogobius myxodermus*	OP050561
2018/6/1	SZ207	*Zacco platypus*	OP051048
2018/6/1	SZ208	*Mugilogobius myxodermus*	OP050562
2018/6/1	SZ209	*Rhinogobius* sp. 1	NA
2018/6/1	SZ210	*Sicyopterus* sp.	NA
2018/6/1	SZ211	*Pseudohemiculter dispar*	OP050602
2018/6/1	SZ212	*Pseudohemiculter dispar*	OP050603
2018/6/1	SZ215	*Sinibrama macrops*	OP050791
2018/6/1	SZ218	*Squalidus argentatus*	OP050868
2018/6/1	SZ219	*Zacco platypus*	OP051049
2018/6/1	SZ220	*Rhinogobius* sp. 4	NA
2018/6/1	SZ221	*Siniperca scherzeri*	OP050816
2018/6/1	SZ222	*Zacco platypus*	OP051050
2018/6/1	SZ223	*Hemiculter leucisculus*	OP050531
2018/6/1	SZ224	*Rhinogobius* sp. 1	NA
2018/6/1	SZ225	*Siniperca* sp.	NA
2018/6/1	SZ226	*Siniperca* sp.	NA
2018/6/1	SZ227	*Rhinogobius* sp. 4	NA

2018/6/1	SZ229	*Pseudohemiculter dispar*	OP050604
2018/6/1	SZ230	*Rhinogobius* sp. 1	NA
2018/6/1	SZ231	*Chanodichthys recurviceps*	OP050471
2018/6/1	SZ232	*Siniperca scherzeri*	OP050817
2018/6/1	SZ233	*Siniperca* sp.	NA
2018/6/1	SZ234	*Sinibrama macrops*	OP050792
2018/6/1	SZ235	*Hyporhamphus intermedius*	OP050553
2018/6/1	SZ236	*Sinibotia robusta*	OP050743
2018/6/1	SZ237	*Opsariichthys bidens*	OP050571
2018/6/1	SZ238	*Coptodon zillii*	OP050523
2018/6/1	SZ239	*Sinibrama macrops*	OP050793
2018/6/1	SZ240	*Sinibrama macrops*	OP050794
2018/6/1	SZ241	*Pseudolaubuca sinensis*	OP050732
2018/6/1	SZ242	*Hemiculter leucisculus*	OP050532
2018/6/1	SZ243	*Pseudohemiculter dispar*	OP050605
2018/6/1	SZ244	*Zacco platypus*	OP051052
2018/6/1	SZ251	*Siniperca scherzeri*	OP050818
2018/6/5	SZ252	*Siniperca scherzeri*	OP050819
2018/6/5	SZ254	*Siniperca* sp.	NA
2018/6/5	SZ255	*Siniperca scherzeri*	OP050820
2018/6/5	SZ256	*Siniperca* sp.	NA
2018/6/5	SZ258	*Pseudohemiculter dispar*	OP050607
2018/6/5	SZ259	*Pseudolaubuca engraulis*	OP050718
2018/6/5	SZ260	*Pseudorasbora parva*	OP050740
2018/6/5	SZ262	*Pseudolaubuca engraulis*	OP050719
2018/6/5	SZ263	*Pseudohemiculter dispar*	OP050608
2018/6/5	SZ264	*Pseudohemiculter dispar*	OP050609
2018/6/5	SZ265	*Pseudohemiculter dispar*	OP050610
2018/6/5	SZ266	*Siniperca* sp.	NA
2018/6/5	SZ267	*Pseudolaubuca engraulis*	OP050720
2018/6/5	SZ268	*Pseudohemiculter dispar*	OP050611
2018/6/5	SZ269	*Hemiculter leucisculus*	OP050533
2018/6/10	SZ271	*Squalidus argentatus*	OP050857
2018/6/10	SZ275	*Siniperca scherzeri*	OP050828
2018/6/10	SZ276	*Siniperca* sp.	NA
2018/6/10	SZ277	*Chanodichthys recurviceps*	OP050472
2018/6/10	SZ278	*Pseudohemiculter dispar*	OP050613
2018/6/10	SZ279	*Pseudohemiculter dispar*	OP050614
2018/6/10	SZ280	*Mylopharyngodon piceus*	OP050566
2018/6/10	SZ281	*Squalidus argentatus*	OP050858
2018/6/10	SZ282	*Rhinogobius* sp. 3	NA
2018/6/10	SZ284	*Rhinogobius* sp. 5	NA
2018/6/10	SZ285	*Chanodichthys* sp.	NA
2018/6/10	SZ286	*Squaliobarbus curriculus*	OP050880

2018/6/10	SZ288	*Hemiculter leucisculus*	OP050534
2018/6/10	SZ289	*Chanodichthys recurviceps*	OP050476
2018/6/10	SZ290	*Chanodichthys recurviceps*	OP050477
2018/6/10	SZ291	*Rhinogobius* sp. 4	NA
2018/6/10	SZ292	*Pseudohemiculter dispar*	OP050619
2018/6/10	SZ293	*Zacco platypus*	OP051053
2018/6/10	SZ294	*Hemiculter leucisculus*	OP050535
2018/6/10	SZ295	*Hemiculter leucisculus*	OP050536
2018/6/10	SZ296	*Oreochromis* sp. 3	NA
2018/6/10	SZ297	*Hemiculter leucisculus*	OP050537
2018/6/15	SZ298	*Pseudohemiculter dispar*	OP050621
2018/6/15	SZ299	*Pseudohemiculter dispar*	OP050622
2018/6/15	SZ313	*Hemiculterella sauvagei*	OP050545
2018/6/15	SZ314	*Rhinogobius* sp. 3	NA
2018/6/15	SZ315	*Rhinogobius cliffordpopei*	OP050741
2018/6/15	SZ316	*Rhinogobius* sp. 3	NA
2018/6/15	SZ318	*Rhinogobius* sp. 3	NA
2018/6/15	SZ319	*Squaliobarbus curriculus*	OP050894
2018/6/15	SZ321	*Rhinogobius* sp. 4	NA
2018/6/15	SZ322	*Rhinogobius* sp. 4	NA
2018/6/15	SZ326	*Hemiculter leucisculus*	OP050538
2018/6/15	SZ327	*Pseudohemiculter dispar*	OP050623
2018/6/15	SZ332	*Siniperca scherzeri*	OP050833
2018/6/15	SZ333	*Siniperca scherzeri*	OP050834
2018/6/15	SZ334	*Siniperca* sp.	NA
2018/6/15	SZ335	*Rhinogobius* sp. 4	NA
2018/6/15	SZ336	*Siniperca scherzeri*	OP050835
2018/6/15	SZ337	*Siniperca scherzeri*	OP050836
2018/6/15	SZ338	*Squaliobarbus curriculus*	OP050895
2018/6/15	SZ339	*Squaliobarbus curriculus*	OP050896
2018/6/15	SZ340	*Squalidus argentatus*	OP050859
2018/6/15	SZ341	*Squalidus argentatus*	OP050860
2018/6/15	SZ342	*Pseudohemiculter dispar*	OP050624
2018/6/20	SZ343	*Pseudolaubuca engraulis*	OP050721
2018/6/20	SZ344	*Pseudohemiculter dispar*	OP050625
2018/6/20	SZ345	*Squaliobarbus curriculus*	OP050897
2018/6/20	SZ346	*Siniperca* sp.	NA
2018/6/20	SZ347	*Toxabramis houdemeri*	OP051036
2018/6/20	SZ348	*Pseudolaubuca engraulis*	OP050722
2018/6/20	SZ350	*Pseudohemiculter dispar*	OP050626
2018/6/20	SZ351	*Pseudohemiculter dispar*	OP050627
2018/6/20	SZ352	*Siniperca* sp.	NA
2018/6/20	SZ357	*Pseudolaubuca engraulis*	OP050723
2018/6/20	SZ360	*Pseudolaubuca sinensis*	OP050734

2018/6/20	SZ362	*Rhinogobius* sp. 1	NA
2018/6/20	SZ364	*Sinibrama macrops*	OP050796
2018/6/20	SZ366	*Pseudohemiculter dispar*	OP050628
2018/6/20	SZ367	*Schistura* sp.	NA
2018/6/20	SZ368	*Pseudohemiculter dispar*	OP050629
2018/6/20	SZ371	*Sinibrama macrops*	OP050797
2018/6/20	SZ372	*Squaliobarbus curriculus*	OP050898
2018/6/20	SZ373	*Sinibrama macrops*	OP050798
2018/6/20	SZ374	*Siniperca scherzeri*	OP050838
2018/6/20	SZ375	*Squaliobarbus curriculus*	OP050899
2018/6/20	SZ376	*Squaliobarbus curriculus*	OP050900
2018/6/20	SZ377	*Squaliobarbus curriculus*	OP050901
2018/6/20	SZ378	*Sinibrama melrosei*	OP050802
2018/6/20	SZ379	*Squaliobarbus curriculus*	OP050902
2018/6/20	SZ380	*Squaliobarbus curriculus*	OP050903
2018/6/20	SZ381	*Squaliobarbus curriculus*	OP050904
2018/6/20	SZ382	*Squaliobarbus curriculus*	OP050905
2018/6/20	SZ383	*Sinibotia robusta*	OP050749
2018/6/20	SZ384	*Siniperca* sp.	NA
2018/6/20	SZ385	*Squaliobarbus curriculus*	OP050906
2018/6/20	SZ386	*Siniperca* sp.	NA
2018/6/20	SZ387	*Squaliobarbus curriculus*	OP050907
2018/6/20	SZ388	*Squaliobarbus curriculus*	OP050908
2018/6/20	SZ389	*Squaliobarbus curriculus*	OP050909
2018/6/20	SZ391	*Squaliobarbus curriculus*	OP050910
2018/6/20	SZ392	*Squaliobarbus curriculus*	OP050911
2018/6/20	SZ393	*Squaliobarbus curriculus*	OP050912
2018/6/20	SZ394	*Squaliobarbus curriculus*	OP050913
2018/6/20	SZ395	*Pseudohemiculter dispar*	OP050630
2018/6/20	SZ396	*Squaliobarbus curriculus*	OP050914
2018/6/20	SZ397	*Squaliobarbus curriculus*	OP050915
2018/6/20	SZ398	*Squaliobarbus curriculus*	OP050916
2018/6/20	SZ399	*Squaliobarbus curriculus*	OP050917
2018/6/20	SZ400	*Squaliobarbus curriculus*	OP050918
2018/6/20	SZ401	*Squaliobarbus curriculus*	OP050919
2018/6/20	SZ402	*Pseudohemiculter dispar*	OP050631
2018/6/20	SZ403	*Rhinogobius* sp. 4	NA
2018/6/20	SZ404	*Squaliobarbus curriculus*	OP050920
2018/6/20	SZ405	*Squaliobarbus curriculus*	OP050921
2018/6/20	SZ406	*Squaliobarbus curriculus*	OP050922
2018/6/20	SZ407	*Squaliobarbus curriculus*	OP050923
2018/6/20	SZ408	*Pseudohemiculter dispar*	OP050632
2018/6/20	SZ409	*Squaliobarbus curriculus*	OP050924
2018/6/20	SZ410	*Squaliobarbus curriculus*	OP050925

2018/6/20	SZ411	*Pseudohemiculter dispar*	OP050633
2018/6/20	SZ412	*Squaliobarbus curriculus*	OP050926
2018/6/20	SZ413	*Hemiculterella wui*	OP050547
2018/6/25	SZ414	*Siniperca scherzeri*	OP050839
2018/6/25	SZ415	*Squaliobarbus curriculus*	OP050927
2018/6/25	SZ416	*Pseudohemiculter dispar*	OP050634
2018/6/25	SZ417	*Neosalanx* sp.	NA
2018/6/25	SZ418	*Pseudohemiculter dispar*	OP050635
2018/6/25	SZ419	*Pseudolaubuca engraulis*	OP050724
2018/6/25	SZ420	*Chanodichthys recurviceps*	OP050478
2018/6/30	SZ422	*Pseudohemiculter dispar*	OP050637
2018/6/30	SZ423	*Pseudohemiculter dispar*	OP050638
2018/6/30	SZ424	*Squaliobarbus curriculus*	OP050928
2018/6/30	SZ425	*Squaliobarbus curriculus*	OP050929
2018/6/30	SZ426	*Zacco platypus*	OP051054
2018/6/30	SZ427	*Pseudohemiculter dispar*	OP050639
2018/6/30	SZ428	*Squaliobarbus curriculus*	OP050930
2018/6/30	SZ429	*Pseudohemiculter dispar*	OP050640
2018/6/30	SZ430	*Pseudohemiculter dispar*	OP050641
2018/6/30	SZ431	*Pseudohemiculter dispar*	OP050642
2018/6/30	SZ433	*Squaliobarbus curriculus*	OP050931
2018/6/30	SZ434	*Squaliobarbus curriculus*	OP050932
2018/7/3	SZ435	*Sinibotia robusta*	OP050750
2018/7/3	SZ436	*Squaliobarbus curriculus*	OP050933
2018/7/3	SZ437	*Squaliobarbus curriculus*	OP050934
2018/7/3	SZ438	*Sinibotia robusta*	OP050751
2018/7/3	SZ439	*Squaliobarbus curriculus*	OP050935
2018/7/3	SZ440	*Cirrhinus mrigala*	OP050506
2018/7/3	SZ441	*Xenocypris* sp.	NA
2018/7/3	SZ442	*Squaliobarbus curriculus*	OP050936
2018/7/3	SZ443	*Sinibotia robusta*	OP050752
2018/7/3	SZ444	*Cirrhinus mrigala*	OP050507
2018/7/3	SZ445	*Squaliobarbus curriculus*	OP050937
2018/7/3	SZ446	*Squaliobarbus curriculus*	OP050938
2018/7/3	SZ447	*Squaliobarbus curriculus*	OP050939
2018/7/3	SZ448	*Squaliobarbus curriculus*	OP050940
2018/7/3	SZ449	*Cirrhinus mrigala*	OP050508
2018/7/3	SZ450	*Squaliobarbus curriculus*	OP050941
2018/7/3	SZ451	*Squaliobarbus curriculus*	OP050942
2018/7/3	SZ452	*Squaliobarbus curriculus*	OP050943
2018/7/3	SZ453	*Cirrhinus mrigala*	OP050509
2018/7/3	SZ454	*Squaliobarbus curriculus*	OP050944
2018/7/3	SZ455	*Squaliobarbus curriculus*	OP050945
2018/7/3	SZ456	*Squaliobarbus curriculus*	OP050946

2018/7/3	SZ457	*Squaliobarbus curriculus*	OP050947
2018/7/3	SZ458	*Squaliobarbus curriculus*	OP050948
2018/7/3	SZ459	*Squaliobarbus curriculus*	OP050949
2018/7/3	SZ460	*Cirrhinus mrigala*	OP050510
2018/7/3	SZ461	*Cirrhinus mrigala*	OP050511
2018/7/3	SZ462	*Squaliobarbus curriculus*	OP050950
2018/7/3	SZ463	*Cirrhinus mrigala*	OP050512
2018/7/3	SZ464	*Squaliobarbus curriculus*	OP050951
2018/7/3	SZ465	*Squaliobarbus curriculus*	OP050952
2018/7/3	SZ466	*Hypophthalmichthys molitrix*	OP050548
2018/7/3	SZ467	*Pseudohemiculter dispar*	OP050643
2018/7/3	SZ468	*Cirrhinus mrigala*	OP050513
2018/7/3	SZ469	*Squaliobarbus curriculus*	OP050953
2018/7/3	SZ470	*Siniperca* sp.	NA
2018/7/3	SZ471	*Sinibotia robusta*	OP050753
2018/7/3	SZ472	*Rhinogobius* sp. 1	NA
2018/7/3	SZ473	*Hypostomus* sp.	NA
2018/7/3	SZ474	*Squalidus argentatus*	OP050861
2018/7/3	SZ475	*Garra orientalis*	OP050525
2018/7/3	SZ476	*Mylopharyngodon piceus*	OP050567
2018/7/3	SZ477	*Squaliobarbus curriculus*	OP050954
2018/7/3	SZ478	*Squaliobarbus curriculus*	OP050955
2018/7/3	SZ479	*Pseudolaubuca sinensis*	OP050735
2018/7/3	SZ480	*Mylopharyngodon piceus*	OP050568
2018/7/3	SZ481	*Squaliobarbus curriculus*	OP050956
2018/7/3	SZ482	*Squaliobarbus curriculus*	OP050957
2018/7/3	SZ483	*Squaliobarbus curriculus*	OP050958
2018/7/3	SZ484	*Squaliobarbus curriculus*	OP050959
2018/7/3	SZ485	*Squaliobarbus curriculus*	OP050960
2018/7/3	SZ486	*Squaliobarbus curriculus*	OP050961
2018/7/3	SZ487	*Squaliobarbus curriculus*	OP050962
2018/7/3	SZ488	*Squaliobarbus curriculus*	OP050963
2018/7/3	SZ489	*Squaliobarbus curriculus*	OP050964
2018/7/3	SZ490	*Squaliobarbus curriculus*	OP050965
2018/7/3	SZ491	*Squaliobarbus curriculus*	OP050966
2018/7/3	SZ492	*Hypophthalmichthys molitrix*	OP050549
2018/7/3	SZ493	*Squaliobarbus curriculus*	OP050967
2018/7/3	SZ494	*Chanodichthys recurviceps*	OP050479
2018/7/3	SZ495	*Squaliobarbus curriculus*	OP050968
2018/7/3	SZ496	*Coptodon zillii*	OP050524
2018/7/3	SZ497	*Rhinogobius* sp. 1	NA
2018/7/3	SZ498	*Sinibotia robusta*	OP050754
2018/7/3	SZ499	*Sinibotia robusta*	OP050755
2018/7/3	SZ500	*Cirrhinus mrigala*	OP050514

2018/7/3	SZ501	Acheilognathinae	NA
2018/7/3	SZ502	*Sinibotia robusta*	OP050756
2018/7/3	SZ503	*Oreochromis* sp. 1	NA
2018/7/3	SZ504	*Squaliobarbus curriculus*	OP050969
2018/7/3	SZ505	*Squaliobarbus curriculus*	OP050970
2018/7/3	SZ506	*Squaliobarbus curriculus*	OP050971
2018/7/3	SZ507	*Squaliobarbus curriculus*	OP050972
2018/7/3	SZ508	*Squaliobarbus curriculus*	OP050973
2018/7/3	SZ509	*Squaliobarbus curriculus*	OP050974
2018/7/3	SZ510	*Squaliobarbus curriculus*	OP050975
2018/7/3	SZ511	*Squaliobarbus curriculus*	OP050976
2018/7/3	SZ512	*Sinibotia robusta*	OP050757
2018/7/8	SZ513	*Oreochromis* sp. 2	NA
2018/7/8	SZ514	*Pseudohemiculter dispar*	OP050648
2018/7/8	SZ515	*Siniperca scherzeri*	OP050840
2018/7/8	SZ516	*Siniperca scherzeri*	OP050841
2018/7/8	SZ517	*Siniperca scherzeri*	OP050842
2018/7/8	SZ518	*Sinibotia robusta*	OP050758
2018/7/8	SZ519	*Squaliobarbus curriculus*	OP050977
2018/7/8	SZ520	*Pseudohemiculter dispar*	OP050649
2018/7/8	SZ521	*Pseudohemiculter dispar*	OP050650
2018/7/8	SZ522	*Siniperca* sp.	NA
2018/7/8	SZ523	*Pseudohemiculter dispar*	OP050651
2018/7/8	SZ524	*Chanodichthys recurviceps*	OP050480
2018/7/8	SZ525	*Pseudohemiculter dispar*	OP050652
2018/7/8	SZ526	*Squaliobarbus curriculus*	OP050978
2018/7/8	SZ527	*Squaliobarbus curriculus*	OP050979
2018/7/8	SZ528	*Pseudolaubuca engraulis*	OP050726
2018/7/8	SZ530	*Siniperca scherzeri*	OP050843
2018/7/8	SZ531	*Squaliobarbus curriculus*	OP050980
2018/7/8	SZ532	*Pseudohemiculter dispar*	OP050654
2018/7/8	SZ533	*Squaliobarbus curriculus*	OP050981
2018/7/8	SZ534	*Chanodichthys recurviceps*	OP050481
2018/7/8	SZ535	*Siniperca* sp.	NA
2018/7/8	SZ536	*Squaliobarbus curriculus*	OP050982
2018/7/8	SZ536	*Squaliobarbus curriculus*	OP050983
2018/7/8	SZ537	*Chanodichthys recurviceps*	OP050482
2018/7/8	SZ538	*Rhinogobius* sp. 4	NA
2018/7/8	SZ539	*Chanodichthys recurviceps*	OP050483
2018/7/8	SZ540	*Chanodichthys recurviceps*	OP050484
2018/7/8	SZ541	*Chanodichthys recurviceps*	OP050485
2018/7/8	SZ542	*Squaliobarbus curriculus*	OP050984
2018/7/8	SZ543	*Siniperca* sp.	NA
2018/7/8	SZ544	*Siniperca* sp.	NA

2018/7/8	SZ545	*Siniperca* sp.	NA
2018/7/8	SZ546	*Pseudohemiculter dispar*	OP050656
2018/7/8	SZ547	*Squaliobarbus curriculus*	OP050985
2018/7/8	SZ548	*Chanodichthys* sp.	NA
2018/7/8	SZ549	*Chanodichthys recurviceps*	OP050486
2018/7/8	SZ550	*Squalidus argentatus*	OP050862
2018/7/8	SZ551	*Hemiculter leucisculus*	OP050540
2018/7/8	SZ552	*Pseudohemiculter dispar*	OP050658
2018/7/8	SZ553	*Chanodichthys recurviceps*	OP050487
2018/7/8	SZ554	*Chanodichthys recurviceps*	OP050488
2018/7/8	SZ555	*Squaliobarbus curriculus*	OP050986
2018/7/8	SZ557	*Squaliobarbus curriculus*	OP050987
2018/7/8	SZ558	*Siniperca* sp.	NA
2018/7/8	SZ559	*Pseudohemiculter dispar*	OP050659
2018/7/8	SZ560	*Squaliobarbus curriculus*	OP050988
2018/7/8	SZ561	*Pseudohemiculter dispar*	OP050661
2018/7/8	SZ562	*Squalidus argentatus*	OP050863
2018/7/8	SZ564	*Siniperca* sp.	NA
2018/7/8	SZ565	*Siniperca* sp.	NA
2018/7/8	SZ566	*Pseudohemiculter dispar*	OP050662
2018/7/8	SZ567	*Pseudohemiculter dispar*	OP050663
2018/7/8	SZ568	*Siniperca* sp.	NA
2018/7/8	SZ569	*Squaliobarbus curriculus*	OP050989
2018/7/8	SZ570	*Siniperca* sp.	NA
2018/7/8	SZ571	*Chanodichthys recurviceps*	OP050489
2018/7/8	SZ572	*Chanodichthys recurviceps*	OP050490
2018/7/8	SZ573	*Pseudolaubuca engraulis*	OP050727
2018/7/8	SZ574	*Squaliobarbus curriculus*	OP050990
2018/7/8	SZ575	*Pseudohemiculter dispar*	OP050665
2018/7/8	SZ576	*Pseudohemiculter dispar*	OP050666
2018/7/8	SZ577	*Rhinogobius* sp. 4	NA
2018/7/8	SZ578	*Pseudohemiculter dispar*	OP050667
2018/7/8	SZ579	*Pseudohemiculter dispar*	OP050668
2018/7/8	SZ580	*Chanodichthys recurviceps*	OP050491
2018/7/8	SZ581	*Sinibrama macrops*	OP050799
2018/7/8	SZ582	*Chanodichthys recurviceps*	OP050492
2018/7/8	SZ583	*Pseudohemiculter dispar*	OP050670
2018/7/8	SZ584	*Pseudohemiculter dispar*	OP050671
2018/7/8	SZ585	*Squaliobarbus curriculus*	OP050991
2018/7/8	SZ586	*Chanodichthys recurviceps*	OP050493
2018/7/8	SZ587	*Chanodichthys recurviceps*	OP050494
2018/7/8	SZ588	*Chanodichthys recurviceps*	OP050495
2018/7/8	SZ589	*Pseudohemiculter dispar*	OP050672
2018/7/8	SZ590	*Pseudohemiculter dispar*	OP050674

2018/7/8	SZ591	*Squaliobarbus curriculus*	OP050992
2018/7/8	SZ592	*Squaliobarbus curriculus*	OP050993
2018/7/8	SZ593	*Squalidus argentatus*	OP050864
2018/7/8	SZ594	*Pseudohemiculter dispar*	OP050675
2018/7/14	SZ595	*Hyporhamphus intermedius*	OP050556
2018/7/14	SZ597	*Chanodichthys recurviceps*	OP050496
2018/7/14	SZ598	*Squaliobarbus curriculus*	OP050994
2018/7/14	SZ599	*Pseudohemiculter dispar*	OP050676
2018/7/14	SZ600	*Pseudohemiculter dispar*	OP050678
2018/7/14	SZ601	*Squaliobarbus curriculus*	OP050995
2018/7/14	SZ602	*Squaliobarbus curriculus*	OP050996
2018/7/14	SZ603	*Siniperca* sp.	NA
2018/7/14	SZ604	*Chanodichthys recurviceps*	OP050497
2018/7/14	SZ605	*Pseudohemiculter dispar*	OP050679
2018/7/14	SZ606	*Siniperca scherzeri*	OP050844
2018/7/14	SZ607	*Siniperca scherzeri*	OP050845
2018/7/14	SZ608	*Siniperca* sp.	NA
2018/7/14	SZ609	*Chanodichthys recurviceps*	OP050498
2018/7/21	SZ610	*Hypostomus* sp.	NA
2018/7/21	SZ611	*Pseudolaubuca sinensis*	OP050736
2018/7/21	SZ612	*Sinibotia robusta*	OP050759
2018/7/21	SZ613	*Sinibotia robusta*	OP050760
2018/7/21	SZ614	*Sinibotia robusta*	OP050761
2018/7/21	SZ615	*Squaliobarbus curriculus*	OP050997
2018/7/21	SZ616	*Chanodichthys recurviceps*	OP050499
2018/7/21	SZ617	*Sinibotia robusta*	OP050762
2018/7/21	SZ618	*Mylopharyngodon piceus*	OP050569
2018/7/21	SZ619	*Sinibotia robusta*	OP050763
2018/7/21	SZ620	*Sinibotia robusta*	OP050764
2018/7/21	SZ621	*Squaliobarbus curriculus*	OP050998
2018/7/21	SZ622	*Sinibotia robusta*	OP050765
2018/7/21	SZ623	*Rhinogobius* sp. 3	NA
2018/7/21	SZ624	*Squaliobarbus curriculus*	OP050999
2018/7/21	SZ625	*Sinibotia robusta*	OP050766
2018/7/21	SZ626	*Rhinogobius* sp. 3	NA
2018/7/21	SZ627	*Sinibrama macrops*	OP050800
2018/7/21	SZ628	*Sinibotia robusta*	OP050767
2018/7/21	SZ629	*Sinibotia robusta*	OP050768
2018/7/21	SZ630	*Pseudolaubuca sinensis*	OP050737
2018/7/21	SZ631	*Rhinogobius* sp. 3	NA
2018/7/21	SZ632	*Sinibotia robusta*	OP050769
2018/7/21	SZ633	*Chanodichthys recurviceps*	OP050500
2018/7/21	SZ634	*Squaliobarbus curriculus*	OP051000
2018/7/21	SZ635	*Squaliobarbus curriculus*	OP051001

2018/7/21	SZ636	*Squaliobarbus curriculus*	OP051002
2018/7/21	SZ637	*Chanodichthys recurviceps*	OP050501
2018/7/21	SZ638	*Squalidus argentatus*	OP050865
2018/7/21	SZ639	*Squaliobarbus curriculus*	OP051003
2018/7/26	SZ641	*Sinibotia robusta*	OP050770
2018/7/26	SZ642	*Sinibotia robusta*	OP050771
2018/7/26	SZ643	*Sinibotia robusta*	OP050772
2018/7/26	SZ644	*Sinibotia robusta*	OP050773
2018/7/26	SZ645	*Sinibotia robusta*	OP050774
2018/7/26	SZ646	*Squaliobarbus curriculus*	OP051004
2018/7/26	SZ647	*Sinibotia robusta*	OP050775
2018/7/26	SZ648	*Squaliobarbus curriculus*	OP051005
2018/7/26	SZ649	*Sinibotia robusta*	OP050776
2018/7/26	SZ650	*Sinibotia robusta*	OP050777
2018/7/26	SZ651	*Squaliobarbus curriculus*	OP051006
2018/7/26	SZ652	*Sinibotia robusta*	OP050778
2018/7/26	SZ653	*Sinibotia robusta*	OP050779
2018/7/26	SZ654	*Siniperca* sp.	NA
2018/7/26	SZ655	*Siniperca scherzeri*	OP050846
2018/7/26	SZ657	*Sinibotia robusta*	OP050780
2018/7/26	SZ658	*Siniperca* sp.	NA
2018/7/26	SZ659	*Squaliobarbus curriculus*	OP051007
2018/7/26	SZ660	*Sinibotia robusta*	OP050781
2018/7/26	SZ661	*Pseudohemiculter dispar*	OP050684
2018/7/26	SZ662	*Sinibotia robusta*	OP050782
2018/7/26	SZ663	*Pseudohemiculter dispar*	OP050685
2018/7/26	SZ664	*Squaliobarbus curriculus*	OP051008
2018/7/26	SZ665	*Sinibotia robusta*	OP050783
2018/7/26	SZ666	*Squaliobarbus curriculus*	OP051009
2018/7/26	SZ667	*Squaliobarbus curriculus*	OP051010
2018/7/26	SZ668	*Sinibotia robusta*	OP050784
2018/7/26	SZ669	*Sinibotia robusta*	OP050785
2018/7/30	SZ670	*Rhinogobius* sp. 3	NA
2018/7/30	SZ671	*Siniperca scherzeri*	OP050847
2018/7/30	SZ672	*Sinibotia robusta*	OP050786
2018/7/30	SZ673	*Squaliobarbus curriculus*	OP051011
2018/7/30	SZ674	*Siniperca* sp.	NA
2018/7/30	SZ675	*Pseudolaubuca sinensis*	OP050738
2018/7/30	SZ676	*Siniperca scherzeri*	OP050848
2018/7/30	SZ677	*Pseudolaubuca sinensis*	OP050739
2018/7/30	SZ678	*Siniperca scherzeri*	OP050849
2018/7/30	SZ679	*Squalidus argentatus*	OP050866
2018/7/30	SZ680	*Chanodichthys recurviceps*	OP050502
2018/7/30	SZ681	*Siniperca* sp.	NA

2018/7/30	SZ682	*Chanodichthys recurviceps*	OP050503
2018/7/30	SZ683	*Sinibrama macrops*	OP050801
2018/7/30	SZ684	*Chanodichthys recurviceps*	OP050504
2018/7/30	SZ685	*Squaliobarbus curriculus*	OP051012
2018/7/30	SZ686	*Siniperca* sp.	NA
2018/7/30	SZ687	*Siniperca* sp.	NA
2018/7/30	SZ688	*Siniperca* sp.	NA
2018/7/30	SZ689	*Oreochromis* sp. 2	NA
2018/7/30	SZ690	*Siniperca scherzeri*	OP050850
2018/7/30	SZ691	*Squalidus argentatus*	OP050867
2018/7/30	SZ692	*Mugilogobius myxodermus*	OP050565
2018/8/3	SZ693	*Squaliobarbus curriculus*	OP051013
2018/8/3	SZ694	*Pseudohemiculter dispar*	OP050689
2018/8/3	SZ695	*Pseudohemiculter dispar*	OP050690
2018/8/3	SZ696	*Squaliobarbus curriculus*	OP051014
2018/8/3	SZ697	*Siniperca* sp.	NA
2018/8/3	SZ698	*Squaliobarbus curriculus*	OP051015
2018/8/3	SZ699	*Squaliobarbus curriculus*	OP051016
2018/8/3	SZ700	*Squaliobarbus curriculus*	OP051017
2018/8/3	SZ701	*Squaliobarbus curriculus*	OP051018
2018/8/3	SZ702	*Squaliobarbus curriculus*	OP051019
2018/8/3	SZ703	*Siniperca* sp.	NA
2018/8/3	SZ704	*Squaliobarbus curriculus*	OP051020
2018/8/3	SZ705	*Pseudohemiculter dispar*	OP050691
2018/8/3	SZ706	*Squaliobarbus curriculus*	OP051021
2018/8/3	SZ707	*Squaliobarbus curriculus*	OP051022
2018/8/3	SZ708	*Pseudolaubuca sinensis*	OP050730
2018/8/3	SZ709	*Squaliobarbus curriculus*	OP051023
2018/8/3	SZ710	*Pseudohemiculter dispar*	OP050693
2018/8/3	SZ711	*Squaliobarbus curriculus*	OP051024
2018/8/3	SZ712	*Siniperca* sp.	NA
2018/8/3	SZ713	*Chanodichthys recurviceps*	OP050505
2018/8/3	SZ714	*Squaliobarbus curriculus*	OP051025
2018/8/3	SZ715	*Squaliobarbus curriculus*	OP051026
2018/8/3	SZ716	*Squaliobarbus curriculus*	OP051027
2018/8/3	SZ717	*Squaliobarbus curriculus*	OP051028
2018/8/3	SZ718	*Squaliobarbus curriculus*	OP051029
2018/8/3	SZ719	*Squaliobarbus curriculus*	OP051030
2018/8/3	SZ720	*Squaliobarbus curriculus*	OP051031
2018/8/3	SZ721	*Squaliobarbus curriculus*	OP051032
2018/8/3	SZ722	*Pseudohemiculter dispar*	OP050694
2018/8/3	SZ723	*Pseudohemiculter dispar*	OP050695
2018/8/3	SZ724	*Rhinogobius* sp. 4	NA
2018/8/3	SZ725	*Squaliobarbus curriculus*	OP051033

2018/8/3	SZ726	*Squaliobarbus curriculus*	OP051034
2018/8/3	SZ727	*Pseudolaubuca engraulis*	OP050728
2018/8/3	SZ728	*Pseudohemiculter dispar*	OP050696
2018/8/3	SZ729	*Squaliobarbus curriculus*	OP051035
2018/8/10	SZ1214	*Pseudolaubuca engraulis*	OP050717
2018/8/10	SZ1773	*Hemiculter leucisculus*	OP050530
2018/8/14	SZ1965	*Pseudohemiculter dispar*	OP050598
2018/8/14	SZ1992	*Siniperca* sp.	NA
2018/8/14	SZ2011	*Neosalanx* sp.	NA
2018/8/14	SZ2027	*Pseudohemiculter dispar*	OP050601
2018/8/14	SZ2032	*Squaliobarbus curriculus*	OP050869
2018/8/14	SZ2038	*Neosalanx* sp.	NA
2018/8/18	SZ2668	*Siniperca scherzeri*	OP050821
2018/8/18	SZ2675	*Siniperca* sp.	NA
2018/8/18	SZ2676	*Siniperca scherzeri*	OP050822
2018/8/18	SZ2686	*Siniperca* sp.	NA
2018/8/18	SZ2687	*Siniperca* sp.	NA
2018/8/18	SZ2689	*Siniperca scherzeri*	OP050823
2018/8/18	SZ2690	*Siniperca scherzeri*	OP050824
2018/8/18	SZ2695	*Siniperca* sp.	NA
2018/8/18	SZ2697	*Siniperca scherzeri*	OP050825
2018/8/18	SZ2698	*Siniperca scherzeri*	OP050826
2018/8/18	SZ2701	*Siniperca* sp.	NA
2018/8/22	SZ2714	*Chanodichthys* sp.	NA
2018/8/22	SZ2718	*Squaliobarbus curriculus*	OP050870
2018/8/22	SZ2721	*Squaliobarbus curriculus*	OP050871
2018/8/22	SZ2732	*Pseudohemiculter dispar*	OP050601
2018/8/22	SZ2736	*Rhinogobius* sp. 4	NA
2018/8/22	SZ2738	*Siniperca* sp.	NA
2018/8/22	SZ2740	*Siniperca scherzeri*	OP050827
2018/8/22	SZ2741	*Siniperca* sp.	NA
2018/8/22	SZ2742	*Squaliobarbus curriculus*	OP050872
2018/8/22	SZ2750	*Siniperca* sp.	NA
2018/8/22	SZ2755	*Schistura* sp.	NA
2018/8/22	SZ2756	*Hemiculterella sauvagei*	OP050544
2018/8/22	SZ2758	*Neosalanx* sp.	NA
2018/8/22	SZ2760	*Siniperca* sp.	NA
2018/8/22	SZ2761	*Rhinogobius* sp. 4	NA
2018/8/22	SZ2762	*Squaliobarbus curriculus*	OP050873
2018/8/22	SZ2767	*Siniperca scherzeri*	OP050829
2018/8/22	SZ2769	*Siniperca* sp.	NA
2018/8/27	SZ2792	*Ancherythroculter lini*	OP050461
2018/8/27	SZ2793	*Squaliobarbus curriculus*	OP050874
2018/8/27	SZ2795	*Pseudohemiculter dispar*	OP050615

2018/8/27	SZ2796	*Squaliobarbus curriculus*	OP050875
2018/8/27	SZ2798	*Squaliobarbus curriculus*	OP050876
2018/8/27	SZ2801	*Ancherythroculter lini*	OP050462
2018/8/27	SZ2802	*Chanodichthys recurviceps*	OP050473
2018/8/27	SZ2803	*Pseudohemiculter dispar*	OP050617
2018/8/27	SZ2804	*Rhinogobius* sp. 4	NA
2018/8/27	SZ2805	*Squaliobarbus curriculus*	OP050877
2018/8/27	SZ2806	*Squaliobarbus curriculus*	OP050878
2018/8/27	SZ2807	*Squaliobarbus curriculus*	OP050879
2018/8/27	SZ2812	*Siniperca* sp.	NA
2018/8/27	SZ2813	*Chanodichthys recurviceps*	OP050474
2018/8/27	SZ2814	*Chanodichthys recurviceps*	OP050475
2018/8/27	SZ2815	*Pseudohemiculter dispar*	OP050618
2018/8/27	SZ2822	*Mugilogobius myxodermus*	OP050563
2018/8/27	SZ2841	*Siniperca* sp.	NA
2018/8/27	SZ2843	*Neosalanx* sp.	NA
2018/8/27	SZ2857	*Siniperca* sp.	NA
2018/8/27	SZ2867	*Sinibotia robusta*	OP050744
2018/8/27	SZ2871	*Sinibotia robusta*	OP050745
2018/8/27	SZ2874	*Sinibotia robusta*	OP050746
2018/8/27	SZ2882	*Rhinogobius* sp. 4	NA
2018/8/27	SZ2883	*Rhinogobius* sp. 4	NA
2018/8/27	SZ2886	*Sinibotia robusta*	OP050747
2018/8/27	SZ2943	*Siniperca* sp.	NA
2018/8/27	SZ2944	*Squaliobarbus curriculus*	OP050881
2018/8/27	SZ2949	*Squaliobarbus curriculus*	OP050882
2018/8/27	SZ2953	*Siniperca* sp.	NA
2018/8/27	SZ2954	*Squaliobarbus curriculus*	OP050883
2018/8/27	SZ2955	*Squaliobarbus curriculus*	OP050884
2018/8/27	SZ2956	*Squaliobarbus curriculus*	OP050885
2018/8/27	SZ2957	*Mugilogobius myxodermus*	OP050564
2018/8/27	SZ2959	*Pseudolaubuca sinensis*	OP050733
2018/8/27	SZ2960	*Pseudohemiculter dispar*	OP050620
2018/8/27	SZ2963	*Squaliobarbus curriculus*	OP050886
2018/8/27	SZ2964	*Squaliobarbus curriculus*	OP050887
2018/8/27	SZ2965	*Sinibotia robusta*	OP050748
2018/8/27	SZ2966	*Squaliobarbus curriculus*	OP050888
2018/8/27	SZ2967	*Siniperca* sp.	NA
2018/8/27	SZ2968	*Siniperca* sp.	NA
2018/8/27	SZ2969	*Squaliobarbus curriculus*	OP050889
2018/8/27	SZ2971	*Squaliobarbus curriculus*	OP050890
2018/8/27	SZ2972	*Siniperca* sp.	NA
2018/8/27	SZ2974	*Siniperca scherzeri*	OP050830
2018/8/27	SZ2976	*Squaliobarbus curriculus*	OP050891

2018/8/27	SZ2978	*Squaliobarbus curriculus*	OP050892
2018/8/27	SZ2979	*Siniperca* sp.	NA
2018/8/27	SZ2980	*Squaliobarbus curriculus*	OP050893
2018/8/27	SZ2984	*Siniperca* sp.	NA
