# Supplementary material for: Unraveling the Drifting Larval Fish Community in a Large Spawning Ground in the Middle Pearl River Using DNA Barcoding
Source: Animals (Basel). 2022 Sep 24;12(19):2555. doi: 10.3390/ani12192555 (PMC9559676; doi:10.3390/ani12192555)
Supplement: Supplementary file 1 [file animals-12-02555-s001.zip › Table S2.pdf]

Sample ID	Final name	Best match species	Genbank Nos	Simililarity	Nearest species	Genbank Nos	Simililarity
SZ1	*Coptodon zillii*	*Coptodon zillii*	MG407387	100	/	/	/
SZ2	*Hyporhamphus intermedius*	*Hyporhamphus intermedius*	MT805182	100	*Rhynchorhamphus georgii*	JQ738540	87.99
SZ3	*Siniperca* sp.	*Siniperca knerii*	MT805838	100	*Siniperca chuatsi*	private	100
SZ4	*Hyporhamphus intermedius*	*Hyporhamphus intermedius*	private	100	*Rhynchorhamphus georgii*	JQ738540	87.84
SZ5	*Hyporhamphus intermedius*	*Hyporhamphus intermedius*	MT805184	100	*Rhynchorhamphus georgii*	JQ738540	87.99
SZ6	*Hemiculter leucisculus*	*Hemiculter leucisculus*	MZ403354	100	/	/	/
SZ7	*Rhinogobius* sp. 4	*Rhinogobius sp. Ceheng*	private	98.26	*Rhinogobius sp. Liping*	private	91.79
SZ8	*Pseudohemiculter dispar*	*Pseudohemiculter dispar*	MZ403512	100	/	/	/
SZ9	*Siniperca* sp.	*Siniperca knerii*	MT805836	100	*Siniperca chuatsi*	MW402975	100
SZ10	*Siniperca scherzeri*	*Siniperca scherzeri*	MZ149211	100	*Siniperca roulei*	KP710957	96.42
SZ11	*Zacco platypus*	*Zacco platypus*	MZ149250	100	*Zacco acutipinnis*	NC028595	93.64
SZ12	*Siniperca* sp.	*Siniperca knerii*	MT805838	100	*Siniperca chuatsi*	private	100
SZ13	*Siniperca* sp.	*Siniperca knerii*	MT805838	100	*Siniperca chuatsi*	private	100
SZ14	*Siniperca* sp.	*Siniperca knerii*	MT805828	100	*Siniperca chuatsi*	KX224166	99.84
SZ15	*Opsariichthys bidens*	*Opsariichthys bidens*	MT805369	100	*Zacco acutipinnis*	NC028595	92.87
SZ16	*Zacco platypus*	*Zacco platypus*	MT806018	100	*Zacco acutipinnis*	NC028595	94.88
SZ17	*Pseudohemiculter dispar*	*Pseudohemiculter dispar*	MZ403505	100	/	/	/
SZ18	*Zacco platypus*	*Zacco platypus*	MT806018	100	*Zacco acutipinnis*	NC028595	94.88
SZ19	*Zacco platypus*	*Zacco platypus*	MZ149250	100	*Zacco acutipinnis*	NC028595	93.64
SZ20	*Pseudohemiculter dispar*	*Pseudohemiculter dispar*	MZ403505	100	/	/	/
SZ21	*Siniperca* sp.	*Siniperca knerii*	MT805838	100	*Siniperca chuatsi*	private	100
SZ22	*Siniperca* sp.	*Siniperca knerii*	MT805833	100	*Siniperca chuatsi*	KX224166	99.84
SZ24	*Zacco platypus*	*Zacco platypus*	MZ149250	100	*Zacco acutipinnis*	NC028595	93.64
SZ25	*Pseudohemiculter dispar*	*Pseudohemiculter dispar*	MZ403512	100	/	/	/
SZ26	*Siniperca* sp.	*Siniperca knerii*	MT805838	100	*Siniperca chuatsi*	private	100
SZ27	*Siniperca* sp.	*Siniperca knerii*	MT805838	100	*Siniperca chuatsi*	private	100
SZ28	*Pseudohemiculter dispar*	*Pseudohemiculter dispar*	MZ403505	100	/	/	/
SZ29	*Rhinogobius* sp. 3	*Rhinogobius giurinus*	NC022692	100	*Rhinogobius similis*	KF371534	100
SZ30	*Sinibrama macrops*	*Sinibrama macrops*	MZ403625	100	*Sinibrama wui*	private	96.35

SZ31	*Siniperca scherzeri*	*Siniperca scherzeri*	MZ149211	100	*Siniperca roulei*	KP710957	96.42
SZ32	*Siniperca* sp.	*Siniperca knerii*	MT805836	100	*Siniperca chuatsi*	MW402975	100
SZ33	*Siniperca scherzeri*	*Siniperca scherzeri*	MZ149211	100	*Siniperca roulei*	KP710957	96.42
SZ34	*Siniperca* sp.	*Siniperca knerii*	MT805838	100	*Siniperca chuatsi*	private	100
SZ35	*Siniperca scherzeri*	*Siniperca scherzeri*	MZ149211	100	*Siniperca roulei*	KP710957	96.42
SZ36	*Siniperca* sp.	*Siniperca knerii*	MT805838	100	*Siniperca chuatsi*	private	100
SZ37	*Siniperca* sp.	*Siniperca knerii*	MT805838	100	*Siniperca chuatsi*	private	100
SZ38	*Siniperca* sp.	*Siniperca knerii*	MT805833	100	*Siniperca chuatsi*	KX224166	99.84
SZ39	*Siniperca* sp.	*Siniperca knerii*	MT805836	100	*Siniperca chuatsi*	MW402975	100
SZ40	*Siniperca* sp.	*Siniperca chuatsi*	KX224166	99.84	*Siniperca knerii*	EF143389	99.84
SZ41	*Siniperca* sp.	*Siniperca knerii*	MT805838	100	*Siniperca chuatsi*	private	100
SZ42	*Pseudohemiculter dispar*	*Pseudohemiculter dispar*	MZ403505	100	/	/	/
SZ43	*Siniperca* sp.	*Siniperca knerii*	MT805838	100	*Siniperca chuatsi*	private	100
SZ44	*Zacco platypus*	*Zacco platypus*	MZ149250	100	*Zacco acutipinnis*	NC028595	93.64
SZ45	*Hemiculter leucisculus*	*Hemiculter leucisculus*	MZ403310	100	/	/	/
SZ46	*Pseudolaubuca engraulis*	*Pseudolaubuca engraulis*	MZ403563	100	/	/	/
SZ47	*Pseudohemiculter dispar*	*Pseudohemiculter dispar*	MZ403505	100	/	/	/
SZ48	*Pseudohemiculter dispar*	*Pseudohemiculter dispar*	MZ403505	100	/	/	/
SZ49	*Pseudohemiculter dispar*	*Pseudohemiculter dispar*	MZ403505	100	/	/	/
SZ50	*Rhinogobius* sp. 1	*Rhinogobius leavelli*	AB988820	100	*Rhinogobius maculafasciatus*	KU944927	99.46
SZ51	*Pseudohemiculter dispar*	*Pseudohemiculter dispar*	MZ403505	100	/	/	/
SZ52	*Rhinogobius* sp. 4	*Rhinogobius sp. Ceheng*	private	98.26	*Rhinogobius sp. Liping*	private	91.79
SZ53	*Pseudohemiculter dispar*	*Pseudohemiculter dispar*	MZ403505	100	/	/	/
SZ54	*Pseudohemiculter dispar*	*Pseudohemiculter dispar*	MZ403512	100	/	/	/
SZ55	*Pseudohemiculter dispar*	*Pseudohemiculter dispar*	MZ403515	100	/	/	/
SZ56	*Pseudohemiculter dispar*	*Pseudohemiculter dispar*	MZ403505	100	/	/	/
SZ57	*Pseudohemiculter dispar*	*Pseudohemiculter dispar*	MZ403515	100	/	/	/
SZ58	*Pseudohemiculter dispar*	*Pseudohemiculter dispar*	MZ403505	100	/	/	/
SZ59	*Pseudohemiculter dispar*	*Pseudohemiculter dispar*	MZ403505	100	/	/	/

SZ60	*Pseudohemiculter dispar*	*Pseudohemiculter dispar*	MZ148927	100	/	/	/
SZ61	*Siniperca* sp.	*Siniperca knerii*	MT805838	100	*Siniperca chuatsi*	private	100
SZ62	*Pseudohemiculter dispar*	*Pseudohemiculter dispar*	MZ403505	100	/	/	/
SZ63	*Pseudohemiculter dispar*	*Pseudohemiculter dispar*	MZ403505	100	/	/	/
SZ64	*Hemiculter leucisculus*	*Hemiculter leucisculus*	MZ403354	100	/	/	/
SZ65	*Pseudohemiculter dispar*	*Pseudohemiculter dispar*	MZ403505	100	/	/	/
SZ66	*Pseudohemiculter dispar*	*Pseudohemiculter dispar*	private	99.68	/	/	/
SZ67	*Pseudohemiculter dispar*	*Pseudohemiculter dispar*	MZ403515	100	/	/	/
SZ68	*Pseudohemiculter dispar*	*Pseudohemiculter dispar*	MZ403505	100	/	/	/
SZ69	*Pseudohemiculter dispar*	*Pseudohemiculter dispar*	MZ403515	100	/	/	/
SZ70	*Rhinogobius* sp. 1	*Rhinogobius leavelli*	AB988820	99.84	*Rhinogobius maculafasciatus*	KU944927	99.28
SZ71	*Pseudohemiculter dispar*	*Pseudohemiculter dispar*	MZ403505	100	/	/	/
SZ72	*Hemiculter leucisculus*	*Hemiculter leucisculus*	MZ403310	100	/	/	/
SZ73	*Pseudohemiculter dispar*	*Pseudohemiculter dispar*	MZ403505	100	/	/	/
SZ74	*Siniperca scherzeri*	*Siniperca scherzeri*	MZ149211	100	*Siniperca roulei*	KP710957	96.42
SZ75	*Coilia grayii*	*Coilia grayii*	KX254516	100	*Coilia nasus*	KY038985	97.79
SZ76	*Pseudolaubuca engraulis*	*Pseudolaubuca engraulis*	MZ403565	100	/	/	/
SZ77	*Pseudohemiculter dispar*	*Pseudohemiculter dispar*	MZ403505	100	/	/	/
SZ78	*Siniperca scherzeri*	*Siniperca scherzeri*	MZ149211	100	*Siniperca roulei*	KP710957	96.42
SZ79	*Pseudohemiculter dispar*	*Pseudohemiculter dispar*	MZ403505	100	/	/	/
SZ80	*Pseudohemiculter dispar*	*Pseudohemiculter dispar*	MZ403515	100	/	/	/
SZ81	*Hemiculterella sauvagei*	*Hemiculterella sauvagei*	MZ403261	100	*Hemiculter leucisculus*	private	95.73
SZ82	*Zacco platypus*	*Zacco platypus*	private	99.83	*Zacco acutipinnis*	NC028595	93.49
SZ83	*Pseudohemiculter dispar*	*Pseudohemiculter dispar*	MZ403505	100	/	/	/
SZ84	*Pseudohemiculter dispar*	*Pseudohemiculter dispar*	MZ403505	100	/	/	/
SZ85	*Pseudohemiculter dispar*	*Pseudohemiculter dispar*	MZ403505	100	/	/	/
SZ86	*Pseudohemiculter dispar*	*Pseudohemiculter dispar*	MZ403505	100	/	/	/
SZ87	*Pseudohemiculter dispar*	*Pseudohemiculter dispar*	MZ403505	100	/	/	/
SZ88	*Pseudohemiculter dispar*	*Pseudohemiculter dispar*	MZ403505	100	/	/	/

SZ89	*Pseudohemiculter dispar*	*Pseudohemiculter dispar*	MZ403505	100	/	/	/
SZ90	*Zacco platypus*	*Zacco platypus*	MZ149250	100	*Zacco acutipinnis*	NC028595	93.64
SZ91	*Pseudohemiculter dispar*	*Pseudohemiculter dispar*	MZ403505	100	/	/	/
SZ92	*Rhinogobius* sp. 1	*Rhinogobius leavelli*	AB988820	100	*Rhinogobius maculafasciatus*	KU944927	99.46
SZ93	*Pseudohemiculter dispar*	*Pseudohemiculter dispar*	MZ403505	100	/	/	/
SZ94	*Pseudohemiculter dispar*	*Pseudohemiculter dispar*	MZ403505	100	/	/	/
SZ95	*Pseudohemiculter dispar*	*Pseudohemiculter dispar*	MZ403505	100	/	/	/
SZ96	*Pseudohemiculter dispar*	*Pseudohemiculter dispar*	private	99.84	/	/	/
SZ97	*Pseudohemiculter dispar*	*Pseudohemiculter dispar*	MZ403505	100	/	/	/
SZ98	*Pseudohemiculter dispar*	*Pseudohemiculter dispar*	MZ403505	100	/	/	/
SZ99	*Pseudohemiculter dispar*	*Pseudohemiculter dispar*	MZ403505	100	/	/	/
SZ100	*Pseudohemiculter dispar*	*Pseudohemiculter dispar*	MZ403505	100	/	/	/
SZ101	*Pseudohemiculter dispar*	*Pseudohemiculter dispar*	MZ148926	100	/	/	/
SZ102	*Pseudohemiculter dispar*	*Pseudohemiculter dispar*	MZ403505	100	/	/	/
SZ103	*Coilia grayii*	*Coilia grayii*	KX254516	100	*Coilia nasus*	KY038985	97.79
SZ104	*Rhinogobius* sp. 1	*Rhinogobius leavelli*	AB988820	100	*Rhinogobius maculafasciatus*	KU944927	99.46
SZ105	*Rhinogobius* sp. 1	*Rhinogobius leavelli*	AB988820	100	*Rhinogobius maculafasciatus*	KU944927	99.46
SZ106	*Mugilogobius myxodermus*	*Mugilogobius myxodermus*	MT805276	100	*Mugilogobius abei*	KF128984	90.85
SZ107	*Rhinogobius* sp. 1	*Rhinogobius leavelli*	AB988820	100	*Rhinogobius maculafasciatus*	KU944927	99.46
SZ108	*Coilia grayii*	*Coilia grayii*	KX254516	100	*Coilia nasus*	KY038985	97.79
SZ109	*Siniperca scherzeri*	*Siniperca scherzeri*	MZ149211	100	*Siniperca roulei*	KP710957	96.42
SZ110	*Chanodichthys recurviceps*	*Chanodichthys recurviceps*	MZ403103	100	/	/	/
SZ111	*Mugilogobius myxodermus*	*Mugilogobius myxodermus*	MT805275	99.82	*Mugilogobius abei*	KF128984	91.16
SZ113	*Chanodichthys recurviceps*	*Chanodichthys recurviceps*	MZ403103	100	/	/	/
SZ114	*Coilia grayii*	*Coilia grayii*	KX254516	100	*Coilia nasus*	KY038985	97.79
SZ115	*Rhinogobius* sp. 2	*Rhinogobius maculafasciatus*	KU944927	100	*Rhinogobius cliffordpopei*	MT413342	99.84
SZ116	*Mugilogobius myxodermus*	*Mugilogobius myxodermus*	MT805276	100	*Mugilogobius abei*	KF128984	90.85
SZ117	*Hyporhamphus intermedius*	*Hyporhamphus intermedius*	MT805180	100	*Rhynchorhamphus georgii*	JQ738540	87.99
SZ118	*Zacco platypus*	*Zacco platypus*	MZ149250	100	*Zacco acutipinnis*	NC028595	93.64

SZ119	*Chanodichthys recurviceps*	*Chanodichthys recurviceps*	MZ403103	100	/	/	/
SZ120	*Pseudohemiculter dispar*	*Pseudohemiculter dispar*	MZ403505	100	/	/	/
SZ121	*Siniperca scherzeri*	*Siniperca scherzeri*	MZ149211	100	*Siniperca roulei*	KP710957	96.42
SZ122	*Chanodichthys recurviceps*	*Chanodichthys recurviceps*	MZ403103	100	/	/	/
SZ123	*Megalobrama terminalis*	*Megalobrama terminalis*	MZ403461	100	/	/	/
SZ124	*Sicyopterus sp.*	*Sicyopterus sp.*	private	99.84	*Alepes melanoptera*	private	84.55
SZ125	*Pseudohemiculter dispar*	*Pseudohemiculter dispar*	MZ403505	100	/	/	/
SZ126	*Siniperca* sp.	*Siniperca knerii*	MT805838	100	*Siniperca chuatsi*	private	100
SZ127	*Coilia grayii*	*Coilia grayii*	KX254516	100	*Coilia nasus*	KY038985	97.79
SZ128	*Chanodichthys recurviceps*	*Chanodichthys recurviceps*	MZ403011	100	/	/	/
SZ129	*Rhinogobius* sp. 3	*Rhinogobius giurinus*	NC022692	100	*Rhinogobius similis*	KF371534	100
SZ130	*Squalidus argentatus*	*Squalidus argentatus*	MT805840	100	/	/	/
SZ131	*Squalidus argentatus*	*Squalidus argentatus*	MZ149227	100	/	/	/
SZ132	*Squalidus argentatus*	*Squalidus argentatus*	MZ149241	100	/	/	/
SZ133	*Chanodichthys recurviceps*	*Chanodichthys recurviceps*	MZ403011	100	/	/	/
SZ134	*Squalidus argentatus*	*Squalidus argentatus*	MT805840	100	/	/	/
SZ135	*Rhinogobius* sp. 1	*Rhinogobius leavelli*	AB988820	100	*Rhinogobius maculafasciatus*	KU944927	99.46
SZ136	*Rhinogobius* sp. 4	*Rhinogobius sp. Ceheng*	private	98.26	*Rhinogobius sp. Liping*	private	91.79
SZ137	*Pseudohemiculter dispar*	*Pseudohemiculter dispar*	MZ403505	100	/	/	/
SZ138	*Siniperca scherzeri*	*Siniperca scherzeri*	MZ149211	100	*Siniperca roulei*	KP710957	96.42
SZ139	*Chanodichthys recurviceps*	*Chanodichthys recurviceps*	MZ403103	100	/	/	/
SZ140	*Rhinogobius* sp. 1	*Rhinogobius leavelli*	AB988820	100	*Rhinogobius maculafasciatus*	KU944927	99.46
SZ141	*Oreochromis* sp. 1	*Oreochromis aureus*	MT525047	100	*Oreochromis niloticus*	private	100
SZ142	*Oreochromis* sp. 1	*Oreochromis aureus*	MT525047	100	*Oreochromis niloticus*	private	100
SZ143	*Siniperca scherzeri*	*Siniperca scherzeri*	private	99.84	/	/	/
SZ144	*Siniperca scherzeri*	*Siniperca scherzeri*	MZ149211	100	*Siniperca roulei*	KP710957	96.42
SZ145	*Siniperca scherzeri*	*Siniperca scherzeri*	MZ149211	100	*Siniperca roulei*	KP710957	96.42
SZ146	*Siniperca scherzeri*	*Siniperca scherzeri*	MZ149211	100	*Siniperca roulei*	KP710957	96.42
SZ147	*Hyporhamphus intermedius*	*Hyporhamphus intermedius*	MT805181	100	*Rhynchorhamphus georgii*	JQ738540	87.99

SZ148	*Pseudohemiculter dispar*	*Pseudohemiculter dispar*	MZ403505	100	/	/	/
SZ149	*Siniperca scherzeri*	*Siniperca scherzeri*	MZ149211	100	*Siniperca roulei*	KP710957	96.42
SZ150	*Siniperca scherzeri*	*Siniperca scherzeri*	MZ149211	100	*Siniperca roulei*	KP710957	96.42
SZ151	*Zacco platypus*	*Zacco platypus*	MZ149250	100	*Zacco acutipinnis*	NC028595	93.64
SZ152	*Pseudolaubuca engraulis*	*Pseudolaubuca engraulis*	MZ403565	100	/	/	/
SZ153	*Hemiculter leucisculus*	*Hemiculter leucisculus*	MZ403354	100	/	/	/
SZ154	*Pseudohemiculter dispar*	*Pseudohemiculter dispar*	MZ403505	100	/	/	/
SZ155	*Pseudohemiculter dispar*	*Pseudohemiculter dispar*	MZ403505	100	/	/	/
SZ156	*Pseudohemiculter dispar*	*Pseudohemiculter dispar*	MZ403505	100	/	/	/
SZ157	*Pseudohemiculter dispar*	*Pseudohemiculter dispar*	MZ403505	100	/	/	/
SZ158	*Hemiculter leucisculus*	*Hemiculter leucisculus*	MZ403353	99.83	/	/	/
SZ159	*Pseudohemiculter dispar*	*Pseudohemiculter dispar*	MZ403505	100	/	/	/
SZ160	*Zacco platypus*	*Zacco platypus*	MZ149250	100	*Zacco acutipinnis*	NC028595	93.64
SZ161	*Pseudohemiculter dispar*	*Pseudohemiculter dispar*	MZ403505	100	/	/	/
SZ162	*Pseudohemiculter dispar*	*Pseudohemiculter dispar*	MZ403505	100	/	/	/
SZ163	*Pseudohemiculter dispar*	*Pseudohemiculter dispar*	MZ403515	100	/	/	/
SZ164	*Hemiculter leucisculus*	*Hemiculter leucisculus*	MZ403354	100	/	/	/
SZ165	*Pseudohemiculter dispar*	*Pseudohemiculter dispar*	MZ403505	100	/	/	/
SZ166	*Pseudohemiculter dispar*	*Pseudohemiculter dispar*	MZ403505	100	/	/	/
SZ167	*Pseudohemiculter dispar*	*Pseudohemiculter dispar*	MZ403512	100	/	/	/
SZ168	*Pseudohemiculter dispar*	*Pseudohemiculter dispar*	MZ403505	100	/	/	/
SZ169	*Pseudohemiculter dispar*	*Pseudohemiculter dispar*	MZ403505	100	/	/	/
SZ170	*Pseudohemiculter dispar*	*Pseudohemiculter dispar*	MZ403505	100	/	/	/
SZ171	*Siniperca scherzeri*	*Siniperca scherzeri*	MZ149211	100	*Siniperca roulei*	KP710957	96.42
SZ172	*Pseudolaubuca sinensis*	*Pseudolaubuca sinensis*	MZ403617	100	/	/	/
SZ173	*Zacco platypus*	*Zacco platypus*	MT806018	100	*Zacco acutipinnis*	NC028595	94.88
SZ174	*Siniperca scherzeri*	*Siniperca scherzeri*	MZ149211	100	*Siniperca roulei*	KP710957	96.42
SZ175	*Hemiculter leucisculus*	*Hemiculter leucisculus*	MZ403321	100	/	/	/
SZ176	*Pseudohemiculter dispar*	*Pseudohemiculter dispar*	MZ403515	100	/	/	/

SZ177	Unknown sp.ecies 1	*NA*	*NA*	*NA*	*NA*	*NA*	*NA*
SZ178	*Pseudohemiculter dispar*	*Pseudohemiculter dispar*	MZ403515	100	/	/	/
SZ179	*Rhinogobius* sp. 2	*Rhinogobius maculafasciatus*	KU944928	100	*Rhinogobius cliffordpopei*	MT413343	99.84
SZ180	*Pseudohemiculter dispar*	*Pseudohemiculter dispar*	MZ403512	100	/	/	/
SZ181	*Neosalanx* sp.	*Neosalanx tangkahkeii*	GQ848972	100	*Neosalanx brevirostris*	private	100
SZ182	*Coilia grayii*	*Coilia grayii*	KX254516	100	*Coilia nasus*	KY038985	97.79
SZ183	*Oreochromis* sp. 1	*Oreochromis aureus*	MT525047	100	*Oreochromis niloticus*	private	100
SZ185	*Acheilognathinae*	*Acheilognathus tonkinensis*	NC042407	99.84	*Rhodeus sericeus*	HQ557338	99.52
SZ186	*Siniperca* sp.	*Siniperca chuatsi*	KX224166	99.84	*Siniperca knerii*	EF143389	99.84
SZ187	*Coilia grayii*	*Coilia grayii*	KX254516	100	*Coilia nasus*	KY038985	97.79
SZ188	*Sinibrama macrops*	*Sinibrama macrops*	MZ403625	100	*Sinibrama wui*	private	96.35
SZ189	Unknown sp.ecies 2	*NA*	*NA*	*NA*	*NA*	*NA*	*NA*
SZ190	*Sinibrama macrops*	*Sinibrama macrops*	MZ403625	100	*Sinibrama wui*	private	96.35
SZ191	*Zacco platypus*	*Zacco platypus*	MZ149250	100	*Zacco acutipinnis*	NC028595	93.64
SZ193	*Chanodichthys recurviceps*	*Chanodichthys recurviceps*	MZ403103	100	/	/	/
SZ194	*Zacco platypus*	*Zacco platypus*	MZ149250	100	*Zacco acutipinnis*	NC028595	93.64
SZ195	*Siniperca scherzeri*	*Siniperca scherzeri*	MZ149211	100	*Siniperca roulei*	KP710957	96.42
SZ196	*Rhinogobius* sp. 4	*Rhinogobius sp. Ceheng*	private	98.26	*Rhinogobius sp. Liping*	private	91.79
SZ197	*Oreochromis* sp. 1	*Oreochromis aureus*	MT525047	100	*Oreochromis niloticus*	private	100
SZ198	*Sinibotia robusta*	*Sinibotia robusta*	AP011436	100	/	/	/
SZ200	*Zacco platypus*	*Zacco platypus*	MZ149250	100	*Zacco acutipinnis*	NC028595	93.64
SZ201	*Pseudohemiculter dispar*	*Pseudohemiculter dispar*	private	99.68	/	/	/
SZ202	*Sinibrama macrops*	*Sinibrama macrops*	MZ403625	100	*Sinibrama wui*	private	96.35
SZ203	*Siniperca* sp.	*Siniperca knerii*	MT805836	100	*Siniperca chuatsi*	MW402975	100
SZ204	*Sinibrama macrops*	*Sinibrama macrops*	MZ403625	100	*Sinibrama wui*	private	96.35
SZ205	*Rhinogobius* sp. 3	*Rhinogobius giurinus*	private	100	*Rhinogobius similis*	MT805715	100
SZ206	*Mugilogobius myxodermus*	*Mugilogobius myxodermus*	MT805276	100	*Mugilogobius abei*	KF128984	90.85
SZ207	*Zacco platypus*	*Zacco platypus*	MZ149250	100	*Zacco acutipinnis*	NC028595	93.64
SZ208	*Mugilogobius myxodermus*	*Mugilogobius myxodermus*	MT805276	100	*Mugilogobius abei*	KF128984	90.85

SZ209	*Rhinogobius* sp. 1	*Rhinogobius leavelli*	AB988820	100	*Rhinogobius maculafasciatus*	KU944927	99.46
SZ210	*Sicyopterus* sp.	*Sicyopterus sp.*	private	99.84	*Alepes melanoptera*	private	84.55
SZ211	*Pseudohemiculter dispar*	*Pseudohemiculter dispar*	MZ403505	100	/	/	/
SZ212	*Pseudohemiculter dispar*	*Pseudohemiculter dispar*	MZ403515	100	/	/	/
SZ215	*Sinibrama macrops*	*Sinibrama macrops*	MZ403625	100	*Sinibrama wui*	private	96.35
SZ218	*Squalidus argentatus*	*Squalidus argentatus*	MZ149246	100	*Squalidus chankaensis*	private	98.89
SZ219	*Zacco platypus*	*Zacco platypus*	MZ149250	100	*Zacco acutipinnis*	NC028595	93.64
SZ220	*Rhinogobius* sp. 4	*Rhinogobius sp. Ceheng*	private	98.26	*Rhinogobius sp. Liping*	private	91.79
SZ221	*Siniperca scherzeri*	*Siniperca scherzeri*	MZ149211	100	*Siniperca roulei*	KP710957	96.42
SZ222	*Zacco platypus*	*Zacco platypus*	MT806018	100	*Zacco acutipinnis*	NC028595	94.88
SZ223	*Hemiculter leucisculus*	*Hemiculter leucisculus*	MZ403310	100	/	/	/
SZ224	*Rhinogobius* sp. 1	*Rhinogobius leavelli*	AB988820	100	*Rhinogobius maculafasciatus*	KU944927	99.46
SZ225	*Siniperca* sp.	*Siniperca knerii*	MT805838	100	*Siniperca chuatsi*	private	100
SZ226	*Siniperca* sp.	*Siniperca knerii*	MT805838	100	*Siniperca chuatsi*	private	100
SZ227	*Rhinogobius* sp. 4	*Rhinogobius sp. Ceheng*	private	98.26	*Rhinogobius sp. Liping*	private	91.79
SZ229	*Pseudohemiculter dispar*	*Pseudohemiculter dispar*	MZ403505	100	/	/	/
SZ230	*Rhinogobius* sp. 1	*Rhinogobius leavelli*	AB988820	100	*Rhinogobius maculafasciatus*	KU944927	99.46
SZ231	*Chanodichthys recurviceps*	*Chanodichthys recurviceps*	MZ403103	100	/	/	/
SZ232	*Siniperca scherzeri*	*Siniperca scherzeri*	MZ149211	100	*Siniperca roulei*	KP710957	96.42
SZ233	*Siniperca* sp.	*Siniperca knerii*	MT805838	100	*Siniperca chuatsi*	private	100
SZ234	*Sinibrama macrops*	*Sinibrama macrops*	MZ403625	100	*Sinibrama wui*	private	96.35
SZ235	*Hyporhamphus intermedius*	*Hyporhamphus intermedius*	MT805183	100	*Rhynchorhamphus georgii*	JQ738540	87.99
SZ236	*Sinibotia robusta*	*Sinibotia robusta*	MH027663	100	/	/	/
SZ237	*Opsariichthys bidens*	*Opsariichthys bidens*	MT805369	100	*Zacco acutipinnis*	NC028595	92.87
SZ238	*Coptodon zillii*	*Coptodon zillii*	MG407387	100	/	/	/
SZ239	*Sinibrama macrops*	*Sinibrama macrops*	MZ403625	100	*Sinibrama wui*	private	96.35
SZ240	*Sinibrama macrops*	*Sinibrama macrops*	MZ403625	100	*Sinibrama wui*	private	96.35
SZ241	*Pseudolaubuca sinensis*	*Pseudolaubuca sinensis*	MT805611	100	/	/	/
SZ242	*Hemiculter leucisculus*	*Hemiculter leucisculus*	MZ403353	100	/	/	/

SZ243	*Pseudohemiculter dispar*	*Pseudohemiculter dispar*	MZ403505	100	/	/	/
SZ244	*Zacco platypus*	*Zacco platypus*	MZ149250	100	*Zacco acutipinnis*	NC028595	93.64
SZ251	*Siniperca scherzeri*	*Siniperca scherzeri*	MZ149211	100	*Siniperca roulei*	KP710957	96.42
SZ252	*Siniperca scherzeri*	*Siniperca scherzeri*	MZ149211	100	*Siniperca roulei*	KP710957	96.42
SZ254	*Siniperca* sp.	*Siniperca knerii*	MT805838	100	*Siniperca chuatsi*	private	100
SZ255	*Siniperca scherzeri*	*Siniperca scherzeri*	MZ149211	100	*Siniperca roulei*	KP710957	96.42
SZ256	*Siniperca* sp.	*Siniperca knerii*	MT805833	100	*Siniperca chuatsi*	KX224166	99.84
SZ258	*Pseudohemiculter dispar*	*Pseudohemiculter dispar*	MZ403512	100	/	/	/
SZ259	*Pseudolaubuca engraulis*	*Pseudolaubuca engraulis*	MZ403565	100	/	/	/
SZ260	*Pseudorasbora parva*	*Pseudorasbora parva*	MT571919	100	/	/	/
SZ262	*Pseudolaubuca engraulis*	*Pseudolaubuca engraulis*	KR862091	100	/	/	/
SZ263	*Pseudohemiculter dispar*	*Pseudohemiculter dispar*	MZ403505	100	/	/	/
SZ264	*Pseudohemiculter dispar*	*Pseudohemiculter dispar*	MZ403515	100	/	/	/
SZ265	*Pseudohemiculter dispar*	*Pseudohemiculter dispar*	MZ403505	100	/	/	/
SZ266	*Siniperca* sp.	*Siniperca knerii*	MT805838	100	*Siniperca chuatsi*	private	100
SZ267	*Pseudolaubuca engraulis*	*Pseudolaubuca engraulis*	KR862091	100	/	/	/
SZ268	*Pseudohemiculter dispar*	*Pseudohemiculter dispar*	MZ403505	100	/	/	/
SZ269	*Hemiculter leucisculus*	*Hemiculter leucisculus*	MZ403354	100	/	/	/
SZ271	*Squalidus argentatus*	*Squalidus argentatus*	MT805839	100	/	/	/
SZ275	*Siniperca scherzeri*	*Siniperca scherzeri*	MZ149211	100	*Siniperca roulei*	KP710957	96.42
SZ276	*Siniperca* sp.	*Siniperca knerii*	MT805838	100	*Siniperca chuatsi*	private	100
SZ277	*Chanodichthys recurviceps*	*Chanodichthys recurviceps*	MZ403103	100	/	/	/
SZ278	*Pseudohemiculter dispar*	*Pseudohemiculter dispar*	MZ403505	100	/	/	/
SZ279	*Pseudohemiculter dispar*	*Pseudohemiculter dispar*	MZ403515	100	/	/	/
SZ280	*Mylopharyngodon piceus*	*Mylopharyngodon piceus*	KX224146	100	/	/	/
SZ281	*Squalidus argentatus*	*Squalidus argentatus*	MT805840	100	/	/	/
SZ282	*Rhinogobius* sp. 3	*Rhinogobius giurinus*	private	100	*Rhinogobius similis*	MT805715	100
SZ284	*Unknown sp.ecies* 1	*NA*	*NA*	*NA*	*NA*	*NA*	*NA*
SZ285	*Chanodichthys* sp.	*Chanodichthys alburnus*	MZ403235	100	*Chanodichthys erythropterus*	HQ536352	99.84

SZ286	*Squaliobarbus curriculus*	*Squaliobarbus curriculus*	MT805842	100	/	/	/
SZ288	*Hemiculter leucisculus*	*Hemiculter leucisculus*	MZ403353	99.5	/	/	/
SZ289	*Chanodichthys recurviceps*	*Chanodichthys recurviceps*	MZ403103	100	/	/	/
SZ290	*Chanodichthys recurviceps*	*Chanodichthys recurviceps*	MZ403103	100	/	/	/
SZ291	*Rhinogobius* sp. 4	*Rhinogobius sp. Ceheng*	private	98.26	*Rhinogobius sp. Liping*	private	91.79
SZ292	*Pseudohemiculter dispar*	*Pseudohemiculter dispar*	MZ403505	100	/	/	/
SZ293	*Zacco platypus*	*Zacco platypus*	MZ149250	100	*Zacco acutipinnis*	NC028595	93.64
SZ294	*Hemiculter leucisculus*	*Hemiculter leucisculus*	MZ403308	100	/	/	/
SZ295	*Hemiculter leucisculus*	*Hemiculter leucisculus*	MZ403353	100	/	/	/
SZ296	*Oreochromis* sp. 3	*Oreochromis aureus*	MT525047	100	*Oreochromis niloticus*	MG407412	100
SZ297	*Hemiculter leucisculus*	*Hemiculter leucisculus*	MZ403354	100	/	/	/
SZ298	*Pseudohemiculter dispar*	*Pseudohemiculter dispar*	MZ403505	100	/	/	/
SZ299	*Pseudohemiculter dispar*	*Pseudohemiculter dispar*	MZ403512	100	/	/	/
SZ313	*Hemiculterella sauvagei*	*Hemiculterella sauvagei*	MZ403272	100	*Pseudohemiculter dispar*	NC020435	97.83
SZ314	*Rhinogobius* sp.3	*Rhinogobius giurinus*	private	100	*Rhinogobius similis*	MT805715	100
SZ315	*Rhinogobius cliffordpopei*	*Rhinogobius cliffordpopei*	MT805714	100	*Rhinogobius leavelli*	AB988820	95.56
SZ316	*Rhinogobius* sp.3	*Rhinogobius giurinus*	private	100	*Rhinogobius similis*	MT805715	100
SZ318	*Rhinogobius* sp. 3	*Rhinogobius giurinus*	private	100	*Rhinogobius similis*	MT805715	100
SZ319	*Squaliobarbus curriculus*	*Squaliobarbus curriculus*	MT805842	100	/	/	/
SZ321	*Rhinogobius* sp. 4	*Rhinogobius sp. Ceheng*	private	98.26	*Rhinogobius sp. Liping*	private	91.79
SZ322	*Rhinogobius* sp. 4	*Rhinogobius sp. Ceheng*	private	98.26	*Rhinogobius sp. Liping*	private	91.79
SZ326	*Hemiculter leucisculus*	*Hemiculter leucisculus*	MZ403308	100	/	/	/
SZ327	*Pseudohemiculter dispar*	*Pseudohemiculter dispar*	MZ403505	100	/	/	/
SZ332	*Siniperca scherzeri*	*Siniperca scherzeri*	MZ149211	100	*Siniperca roulei*	KP710957	96.42
SZ333	*Siniperca scherzeri*	*Siniperca scherzeri*	MZ149211	100	*Siniperca roulei*	KP710957	96.42
SZ334	*Siniperca* sp.	*Siniperca knerii*	MT805838	100	*Siniperca chuatsi*	private	100
SZ335	*Rhinogobius* sp. 4	*Rhinogobius sp. Ceheng*	private	98.26	*Rhinogobius sp. Liping*	private	91.79
SZ336	*Siniperca scherzeri*	*Siniperca scherzeri*	MZ149211	100	*Siniperca roulei*	KP710957	96.42
SZ337	*Siniperca scherzeri*	*Siniperca scherzeri*	MZ149211	100	*Siniperca roulei*	KP710957	96.42

SZ338	*Squaliobarbus curriculus*	*Squaliobarbus curriculus*	MT805911	100	/	/	/
SZ339	*Squaliobarbus curriculus*	*Squaliobarbus curriculus*	MT805911	100	/	/	/
SZ340	*Squalidus argentatus*	*Squalidus argentatus*	MT805840	100	/	/	/
SZ341	*Squalidus argentatus*	*Squalidus argentatus*	MT805840	100	/	/	/
SZ342	*Pseudohemiculter dispar*	*Pseudohemiculter dispar*	MZ403515	100	/	/	/
SZ343	*Pseudolaubuca engraulis*	*Pseudolaubuca engraulis*	MZ403563	100	/	/	/
SZ344	*Pseudohemiculter dispar*	*Pseudohemiculter dispar*	MZ403505	100	/	/	/
SZ345	*Squaliobarbus curriculus*	*Squaliobarbus curriculus*	MT805842	100	/	/	/
SZ346	*Siniperca* sp.	*Siniperca knerii*	MT805838	100	*Siniperca chuatsi*	private	100
SZ347	*Toxabramis houdemeri*	*Toxabramis houdemeri*	MZ403721	100	*Pseudohemiculter dispar*	KF029684	94.34
SZ348	*Pseudolaubuca engraulis*	*Pseudolaubuca engraulis*	MZ403565	100	/	/	/
SZ350	*Pseudohemiculter dispar*	*Pseudohemiculter dispar*	MZ403505	100	/	/	/
SZ351	*Pseudohemiculter dispar*	*Pseudohemiculter dispar*	MZ403515	100	/	/	/
SZ352	*Siniperca* sp.	*Siniperca knerii*	MT805838	100	*Siniperca chuatsi*	private	100
SZ357	*Pseudolaubuca engraulis*	*Pseudolaubuca engraulis*	KR862091	100	/	/	/
SZ360	*Pseudolaubuca sinensis*	*Pseudolaubuca sinensis*	MZ403617	100	/	/	/
SZ362	*Rhinogobius* sp. 1	*Rhinogobius leavelli*	AB988820	99.68	*Rhinogobius maculafasciatus*	KU944927	99.09
SZ364	*Sinibrama macrops*	*Sinibrama macrops*	MZ403625	100	*Sinibrama wui*	private	96.35
SZ366	*Pseudohemiculter dispar*	*Pseudohemiculter dispar*	MZ403505	100	/	/	/
SZ367	*Schistura* sp.	*Schistura cf. fasciolata*	private	98.42	*Schistura cf. incerta*	private	97.47
SZ368	*Pseudohemiculter dispar*	*Pseudohemiculter dispar*	MZ403505	100	/	/	/
SZ371	*Sinibrama macrops*	*Sinibrama macrops*	MZ403625	100	*Sinibrama wui*	private	96.35
SZ372	*Squaliobarbus curriculus*	*Squaliobarbus curriculus*	MT805842	100	/	/	/
SZ373	*Sinibrama macrops*	*Sinibrama macrops*	MZ403625	100	*Sinibrama wui*	private	96.35
SZ374	*Siniperca scherzeri*	*Siniperca scherzeri*	MZ149211	100	*Siniperca roulei*	KP710957	96.42
SZ375	*Squaliobarbus curriculus*	*Squaliobarbus curriculus*	MT805842	100	/	/	/
SZ376	*Squaliobarbus curriculus*	*Squaliobarbus curriculus*	MT805842	100	/	/	/
SZ377	*Squaliobarbus curriculus*	*Squaliobarbus curriculus*	MT805842	100	/	/	/
SZ378	*Sinibrama melrosei*	*Sinibrama melrosei*	MZ403636	100	*Sinibrama macrops*	AP012112	95.5

SZ379	*Squaliobarbus curriculus*	*Squaliobarbus curriculus*	MW379547	100	/	/	/
SZ380	*Squaliobarbus curriculus*	*Squaliobarbus curriculus*	MT805842	100	/	/	/
SZ381	*Squaliobarbus curriculus*	*Squaliobarbus curriculus*	MT805842	100	/	/	/
SZ382	*Squaliobarbus curriculus*	*Squaliobarbus curriculus*	MT805842	100	/	/	/
SZ383	*Sinibotia robusta*	*Sinibotia robusta*	JN177235	100	/	/	/
SZ384	*Siniperca* sp.	*Siniperca knerii*	MT805838	100	*Siniperca chuatsi*	private	100
SZ385	*Squaliobarbus curriculus*	*Squaliobarbus curriculus*	MT805842	100	/	/	/
SZ386	*Siniperca* sp.	*Siniperca knerii*	MT805838	100	*Siniperca chuatsi*	private	100
SZ387	*Squaliobarbus curriculus*	*Squaliobarbus curriculus*	MT805842	100	/	/	/
SZ388	*Squaliobarbus curriculus*	*Squaliobarbus curriculus*	MT805842	100	/	/	/
SZ389	*Squaliobarbus curriculus*	*Squaliobarbus curriculus*	MT805842	100	/	/	/
SZ391	*Squaliobarbus curriculus*	*Squaliobarbus curriculus*	MT805843	100	/	/	/
SZ392	*Squaliobarbus curriculus*	*Squaliobarbus curriculus*	MT805842	100	/	/	/
SZ393	*Squaliobarbus curriculus*	*Squaliobarbus curriculus*	MT805846	100	/	/	/
SZ394	*Squaliobarbus curriculus*	*Squaliobarbus curriculus*	MT805842	100	/	/	/
SZ395	*Pseudohemiculter dispar*	*Pseudohemiculter dispar*	MZ403505	100	/	/	/
SZ396	*Squaliobarbus curriculus*	*Squaliobarbus curriculus*	MT805846	100	/	/	/
SZ397	*Squaliobarbus curriculus*	*Squaliobarbus curriculus*	MT805842	100	/	/	/
SZ398	*Squaliobarbus curriculus*	*Squaliobarbus curriculus*	MT805842	100	/	/	/
SZ399	*Squaliobarbus curriculus*	*Squaliobarbus curriculus*	MT805846	100	/	/	/
SZ400	*Squaliobarbus curriculus*	*Squaliobarbus curriculus*	MT805842	100	/	/	/
SZ401	*Squaliobarbus curriculus*	*Squaliobarbus curriculus*	MT805842	100	/	/	/
SZ402	*Pseudohemiculter dispar*	*Pseudohemiculter dispar*	private	99.84	/	/	/
SZ403	*Rhinogobius* sp. 4	*Rhinogobius sp. Ceheng*	private	98.26	*Rhinogobius sp. Liping*	private	91.79
SZ404	*Squaliobarbus curriculus*	*Squaliobarbus curriculus*	MT805842	100	/	/	/
SZ405	*Squaliobarbus curriculus*	*Squaliobarbus curriculus*	MT805842	100	/	/	/
SZ406	*Squaliobarbus curriculus*	*Squaliobarbus curriculus*	private	100	/	/	/
SZ407	*Squaliobarbus curriculus*	*Squaliobarbus curriculus*	MT805842	100	/	/	/
SZ408	*Pseudohemiculter dispar*	*Pseudohemiculter dispar*	MZ403505	100	/	/	/

SZ409	*Squaliobarbus curriculus*	*Squaliobarbus curriculus*	MT805875	100	/	/	/
SZ410	*Squaliobarbus curriculus*	*Squaliobarbus curriculus*	MT805911	100	/	/	/
SZ411	*Pseudohemiculter dispar*	*Pseudohemiculter dispar*	MZ403505	100	/	/	/
SZ412	*Squaliobarbus curriculus*	*Squaliobarbus curriculus*	MT805842	100	/	/	/
SZ413	*Hemiculterella wui*	*Hemiculterella wui*	private	99.84	*Pseudohemiculter dispar*	KF029684	94.97
SZ414	*Siniperca scherzeri*	*Siniperca scherzeri*	MZ149211	100	*Siniperca roulei*	KP710957	96.42
SZ415	*Squaliobarbus curriculus*	*Squaliobarbus curriculus*	MT805911	100	/	/	/
SZ416	*Pseudohemiculter dispar*	*Pseudohemiculter dispar*	MZ403536	100	/	/	/
SZ417	*Neosalanx* sp.	*Neosalanx tangkahkeii*	GQ848972	100	*Neosalanx brevirostris*	private	100
SZ418	*Pseudohemiculter dispar*	*Pseudohemiculter dispar*	MZ403505	100	/	/	/
SZ419	*Pseudolaubuca engraulis*	*Pseudolaubuca engraulis*	MZ403561	100	/	/	/
SZ420	*Chanodichthys recurviceps*	*Chanodichthys recurviceps*	MZ403103	100	/	/	/
SZ422	*Pseudohemiculter dispar*	*Pseudohemiculter dispar*	MZ403515	100	/	/	/
SZ423	*Pseudohemiculter dispar*	*Pseudohemiculter dispar*	MZ403505	100	/	/	/
SZ424	*Squaliobarbus curriculus*	*Squaliobarbus curriculus*	MT805842	100	/	/	/
SZ425	*Squaliobarbus curriculus*	*Squaliobarbus curriculus*	MT805843	100	/	/	/
SZ426	*Zacco platypus*	*Zacco platypus*	MZ149250	100	*Zacco acutipinnis*	NC028595	93.64
SZ427	*Pseudohemiculter dispar*	*Pseudohemiculter dispar*	MZ403505	100	/	/	/
SZ428	*Squaliobarbus curriculus*	*Squaliobarbus curriculus*	MT805842	100	/	/	/
SZ429	*Pseudohemiculter dispar*	*Pseudohemiculter dispar*	MZ403505	100	/	/	/
SZ430	*Pseudohemiculter dispar*	*Pseudohemiculter dispar*	MZ403505	100	/	/	/
SZ431	*Pseudohemiculter dispar*	*Pseudohemiculter dispar*	MZ403505	100	/	/	/
SZ433	*Squaliobarbus curriculus*	*Squaliobarbus curriculus*	MT805842	100	/	/	/
SZ434	*Squaliobarbus curriculus*	*Squaliobarbus curriculus*	MT805842	100	/	/	/
SZ435	*Sinibotia robusta*	*Sinibotia robusta*	MH027665	100	/	/	/
SZ436	*Squaliobarbus curriculus*	*Squaliobarbus curriculus*	MT805911	100	/	/	/
SZ437	*Squaliobarbus curriculus*	*Squaliobarbus curriculus*	MT805875	100	/	/	/
SZ438	*Sinibotia robusta*	*Sinibotia robusta*	MZ149177	100	/	/	/
SZ439	*Squaliobarbus curriculus*	*Squaliobarbus curriculus*	MT805842	100	/	/	/

SZ440	*Cirrhinus mrigala*	*Cirrhinus mrigala*	JX260850	100	/	/	/
SZ441	*Xenocypris* sp.	*Xenocypris davidi*	private	100	*Xenocypris macrolepis*	private	100
SZ442	*Squaliobarbus curriculus*	*Squaliobarbus curriculus*	MT805843	100	/	/	/
SZ443	*Sinibotia robusta*	*Sinibotia robusta*	AP011436	100	/	/	/
SZ444	*Cirrhinus mrigala*	*Cirrhinus mrigala*	JX260850	100	/	/	/
SZ445	*Squaliobarbus curriculus*	*Squaliobarbus curriculus*	MT805842	100	/	/	/
SZ446	*Squaliobarbus curriculus*	*Squaliobarbus curriculus*	MT805911	100	/	/	/
SZ447	*Squaliobarbus curriculus*	*Squaliobarbus curriculus*	MT805846	100	/	/	/
SZ448	*Squaliobarbus curriculus*	*Squaliobarbus curriculus*	MT805842	100	/	/	/
SZ449	*Cirrhinus mrigala*	*Cirrhinus mrigala*	JX260850	100	/	/	/
SZ450	*Squaliobarbus curriculus*	*Squaliobarbus curriculus*	MT805842	100	/	/	/
SZ451	*Squaliobarbus curriculus*	*Squaliobarbus curriculus*	MT805842	100	/	/	/
SZ452	*Squaliobarbus curriculus*	*Squaliobarbus curriculus*	MT805843	100	/	/	/
SZ453	*Cirrhinus mrigala*	*Cirrhinus mrigala*	JX260850	100	/	/	/
SZ454	*Squaliobarbus curriculus*	*Squaliobarbus curriculus*	MT805842	100	/	/	/
SZ455	*Squaliobarbus curriculus*	*Squaliobarbus curriculus*	MT805842	100	/	/	/
SZ456	*Squaliobarbus curriculus*	*Squaliobarbus curriculus*	MT805843	100	/	/	/
SZ457	*Squaliobarbus curriculus*	*Squaliobarbus curriculus*	MT805842	100	/	/	/
SZ458	*Squaliobarbus curriculus*	*Squaliobarbus curriculus*	MT805842	100	/	/	/
SZ459	*Squaliobarbus curriculus*	*Squaliobarbus curriculus*	MT805842	100	/	/	/
SZ460	*Cirrhinus mrigala*	*Cirrhinus mrigala*	JX260850	100	/	/	/
SZ461	*Cirrhinus mrigala*	*Cirrhinus mrigala*	JX260850	100	/	/	/
SZ462	*Squaliobarbus curriculus*	*Squaliobarbus curriculus*	MT805842	100	/	/	/
SZ463	*Cirrhinus mrigala*	*Cirrhinus mrigala*	JX260850	100	/	/	/
SZ464	*Squaliobarbus curriculus*	*Squaliobarbus curriculus*	MT805842	100	/	/	/
SZ465	*Squaliobarbus curriculus*	*Squaliobarbus curriculus*	MT805842	100	/	/	/
SZ466	*Hypophthalmichthys molitrix*	*Hypophthalmichthys molitrix*	FJ459502	100	/	/	/
SZ467	*Pseudohemiculter dispar*	*Pseudohemiculter dispar*	MZ403505	100	/	/	/
SZ468	*Cirrhinus mrigala*	*Cirrhinus mrigala*	JX260850	100	/	/	/

SZ469	*Squaliobarbus curriculus*	*Squaliobarbus curriculus*	MT805842	100	/	/	/
SZ470	*Siniperca* sp.	*Siniperca knerii*	MT805836	100	*Siniperca chuatsi*	MW402975	100
SZ471	*Sinibotia robusta*	*Sinibotia robusta*	MH027668	100	/	/	/
SZ472	*Rhinogobius* sp. 1	*Rhinogobius leavelli*	AB988820	100	*Rhinogobius maculafasciatus*	KU944927	99.46
SZ473	*Hypostomus* sp.	*Hypostomus sp.*	private	100	*Hypostomus plecostomus*	JN026849	100
SZ474	*Squalidus argentatus*	*Squalidus argentatus*	MT805840	100	/	/	/
SZ475	*Garra orientalis*	*Garra orientalis*	MZ148854	100	*Garra cyrano*	private	96.18
SZ476	*Mylopharyngodon piceus*	*Mylopharyngodon piceus*	MT805356	100	/	/	/
SZ477	*Squaliobarbus curriculus*	*Squaliobarbus curriculus*	MT805842	100	/	/	/
SZ478	*Squaliobarbus curriculus*	*Squaliobarbus curriculus*	MT805843	100	/	/	/
SZ479	*Pseudolaubuca sinensis*	*Pseudolaubuca sinensis*	MZ403617	100	/	/	/
SZ480	*Mylopharyngodon piceus*	*Mylopharyngodon piceus*	MT805356	100	/	/	/
SZ481	*Squaliobarbus curriculus*	*Squaliobarbus curriculus*	MT805842	100	/	/	/
SZ482	*Squaliobarbus curriculus*	*Squaliobarbus curriculus*	MT805842	100	/	/	/
SZ483	*Squaliobarbus curriculus*	*Squaliobarbus curriculus*	MT805842	100	/	/	/
SZ484	*Squaliobarbus curriculus*	*Squaliobarbus curriculus*	MT805842	100	/	/	/
SZ485	*Squaliobarbus curriculus*	*Squaliobarbus curriculus*	MT805842	100	/	/	/
SZ486	*Squaliobarbus curriculus*	*Squaliobarbus curriculus*	MT805842	100	/	/	/
SZ487	*Squaliobarbus curriculus*	*Squaliobarbus curriculus*	MT805842	100	/	/	/
SZ488	*Squaliobarbus curriculus*	*Squaliobarbus curriculus*	MT805843	100	/	/	/
SZ489	*Squaliobarbus curriculus*	*Squaliobarbus curriculus*	MT805842	100	/	/	/
SZ490	*Squaliobarbus curriculus*	*Squaliobarbus curriculus*	MT805843	100	/	/	/
SZ491	*Squaliobarbus curriculus*	*Squaliobarbus curriculus*	MT805842	100	/	/	/
SZ492	*Hypophthalmichthys molitrix*	*Hypophthalmichthys molitrix*	MK448068	100	/	/	/
SZ493	*Squaliobarbus curriculus*	*Squaliobarbus curriculus*	MT805879	100	/	/	/
SZ494	*Chanodichthys recurviceps*	*Chanodichthys recurviceps*	MZ403103	100	/	/	/
SZ495	*Squaliobarbus curriculus*	*Squaliobarbus curriculus*	MT805842	100	/	/	/
SZ496	*Coptodon zillii*	*Coptodon zillii*	MG407387	100	/	/	/
SZ497	*Rhinogobius* sp. 1	*Rhinogobius leavelli*	AB988820	99.68	*Rhinogobius maculafasciatus*	KU944927	99.09

SZ498	*Sinibotia robusta*	*Sinibotia robusta*	AP011436	100	/	/	/
SZ499	*Sinibotia robusta*	*Sinibotia robusta*	MZ149177	100	/	/	/
SZ500	*Cirrhinus mrigala*	*Cirrhinus mrigala*	JX260850	100	/	/	/
SZ501	*Acheilognathinae*	*Acheilognathus tonkinensis*	NC042407	99.69	*Rhodeus sericeus*	HQ557338	99.36
SZ502	*Sinibotia robusta*	*Sinibotia robusta*	KT374012	99.84	/	/	/
SZ503	*Oreochromis* sp. 1	*Oreochromis aureus*	MT525047	100	*Oreochromis niloticus*	private	100
SZ504	*Squaliobarbus curriculus*	*Squaliobarbus curriculus*	MT805842	100	/	/	/
SZ505	*Squaliobarbus curriculus*	*Squaliobarbus curriculus*	MT805842	100	/	/	/
SZ506	*Squaliobarbus curriculus*	*Squaliobarbus curriculus*	MT805842	100	/	/	/
SZ507	*Squaliobarbus curriculus*	*Squaliobarbus curriculus*	MT805842	100	/	/	/
SZ508	*Squaliobarbus curriculus*	*Squaliobarbus curriculus*	MT805842	100	/	/	/
SZ509	*Squaliobarbus curriculus*	*Squaliobarbus curriculus*	MT805843	100	/	/	/
SZ510	*Squaliobarbus curriculus*	*Squaliobarbus curriculus*	MT805842	100	/	/	/
SZ511	*Squaliobarbus curriculus*	*Squaliobarbus curriculus*	MT805842	100	/	/	/
SZ512	*Sinibotia robusta*	*Sinibotia robusta*	JN177235	100	/	/	/
SZ513	*Oreochromis* sp. 2	*Oreochromis niloticus*	MG407418	100	*Oreochromis placidus*	private	100
SZ514	*Pseudohemiculter dispar*	*Pseudohemiculter dispar*	MZ403505	100	/	/	/
SZ515	*Siniperca scherzeri*	*Siniperca scherzeri*	MZ149211	100	*Siniperca roulei*	KP710957	96.42
SZ516	*Siniperca scherzeri*	*Siniperca scherzeri*	MZ149211	100	*Siniperca roulei*	KP710957	96.42
SZ517	*Siniperca scherzeri*	*Siniperca scherzeri*	MZ149211	100	*Siniperca roulei*	KP710957	96.42
SZ518	*Sinibotia robusta*	*Sinibotia robusta*	JN177235	100	/	/	/
SZ519	*Squaliobarbus curriculus*	*Squaliobarbus curriculus*	MT805842	100	/	/	/
SZ520	*Pseudohemiculter dispar*	*Pseudohemiculter dispar*	MZ403505	100	/	/	/
SZ521	*Pseudohemiculter dispar*	*Pseudohemiculter dispar*	MZ403505	100	/	/	/
SZ522	*Siniperca* sp.	*Siniperca knerii*	MT805838	100	*Siniperca chuatsi*	private	100
SZ523	*Pseudohemiculter dispar*	*Pseudohemiculter dispar*	MZ403505	100	/	/	/
SZ524	*Chanodichthys recurviceps*	*Chanodichthys recurviceps*	MZ403074	100	/	/	/
SZ525	*Pseudohemiculter dispar*	*Pseudohemiculter dispar*	MZ403505	100	/	/	/
SZ526	*Squaliobarbus curriculus*	*Squaliobarbus curriculus*	MT805843	100	/	/	/

SZ527	*Squaliobarbus curriculus*	*Squaliobarbus curriculus*	MT805843	100	/	/	/
SZ528	*Pseudolaubuca engraulis*	*Pseudolaubuca engraulis*	MZ403565	100	/	/	/
SZ529	*Squaliobarbus curriculus*	*Squaliobarbus curriculus*	MT805851	100	/		
SZ530	*Siniperca* sp.	*Siniperca scherzeri*	MZ149211	100	*Siniperca roulei*	KP710957	96.42
SZ531	*Squaliobarbus curriculus*	*Squaliobarbus curriculus*	MT805842	100	/	/	/
SZ532	*Pseudohemiculter dispar*	*Pseudohemiculter dispar*	MZ403515	100	/	/	/
SZ533	*Squaliobarbus curriculus*	*Squaliobarbus curriculus*	MT805843	100	/	/	/
SZ534	*Chanodichthys recurviceps*	*Chanodichthys recurviceps*	MZ403103	100	/	/	/
SZ535	*Siniperca* sp.	*Siniperca knerii*	MT805838	100	*Siniperca chuatsi*	private	100
SZ536	*Squaliobarbus curriculus*	*Squaliobarbus curriculus*	MT805842	100	/	/	/
SZ537	*Chanodichthys recurviceps*	*Chanodichthys recurviceps*	MZ403103	100	/	/	/
SZ538	*Rhinogobius* sp. 4	*Rhinogobius sp. Ceheng*	private	98.26	*Rhinogobius sp. Liping*	private	91.79
SZ539	*Chanodichthys recurviceps*	*Chanodichthys recurviceps*	MZ403103	100	/	/	/
SZ540	*Chanodichthys recurviceps*	*Chanodichthys recurviceps*	MZ403103	100	/	/	/
SZ541	*Chanodichthys recurviceps*	*Chanodichthys recurviceps*	MZ403074	100	/	/	/
SZ542	*Squaliobarbus curriculus*	*Squaliobarbus curriculus*	MT805842	100	/	/	/
SZ543	*Siniperca* sp.	*Siniperca knerii*	MT805838	100	*Siniperca chuatsi*	private	100
SZ544	*Siniperca* sp.	*Siniperca knerii*	MT805838	100	*Siniperca chuatsi*	private	100
SZ545	*Siniperca* sp.	*Siniperca knerii*	MT805838	100	*Siniperca chuatsi*	private	100
SZ546	*Pseudohemiculter dispar*	*Pseudohemiculter dispar*	MZ403505	100	/	/	/
SZ547	*Squaliobarbus curriculus*	*Squaliobarbus curriculus*	MT805843	100	/	/	/
SZ548	*Chanodichthys* sp.	*Chanodichthys recurviceps*	private	100	*Chanodichthys erythropterus*	HQ536352	100
SZ549	*Chanodichthys recurviceps*	*Chanodichthys recurviceps*	MZ403103	100	/	/	/
SZ550	*Squalidus argentatus*	*Squalidus argentatus*	MZ149227	100	/	/	/
SZ551	*Hemiculter leucisculus*	*Hemiculter leucisculus*	MZ403354	100	/	/	/
SZ552	*Pseudohemiculter dispar*	*Pseudohemiculter dispar*	MZ403505	100	/	/	/
SZ553	*Chanodichthys recurviceps*	*Chanodichthys recurviceps*	MZ403103	100	/	/	/
SZ554	*Chanodichthys recurviceps*	*Chanodichthys recurviceps*	MZ403103	100	/	/	/
SZ555	*Squaliobarbus curriculus*	*Squaliobarbus curriculus*	MT805842	100	/	/	/

SZ557	*Squaliobarbus curriculus*	*Squaliobarbus curriculus*	MT805842	100	/	/	/
SZ558	*Siniperca* sp.	*Siniperca knerii*	MT805838	100	*Siniperca chuatsi*	private	100
SZ559	*Pseudohemiculter dispar*	*Pseudohemiculter dispar*	MZ403505	100	/	/	/
SZ560	*Squaliobarbus curriculus*	*Squaliobarbus curriculus*	MT805842	100	/	/	/
SZ561	*Pseudohemiculter dispar*	*Pseudohemiculter dispar*	MZ403505	100	/	/	/
SZ562	*Squalidus argentatus*	*Squalidus argentatus*	MT805840	100	/	/	/
SZ564	*Siniperca* sp.	*Siniperca knerii*	MT805836	100	*Siniperca chuatsi*	MW402975	100
SZ565	*Siniperca* sp.	*Siniperca knerii*	MT805838	100	*Siniperca chuatsi*	private	100
SZ566	*Pseudohemiculter dispar*	*Pseudohemiculter dispar*	MZ403505	100	/	/	/
SZ567	*Pseudohemiculter dispar*	*Pseudohemiculter dispar*	MZ403505	100	/	/	/
SZ568	*Siniperca* sp.	*Siniperca knerii*	MT805838	100	*Siniperca chuatsi*	private	100
SZ569	*Squaliobarbus curriculus*	*Squaliobarbus curriculus*	MT805842	100	/	/	/
SZ570	*Siniperca* sp.	*Siniperca knerii*	MT805838	100	*Siniperca chuatsi*	private	100
SZ571	*Chanodichthys recurviceps*	*Chanodichthys recurviceps*	MZ403103	100	/	/	/
SZ572	*Chanodichthys recurviceps*	*Chanodichthys recurviceps*	MZ403103	100	/	/	/
SZ573	*Pseudolaubuca engraulis*	*Pseudolaubuca engraulis*	MZ403563	100	/	/	/
SZ574	*Squaliobarbus curriculus*	*Squaliobarbus curriculus*	MT805842	100	/	/	/
SZ575	*Pseudohemiculter dispar*	*Pseudohemiculter dispar*	MZ403505	100	/	/	/
SZ576	*Pseudohemiculter dispar*	*Pseudohemiculter dispar*	MZ403505	100	/	/	/
SZ577	*Rhinogobius* sp. 4	*Rhinogobius sp. Ceheng*	private	98.26	*Rhinogobius sp. Liping*	private	91.79
SZ578	*Pseudohemiculter dispar*	*Pseudohemiculter dispar*	MZ403515	100	/	/	/
SZ579	*Pseudohemiculter dispar*	*Pseudohemiculter dispar*	MZ403505	100	/	/	/
SZ580	*Chanodichthys recurviceps*	*Chanodichthys recurviceps*	MZ403103	100	/	/	/
SZ581	*Sinibrama macrops*	*Sinibrama macrops*	MZ403625	100	*Sinibrama wui*	private	96.35
SZ582	*Chanodichthys recurviceps*	*Chanodichthys recurviceps*	MZ403103	100	/	/	/
SZ583	*Pseudohemiculter dispar*	*Pseudohemiculter dispar*	MZ403505	100	/	/	/
SZ584	*Pseudohemiculter dispar*	*Pseudohemiculter dispar*	MZ403505	100	/	/	/
SZ585	*Squaliobarbus curriculus*	*Squaliobarbus curriculus*	MT805842	100	/	/	/
SZ586	*Chanodichthys recurviceps*	*Chanodichthys recurviceps*	MZ403103	100	/	/	/

SZ587	*Chanodichthys recurviceps*	*Chanodichthys recurviceps*	MZ403103	100	/	/	/
SZ588	*Chanodichthys recurviceps*	*Chanodichthys recurviceps*	MZ403103	100	/	/	/
SZ589	*Pseudohemiculter dispar*	*Pseudohemiculter dispar*	MZ403505	100	/	/	/
SZ590	*Pseudohemiculter dispar*	*Pseudohemiculter dispar*	MZ403505	100	/	/	/
SZ591	*Squaliobarbus curriculus*	*Squaliobarbus curriculus*	MT805842	100	/	/	/
SZ592	*Squaliobarbus curriculus*	*Squaliobarbus curriculus*	MT805843	100	/	/	/
SZ593	*Squalidus argentatus*	*Squalidus argentatus*	MT805840	100	/	/	/
SZ594	*Pseudohemiculter dispar*	*Pseudohemiculter dispar*	MZ403505	100	/	/	/
SZ595	*Hyporhamphus intermedius*	*Hyporhamphus intermedius*	private	100	*Rhynchorhamphus georgii*	JQ738540	87.84
SZ597	*Chanodichthys recurviceps*	*Chanodichthys recurviceps*	MZ403103	100	/	/	/
SZ598	*Squaliobarbus curriculus*	*Squaliobarbus curriculus*	MT805842	100	/	/	/
SZ599	*Pseudohemiculter dispar*	*Pseudohemiculter dispar*	MZ403505	100	/	/	/
SZ600	*Pseudohemiculter dispar*	*Pseudohemiculter dispar*	MZ403505	100	/	/	/
SZ601	*Squaliobarbus curriculus*	*Squaliobarbus curriculus*	MT805842	100	/	/	/
SZ602	*Squaliobarbus curriculus*	*Squaliobarbus curriculus*	MT805842	100	/	/	/
SZ603	*Siniperca* sp.	*Siniperca knerii*	MT805838	100	*Siniperca chuatsi*	private	100
SZ604	*Chanodichthys recurviceps*	*Chanodichthys recurviceps*	MZ403103	100	/	/	/
SZ605	*Pseudohemiculter dispar*	*Pseudohemiculter dispar*	MZ403505	100	/	/	/
SZ606	*Siniperca scherzeri*	*Siniperca scherzeri*	MZ149211	100	*Siniperca roulei*	KP710957	96.42
SZ607	*Siniperca scherzeri*	*Siniperca scherzeri*	MZ149211	100	*Siniperca roulei*	KP710957	96.42
SZ608	*Siniperca* sp.	*Siniperca chuatsi*	KX224166	99.84	*Siniperca knerii*	EF143389	99.84
SZ609	*Chanodichthys recurviceps*	*Chanodichthys recurviceps*	MZ403103	100	/	/	/
SZ610	*Hypostomus* sp.	*Hypostomus sp.*	private	100	*Hypostomus plecostomus*	JN026849	100
SZ611	*Pseudolaubuca sinensis*	*Pseudolaubuca sinensis*	MZ403617	100	/	/	/
SZ612	*Sinibotia robusta*	*Sinibotia robusta*	MH027665	100	/	/	/
SZ613	*Sinibotia robusta*	*Sinibotia robusta*	JN177235	100	/	/	/
SZ614	*Sinibotia robusta*	*Sinibotia robusta*	private	99.84	/	/	/
SZ615	*Squaliobarbus curriculus*	*Squaliobarbus curriculus*	MT805843	100	/	/	/
SZ616	*Chanodichthys recurviceps*	*Chanodichthys recurviceps*	MZ403074	100	/	/	/

SZ617	*Sinibotia robusta*	*Sinibotia robusta*	MH027665	100	/	/	/
SZ618	*Mylopharyngodon piceus*	*Mylopharyngodon piceus*	KX224146	100	/	/	/
SZ619	*Sinibotia robusta*	*Sinibotia robusta*	JN177235	100	/	/	/
SZ620	*Sinibotia robusta*	*Sinibotia robusta*	JN177235	100	/	/	/
SZ621	*Squaliobarbus curriculus*	*Squaliobarbus curriculus*	MT805843	100	/	/	/
SZ622	*Sinibotia robusta*	*Sinibotia robusta*	JN177235	100	/	/	/
SZ623	*Rhinogobius* sp. 3	*Rhinogobius giurinus*	private	100	*Rhinogobius similis*	MT805720	100
SZ624	*Squaliobarbus curriculus*	*Squaliobarbus curriculus*	MT805842	100	/	/	/
SZ625	*Sinibotia robusta*	*Sinibotia robusta*	JN177235	100	/	/	/
SZ626	*Rhinogobius* sp. 3	*Rhinogobius giurinus*	private	100	*Rhinogobius similis*	MT805715	100
SZ627	*Sinibrama macrops*	*Sinibrama macrops*	MZ403625	100	*Sinibrama wui*	private	96.35
SZ628	*Sinibotia robusta*	*Sinibotia robusta*	MH027667	100	/	/	/
SZ629	*Sinibotia robusta*	*Sinibotia robusta*	MT805753	100	/	/	/
SZ630	*Pseudolaubuca sinensis*	*Pseudolaubuca sinensis*	MZ403617	100	/	/	/
SZ631	*Rhinogobius* sp. 3	*Rhinogobius giurinus*	private	100	*Rhinogobius similis*	MT805715	100
SZ632	*Sinibotia robusta*	*Sinibotia robusta*	MH027665	100	/	/	/
SZ633	*Chanodichthys recurviceps*	*Chanodichthys recurviceps*	MZ403103	100	/	/	/
SZ634	*Squaliobarbus curriculus*	*Squaliobarbus curriculus*	MT805842	100	/	/	/
SZ635	*Squaliobarbus curriculus*	*Squaliobarbus curriculus*	MT805843	100	/	/	/
SZ636	*Squaliobarbus curriculus*	*Squaliobarbus curriculus*	MT805843	100	/	/	/
SZ637	*Chanodichthys recurviceps*	*Chanodichthys recurviceps*	MZ403103	100	/	/	/
SZ638	*Squalidus argentatus*	*Squalidus argentatus*	MZ149246	100	*Squalidus chankaensis*	private	98.89
SZ639	*Squaliobarbus curriculus*	*Squaliobarbus curriculus*	MT805842	100	/	/	/
SZ641	*Sinibotia robusta*	*Sinibotia robusta*	MH027668	100	/	/	/
SZ642	*Sinibotia robusta*	*Sinibotia robusta*	KC871170	100	/	/	/
SZ643	*Sinibotia robusta*	*Sinibotia robusta*	JN177235	100	/	/	/
SZ644	*Sinibotia robusta*	*Sinibotia robusta*	JN177235	100	/	/	/
SZ645	*Sinibotia robusta*	*Sinibotia robusta*	MH027670	100	/	/	/
SZ646	*Squaliobarbus curriculus*	*Squaliobarbus curriculus*	MT805842	100	/	/	/

SZ647	*Sinibotia robusta*	*Sinibotia robusta*	MT805748	100	/	/	/
SZ648	*Squaliobarbus curriculus*	*Squaliobarbus curriculus*	MT805842	100	/	/	/
SZ649	*Sinibotia robusta*	*Sinibotia robusta*	JN177235	100	/	/	/
SZ650	*Sinibotia robusta*	*Sinibotia robusta*	JN177235	100	/	/	/
SZ651	*Squaliobarbus curriculus*	*Squaliobarbus curriculus*	MT805842	100	/	/	/
SZ652	*Sinibotia robusta*	*Sinibotia robusta*	MT805742	100	/	/	/
SZ653	*Sinibotia robusta*	*Sinibotia robusta*	MH027668	100	/	/	/
SZ654	*Siniperca* sp.	*Siniperca knerii*	MT805838	100	*Siniperca chuatsi*	private	100
SZ655	*Siniperca scherzeri*	*Siniperca scherzeri*	MZ149211	100	*Siniperca roulei*	KP710957	96.42
SZ657	*Sinibotia robusta*	*Sinibotia robusta*	MT805748	100	/	/	/
SZ658	*Siniperca* sp.	*Siniperca knerii*	MT805838	100	*Siniperca chuatsi*	private	100
SZ659	*Squaliobarbus curriculus*	*Squaliobarbus curriculus*	MT805843	100	/	/	/
SZ660	*Sinibotia robusta*	*Sinibotia robusta*	MT805748	100	/	/	/
SZ661	*Pseudohemiculter dispar*	*Pseudohemiculter dispar*	MZ403515	100	/	/	/
SZ662	*Sinibotia robusta*	*Sinibotia robusta*	MH027665	100	/	/	/
SZ663	*Pseudohemiculter dispar*	*Pseudohemiculter dispar*	MZ149111	100	/	/	/
SZ664	*Squaliobarbus curriculus*	*Squaliobarbus curriculus*	MT805842	100	/	/	/
SZ665	*Sinibotia robusta*	*Sinibotia robusta*	MZ149177	100	/	/	/
SZ666	*Squaliobarbus curriculus*	*Squaliobarbus curriculus*	MT805842	100	/	/	/
SZ667	*Squaliobarbus curriculus*	*Squaliobarbus curriculus*	MT805906	100	/	/	/
SZ668	*Sinibotia robusta*	*Sinibotia robusta*	JN177235	100	/	/	/
SZ669	*Sinibotia robusta*	*Sinibotia robusta*	KT374012	100	/	/	/
SZ670	*Rhinogobius* sp. 3	Rhinogobius similis	MT571961	99.84	*Rhinogobius giurinus*	private	99.68
SZ671	*Siniperca scherzeri*	*Siniperca scherzeri*	MZ149211	100	*Siniperca roulei*	KP710957	96.42
SZ672	*Sinibotia robusta*	*Sinibotia robusta*	MZ149177	100	/	/	/
SZ673	*Squaliobarbus curriculus*	*Squaliobarbus curriculus*	MT805842	100	/	/	/
SZ674	*Siniperca* sp.	*Siniperca knerii*	MT805833	100	*Siniperca chuatsi*	KX224166	99.84
SZ675	*Pseudolaubuca sinensis*	*Pseudolaubuca sinensis*	MZ403617	100	/	/	/
SZ676	*Siniperca scherzeri*	*Siniperca scherzeri*	MZ149211	100	*Siniperca roulei*	KP710957	96.42

SZ677	*Pseudolaubuca sinensis*	*Pseudolaubuca sinensis*	MZ403617	100	/	/	/
SZ678	*Siniperca scherzeri*	*Siniperca scherzeri*	MZ149211	100	*Siniperca roulei*	KP710957	96.42
SZ679	*Squalidus argentatus*	*Squalidus argentatus*	MT805840	100	/	/	/
SZ680	*Chanodichthys recurviceps*	*Chanodichthys recurviceps*	MZ403103	100	/	/	/
SZ681	*Siniperca* sp.	*Siniperca knerii*	MT805838	100	*Siniperca chuatsi*	private	100
SZ682	*Chanodichthys recurviceps*	*Chanodichthys recurviceps*	MZ403103	100	/	/	/
SZ683	*Sinibrama macrops*	*Sinibrama macrops*	MZ403625	100	*Sinibrama wui*	private	96.35
SZ684	*Chanodichthys recurviceps*	*Chanodichthys recurviceps*	MZ403103	100	/	/	/
SZ685	*Squaliobarbus curriculus*	*Squaliobarbus curriculus*	private	99.83	/	/	/
SZ686	*Siniperca* sp.	*Siniperca knerii*	MT805838	100	*Siniperca chuatsi*	private	100
SZ687	*Siniperca* sp.	*Siniperca knerii*	MT805838	100	*Siniperca chuatsi*	private	100
SZ688	*Siniperca* sp.	*Siniperca knerii*	MT805838	100	*Siniperca chuatsi*	private	100
SZ689	*Oreochromis* sp. 2	*Oreochromis niloticus*	MG407418	100	*Oreochromis placidus*	private	100
SZ690	*Siniperca scherzeri*	*Siniperca scherzeri*	MZ149211	100	*Siniperca roulei*	KP710957	96.42
SZ691	*Squalidus argentatus*	*Squalidus argentatus*	MZ149246	100	*Squalidus chankaensis*	private	98.89
SZ692	*Mugilogobius myxodermus*	*Mugilogobius myxodermus*	MT805276	100	*Mugilogobius abei*	KF128984	90.85
SZ693	*Squaliobarbus curriculus*	*Squaliobarbus curriculus*	MT805842	100	/	/	/
SZ694	*Pseudohemiculter dispar*	*Pseudohemiculter dispar*	MZ403505	100	/	/	/
SZ695	*Pseudohemiculter dispar*	*Pseudohemiculter dispar*	MZ403505	100	/	/	/
SZ696	*Squaliobarbus curriculus*	*Squaliobarbus curriculus*	MT805842	100	/	/	/
SZ697	*Siniperca* sp.	*Siniperca knerii*	MT805833	100	*Siniperca chuatsi*	KX224166	99.84
SZ698	*Squaliobarbus curriculus*	*Squaliobarbus curriculus*	MT805843	100	/	/	/
SZ699	*Squaliobarbus curriculus*	*Squaliobarbus curriculus*	MT805842	100	/	/	/
SZ700	*Squaliobarbus curriculus*	*Squaliobarbus curriculus*	MT805842	100	/	/	/
SZ701	*Squaliobarbus curriculus*	*Squaliobarbus curriculus*	MW379547	100	/	/	/
SZ702	*Squaliobarbus curriculus*	*Squaliobarbus curriculus*	MT805911	100	/	/	/
SZ703	*Siniperca* sp.	*Siniperca knerii*	MT805838	100	*Siniperca chuatsi*	private	100
SZ704	*Squaliobarbus curriculus*	*Squaliobarbus curriculus*	MT805842	100	/	/	/
SZ705	*Pseudohemiculter dispar*	*Pseudohemiculter dispar*	MZ403505	100	/	/	/

SZ706	*Squaliobarbus curriculus*	*Squaliobarbus curriculus*	MT805842	100	/	/	/
SZ707	*Squaliobarbus curriculus*	*Squaliobarbus curriculus*	MW379547	100	/	/	/
SZ708	*Pseudolaubuca sinensis*	*Pseudolaubuca sinensis*	MZ403617	100	/	/	/
SZ709	*Squaliobarbus curriculus*	*Squaliobarbus curriculus*	MT805843	100	/	/	/
SZ710	*Pseudohemiculter dispar*	*Pseudohemiculter dispar*	MZ403505	100	/	/	/
SZ711	*Squaliobarbus curriculus*	*Squaliobarbus curriculus*	MT805842	100	/	/	/
SZ712	*Siniperca* sp.	*Siniperca knerii*	MT805838	100	*Siniperca chuatsi*	private	100
SZ713	*Chanodichthys recurviceps*	*Chanodichthys recurviceps*	MZ403103	100	/	/	/
SZ714	*Squaliobarbus curriculus*	*Squaliobarbus curriculus*	MT805842	100	/	/	/
SZ715	*Squaliobarbus curriculus*	*Squaliobarbus curriculus*	MT805842	100	/	/	/
SZ716	*Squaliobarbus curriculus*	*Squaliobarbus curriculus*	MT805843	100	/	/	/
SZ717	*Squaliobarbus curriculus*	*Squaliobarbus curriculus*	MT805842	100	/	/	/
SZ718	*Squaliobarbus curriculus*	*Squaliobarbus curriculus*	MT805842	100	/	/	/
SZ719	*Squaliobarbus curriculus*	*Squaliobarbus curriculus*	MT805843	100	/	/	/
SZ720	*Squaliobarbus curriculus*	*Squaliobarbus curriculus*	MT805843	100	/	/	/
SZ721	*Squaliobarbus curriculus*	*Squaliobarbus curriculus*	MT805843	100	/	/	/
SZ722	*Pseudohemiculter dispar*	*Pseudohemiculter dispar*	MZ403512	100	/	/	/
SZ723	*Pseudohemiculter dispar*	*Pseudohemiculter dispar*	MZ403505	100	/	/	/
SZ724	*Rhinogobius* sp. 4	*Rhinogobius sp. Ceheng*	private	98.26	*Rhinogobius sp. Liping*	private	91.79
SZ725	*Squaliobarbus curriculus*	*Squaliobarbus curriculus*	MT805842	100	/	/	/
SZ726	*Squaliobarbus curriculus*	*Squaliobarbus curriculus*	MT805842	100	/	/	/
SZ727	*Pseudolaubuca engraulis*	*Pseudolaubuca engraulis*	MZ403563	100	/	/	/
SZ728	*Pseudohemiculter dispar*	*Pseudohemiculter dispar*	MZ403505	100	/	/	/
SZ729	*Squaliobarbus curriculus*	*Squaliobarbus curriculus*	MT805842	100	/	/	/
SZ1214	*Pseudolaubuca engraulis*	*Pseudolaubuca engraulis*	NC020462	99.84	/	/	/
SZ1773	*Hemiculter leucisculus*	*Hemiculter leucisculus*	MZ403354	100	/	/	/
SZ1965	*Pseudohemiculter dispar*	*Pseudohemiculter dispar*	MZ403512	100	/	/	/
SZ1992	*Siniperca* sp.	*Siniperca knerii*	MT805838	100	*Siniperca chuatsi*	private	100
SZ2011	*Neosalanx* sp.	*Neosalanx tangkahkeii*	GQ848972	100	*Neosalanx brevirostris*	private	100

SZ2027	*Pseudohemiculter dispar*	*Pseudohemiculter dispar*	MZ403505	100	/	/	/
SZ2032	*Squaliobarbus curriculus*	*Squaliobarbus curriculus*	MZ149249	100	/	/	/
SZ2038	*Neosalanx* sp.	*Neosalanx tangkahkeii*	GQ848972	100	*Neosalanx brevirostris*	private	100
SZ2668	*Siniperca scherzeri*	*Siniperca scherzeri*	MZ149211	100	*Siniperca roulei*	KP710957	96.42
SZ2675	*Siniperca* sp.	*Siniperca knerii*	MT805838	100	*Siniperca chuatsi*	private	100
SZ2676	*Siniperca scherzeri*	*Siniperca scherzeri*	MZ149211	100	*Siniperca roulei*	KP710957	96.42
SZ2686	*Siniperca* sp.	*Siniperca knerii*	MT805838	100	*Siniperca chuatsi*	private	100
SZ2687	*Siniperca* sp.	*Siniperca knerii*	MT805833	100	*Siniperca chuatsi*	KX224166	99.84
SZ2689	*Siniperca scherzeri*	*Siniperca scherzeri*	MZ149211	100	*Siniperca roulei*	KP710957	96.42
SZ2690	*Siniperca scherzeri*	*Siniperca scherzeri*	MZ149211	100	*Siniperca roulei*	KP710957	96.42
SZ2695	*Siniperca* sp.	*Siniperca knerii*	MT805836	100	*Siniperca chuatsi*	MW402975	100
SZ2697	*Siniperca scherzeri*	*Siniperca scherzeri*	MZ149211	100	*Siniperca roulei*	KP710957	96.42
SZ2698	*Siniperca scherzeri*	*Siniperca scherzeri*	MZ149211	100	*Siniperca roulei*	KP710957	96.42
SZ2701	*Siniperca* sp.	*Siniperca knerii*	MT805838	100	*Siniperca chuatsi*	private	100
SZ2714	*Chanodichthys* sp.	*Chanodichthys recurviceps*	private	100	*Chanodichthys erythropterus*	HQ536352	100
SZ2718	*Squaliobarbus curriculus*	*Squaliobarbus curriculus*	MT805842	100	/	/	/
SZ2721	*Squaliobarbus curriculus*	*Squaliobarbus curriculus*	MT805842	100	/	/	/
SZ2732	*Pseudohemiculter dispar*	*Pseudohemiculter dispar*	MZ403505	100	/	/	/
SZ2736	*Rhinogobius* sp. 4	*Rhinogobius sp. Ceheng*	private	98.26	*Rhinogobius sp. Liping*	private	91.79
SZ2738	*Siniperca* sp.	*Siniperca knerii*	MT805838	100	*Siniperca chuatsi*	private	100
SZ2740	*Siniperca scherzeri*	*Siniperca scherzeri*	MZ149211	100	*Siniperca roulei*	KP710957	96.42
SZ2741	*Siniperca* sp.	*Siniperca knerii*	MT805838	100	*Siniperca chuatsi*	private	100
SZ2742	*Squaliobarbus curriculus*	*Squaliobarbus curriculus*	MT805842	100	/	/	/
SZ2750	*Siniperca* sp.	*Siniperca knerii*	MT805838	100	*Siniperca chuatsi*	private	100
SZ2755	*Schistura* sp.	*Schistura cf. fasciolata*	private	98.42	*Schistura cf. incerta*	private	97.31
SZ2756	*Hemiculterella sauvagei*	*Hemiculterella sauvagei*	MZ403272	100	*Pseudohemiculter dispar*	NC020435	97.83
SZ2758	*Neosalanx* sp.	*Neosalanx tangkahkeii*	GQ848972	100	*Neosalanx brevirostris*	private	100
SZ2760	*Siniperca* sp.	*Siniperca knerii*	MT805838	100	*Siniperca chuatsi*	private	100
SZ2761	*Rhinogobius* sp. 4	*Rhinogobius sp. Ceheng*	private	98.26	*Rhinogobius sp. Liping*	private	91.79

SZ2762	*Squaliobarbus curriculus*	*Squaliobarbus curriculus*	MT805842	100	/	/	/
SZ2767	*Siniperca scherzeri*	*Siniperca scherzeri*	MZ149211	100	*Siniperca roulei*	KP710957	96.42
SZ2769	*Siniperca* sp.	*Siniperca knerii*	MT805838	100	*Siniperca chuatsi*	private	100
SZ2792	*AncherythroChanodichthys lini*	*AncherythroChanodichthys lini*	MZ402754	100	*Chanodichthys erythropterus*	MZ402850	96.17
SZ2793	*Squaliobarbus curriculus*	*Squaliobarbus curriculus*	MT805842	100	/	/	/
SZ2795	*Pseudohemiculter dispar*	*Pseudohemiculter dispar*	MZ403515	100	/	/	/
SZ2796	*Squaliobarbus curriculus*	*Squaliobarbus curriculus*	MT805842	100	/	/	/
SZ2798	*Squaliobarbus curriculus*	*Squaliobarbus curriculus*	MT805842	100	/	/	/
SZ2801	*AncherythroChanodichthys lini*	*AncherythroChanodichthys lini*	MZ402771	100	*Chanodichthys erythropterus*	MZ402850	96.33
SZ2802	*Chanodichthys recurviceps*	*Chanodichthys recurviceps*	MZ403103	100	/	/	/
SZ2803	*Pseudohemiculter dispar*	*Pseudohemiculter dispar*	MZ403505	100	/	/	/
SZ2804	*Rhinogobius* sp. 4	*Rhinogobius sp. Ceheng*	private	98.26	*Rhinogobius sp. Liping*	private	91.79
SZ2805	*Squaliobarbus curriculus*	*Squaliobarbus curriculus*	MT805950	100	/	/	/
SZ2806	*Squaliobarbus curriculus*	*Squaliobarbus curriculus*	MT805842	100	/	/	/
SZ2807	*Squaliobarbus curriculus*	*Squaliobarbus curriculus*	MT805842	100	/	/	/
SZ2812	*Siniperca* sp.	*Siniperca knerii*	MT805838	100	*Siniperca chuatsi*	private	100
SZ2813	*Chanodichthys recurviceps*	*Chanodichthys recurviceps*	MZ403103	100	/	/	/
SZ2814	*Chanodichthys recurviceps*	*Chanodichthys recurviceps*	MZ403103	100	/	/	/
SZ2815	*Pseudohemiculter dispar*	*Pseudohemiculter dispar*	MZ403505	100	/	/	/
SZ2822	*Mugilogobius myxodermus*	*Mugilogobius myxodermus*	MT805276	100	*Mugilogobius abei*	KF128984	90.85
SZ2841	*Siniperca* sp.	*Siniperca knerii*	MT805838	100	*Siniperca chuatsi*	private	100
SZ2843	*Neosalanx* sp.	*Neosalanx tangkahkeii*	GQ848972	100	*Neosalanx brevirostris*	private	100
SZ2857	*Siniperca* sp.	*Siniperca knerii*	MT805838	100	*Siniperca chuatsi*	private	100
SZ2867	*Sinibotia robusta*	*Sinibotia robusta*	JN177235	100	/	/	/
SZ2871	*Sinibotia robusta*	*Sinibotia robusta*	MH027668	100	/	/	/
SZ2874	*Sinibotia robusta*	*Sinibotia robusta*	MT805753	100	/	/	/
SZ2882	*Rhinogobius* sp.4	*Rhinogobius sp. Ceheng*	private	98.58	*Rhinogobius sp. Liping*	private	91.31
SZ2883	*Rhinogobius* sp.4	*Rhinogobius sp. Ceheng*	private	98.58	*Rhinogobius sp. Liping*	private	91.31

SZ2886	*Sinibotia robusta*	*Sinibotia robusta*	JN177235	100	/	/	/
SZ2943	*Siniperca* sp.	*Siniperca knerii*	MT805833	100	*Siniperca chuatsi*	KX224166	99.84
SZ2944	*Squaliobarbus curriculus*	*Squaliobarbus curriculus*	MT805843	100	/	/	/
SZ2949	*Squaliobarbus curriculus*	*Squaliobarbus curriculus*	MT805842	100	/	/	/
SZ2953	*Siniperca* sp.	*Siniperca knerii*	MT805838	100	*Siniperca chuatsi*	private	100
SZ2954	*Squaliobarbus curriculus*	*Squaliobarbus curriculus*	MT805911	100	/	/	/
SZ2955	*Squaliobarbus curriculus*	*Squaliobarbus curriculus*	MT805842	100	/	/	/
SZ2956	*Squaliobarbus curriculus*	*Squaliobarbus curriculus*	MT805843	100	/	/	/
SZ2957	*Mugilogobius myxodermus*	*Mugilogobius myxodermus*	MT805275	100	*Mugilogobius abei*	KF128984	91.01
SZ2959	*Pseudolaubuca sinensis*	*Pseudolaubuca sinensis*	MZ403617	100	/	/	/
SZ2960	*Pseudohemiculter dispar*	*Pseudohemiculter dispar*	MZ403505	100	/	/	/
SZ2963	*Squaliobarbus curriculus*	*Squaliobarbus curriculus*	MT805846	100	/	/	/
SZ2964	*Squaliobarbus curriculus*	*Squaliobarbus curriculus*	MT805851	100	/	/	/
SZ2965	*Sinibotia robusta*	*Sinibotia robusta*	MH027668	100	/	/	/
SZ2966	*Squaliobarbus curriculus*	*Squaliobarbus curriculus*	MT805842	100	/	/	/
SZ2967	*Siniperca* sp.	*Siniperca chuatsi*	KX224166	99.84	*Siniperca knerii*	EF143389	99.84
SZ2968	*Siniperca* sp.	*Siniperca knerii*	MT805838	100	*Siniperca chuatsi*	private	100
SZ2969	*Squaliobarbus curriculus*	*Squaliobarbus curriculus*	MT805842	100	/	/	/
SZ2971	*Squaliobarbus curriculus*	*Squaliobarbus curriculus*	MT805842	100	/	/	/
SZ2972	*Siniperca* sp.	*Siniperca knerii*	MT805838	100	*Siniperca chuatsi*	private	100
SZ2974	*Siniperca scherzeri*	*Siniperca scherzeri*	MZ149211	100	*Siniperca roulei*	KP710957	96.42
SZ2976	*Squaliobarbus curriculus*	*Squaliobarbus curriculus*	MT805842	100	/	/	/
SZ2978	*Squaliobarbus curriculus*	*Squaliobarbus curriculus*	MT805842	100	/	/	/
SZ2979	*Siniperca* sp.	*Siniperca knerii*	MT805838	100	*Siniperca chuatsi*	private	100
SZ2980	*Squaliobarbus curriculus*	*Squaliobarbus curriculus*	MT805842	100	/	/	/
SZ2984	*Siniperca* sp.	*Siniperca knerii*	MT805838	100	*Siniperca chuatsi*	private	100
