# Supplementary material for: Unraveling the Drifting Larval Fish Community in a Large Spawning Ground in the Middle Pearl River Using DNA Barcoding
Source: Animals (Basel). 2022 Sep 24;12(19):2555. doi: 10.3390/ani12192555 (PMC9559676; doi:10.3390/ani12192555)
Supplement: Supplementary file 1 [file animals-12-02555-s001.zip › Table S3.pdf]

Sample ID	Final name	Best match species	Genbank Nos	Identity	Nearest species	Genbank Nos	Identity
SZ189	*Rhinogobius* sp. 5	*Rhinogobius duospilus*	MH127918	96.14	*Rhinogobius virgigena*	AB988821	96.39
SZ284	*Rhinogobius* sp. 5	*Rhinogobius duospilus*	MH127918	96.14	*Rhinogobius virgigena*	AB988821	96.39
SZ177	*Rhinogobius* sp. 6	*Rhinogobius duospilus*	MH127918	97.84	*Rhinogobius virgigena*	AB988821	96.86
