# Supplementary material for: Unraveling the Drifting Larval Fish Community in a Large Spawning Ground in the Middle Pearl River Using DNA Barcoding
Source: Animals (Basel). 2022 Sep 24;12(19):2555. doi: 10.3390/ani12192555 (PMC9559676; doi:10.3390/ani12192555)
Supplement: Supplementary file 1 [file animals-12-02555-s001.zip › Table S4.pdf]

>Acheilognathinae_SZ185
GACATTGGCACCCTTTATCTCGTATTTGGTGCCTGAGCCGGAATAGTTGGAACCGCCCT
AAGCCTCCTCATTCGTGCCGAGCTAAGTCAGCCCGGCTCCCTCCTAGGCGACGACCAG
ATTTATAATGTAATCGTAACCGCCCACGCTTTCGTAATAATTTTCTTTATAGTAATGCCCA
TCCTCATCGGGGGATTCGGCAACTGGCTTGTACCGCTAATGATTGGAGCACCTGATATA
GCTTTCCCACGAATAAATAACATAAGCTTTTGACTTCTTCCTCCATCCTTCCTCCTGCTT
CTTGCCTCCTCAGGGGTTGAAGCAGGGGCCGGAACTGGCTGAACGGTGTACCCACCC
CTCGCTGGTAATTTAGCCCACGCAGGAGCCTCAGTAGACCTAACTATCTTCTCACTTCA
TTTAGCAGGGGCATCCTCAATTTTAGGTGCTATTAATTTTATTACTACAACCATTAATATG
AAGCCGCCTGCCATCTCCCAGTATCAAACACCTTTATTCGTGTGGGCTGTACTTGTAAC
AGCAGTACTTCTCCTATTGTCCCTCCCCGTTCTGGCCGCTGGAATCACAATGCTTCTTAC
GGACCGTAATTTAAATACTACATTCTTTGACCCGGCAGGAGGGGGAGACCCAAT
>Acheilognathinae_SZ501
GACATTGGCACCCTTTATCTCGTATTTGGTGCCTGAGCCGGAATAGTTGGAACCGCCCT
AAGCCTCCTCATTCGTGCCGAGCTAAGTCAGCCCGGCTCCCTCCTAGGCGACGACCAG
ATTTATAATGTAATCGTAACCGCCCACGCTTTCGTAATAATTTTCTTTATAGTAATGCCCA
TCCTCATCGGGGGATTCGGCAACTGGCTTGTACCGCTAATGATTGGAGCACCTGATATA
GCTTTCCCACGAATAAATAACATAAGCTTTTGACTTCTTCCTCCATCCTTCCTCTTGCTT
CTTGCCTCCTCAGGGGTTGAAGCAGGGGCCGGAACTGGCTGAACGGTGTACCCACCC
CTCGCTGGTAATTTAGCCCACGCAGGAGCCTCAGTAGACCTAACTATCTTCTCACTTCA
TTTAGCAGGGGCATCCTCAATTTTAGGTGCTATTAATTTTATTACTACAACCATTAATATG
AAGCCGCCTGCCATCTCCCAGTATCAAACACCTTTATTCGTGTGGGCTGTACTTGTAAC
AGCAGTACTTCTCCTATTGTCCCTCCCCGTTCTGGCCGCTGGAATCACAATGCTTCTTAC
GGACCGTAATTTAAATACTACATTCTTTGACCCGGCAGGAGGGGGAGACCCAAT
>Chanodichthys_sp_SZ2714
GACATTGGCACCCTTTATCTTGTATTTGGTGCCTGAGCCGGAATAGTGGGAACCGCTCT
AAGCCTTCTCATTCGAGCCGAACTAAGCCAACCTGGATCACTTCTGGGTGACGATCAA
ATTTATAATGTCATTGTTACTGCCCATGCCTTCGTAATAATTTTCTTTATAGTAATACCAAT
TCTAATCGGAGGGTTTGGAAACTGACTCGTGCCGCTAATAATCGGGGCACCTGATATAG
CATTCCCACGAATAAATAACATGAGTTTTTGACTTCTACCCCCCTCTTTCCTTCTGCTGC
TAGCCTCCTCTGGTGTCGAGGCCGGAGCTGGAACAGGGTGAACAGTATACCCGCCACT
CGCAGGCAATCTTGCCCACGCGGGAGCATCCGTAGACCTAACAATTTTCTCGCTTCACT
TAGCAGGTGTATCATCAATTTTAGGTGCAATTAACTTCATCACCACAACTATTAACATGA
AACCACCAGCCATTTCTCAATACCAAACACCCCTATTTGTCTGAGCTGTACTTGTAACA
GCCGTACTTCTTCTCCTATCCCTGCCAGTCCTAGCCGCTGGGATTACTATACTCCTTACA
GACCGAAATCTAAATACCACATTCTTTGACCCGGCAGGAGGAGGAGACCCGAT
>Chanodichthys_sp_SZ285
GACATTGGCACCCTTTATCTTGTATTTGGTGCCTGAGCCGGAATAGTGGGAACCGCTCT
AAGCCTTCTCATTCGAGCCGAACTAAGCCAACCTGGATCACTTCTGGGTGACGATCAA
ATTTATAATGTCATTGTTACTGCCCATGCCTTCGTAATAATTTTCTTTATAGTAATACCAAT
TCTAATCGGGGGGTTTGGAAACTGACTCGTGCCGCTAATAATCGGGGCACCTGATATAG
CATTCCCACGAATAAATAACATGAGTTTTTGACTTCTACCCCCCTCTTTCCTTCTGCTGC
TAGCCTCCTCTGGTGTCGAGGCCGGAGCTGGAACAGGGTGAACAGTATACCCGCCACT
CGCAGGCAATCTTGCCCACGCGGGAGCATCCGTAGACCTAACAATTTTCTCGCTTCACT

TAGCAGGTGTATCATCAATTTTAGGTGCAATTAACTTCATCACCACAACTATTAACATGA
AACCACCAGCCATTTCTCAATACCAAACACCCCTATTTGTCTGAGCTGTACTTGTAACA
GCCGTACTTCTTCTCCTATCCCTGCCAGTCCTAGCCGCTGGGATTACTATACTCCTTACA
GACCGAAATCTAAATACCACATTCTTTGACCCGGCAGGAGGAGGAGACCCGAT
>Chanodichthys_sp_SZ548
GACATTGGCACCCTTTATCTTGTATTTGGTGCCTGAGCCGGAATAGTGGGAACCGCTCT
AAGCCTTCTCATTCGAGCCGAACTAAGCCAACCTGGATCACTTCTGGGTGACGATCAA
ATTTATAATGTCATTGTTACTGCCCATGCCTTCGTAATAATTTTCTTTATAGTAATACCAAT
TCTAATCGGAGGGTTTGGAAACTGACTCGTGCCGCTAATAATCGGGGCACCTGATATAG
CATTCCCACGAATAAATAACATGAGTTTTTGACTTCTACCCCCCTCTTTCCTTCTGCTGC
TAGCCTCCTCTGGTGTCGAGGCCGGAGCTGGAACAGGGTGAACAGTATACCCGCCACT
CGCAGGCAATCTTGCCCACGCGGGAGCATCCGTAGACCTAACAATTTTCTCGCTTCACT
TAGCAGGTGTATCATCAATTTTAGGTGCAATTAACTTCATCACCACAACTATTAACATGA
AACCACCAGCCATTTCTCAATACCAAACACCCCTATTTGTCTGAGCTGTACTTGTAACA
GCCGTACTTCTTCTCCTATCCCTGCCAGTCCTAGCCGCTGGGATTACTATACTCCTTACA
GACCGAAATCTAAATACCACATTCTTTGACCCGGCAGGAGGAGGAGACCCGAT
>Hypostomus_sp_SZ473
GACATTGGCACCCTTTACTTAGTATTTGGTGCCTGAGCCGGAATGGTTGGCACAGCTCT
CAGCCTTTTAATTCGGGCTGAGCTAAGCCAACCCGGCTCCCTACTAGGTGATGACCAAA
TTTATAATGTCATCGTTACTGCACATGCCTTCGTAATAATTTTCTTTATAGTAATACCAATT
ATAATTGGAGGCTTCGGAAATTGACTAGTCCCACTAATGATTGGAGCACCCGACATAGC
CTTCCCACGAATAAATAATATGAGCTTCTGACTACTCCCCCCATCATTCCTTCTCCTATTA
GCCTCCTCAGGAGTTGAAGCAGGGGCAGGAACAGGTTGAACTGTATATCCACCCCTCG
CTGGAAACCTAGCCCATGCAGGAGCTTCAGTTGATCTAACCATCTTTTCACTTCACCTA
GCTGGTGTTTCTTCAATTTTAGGAGCAATTAACTTTATTACTACTATTATTAATATGAAAC
CCCCAGCCATTTCTCAATACCAAACTCCTTTATTCGTGTGGGCTGTACTTATTACAGCTG
TATTACTCCTGCTTTCACTACCCGTTCTAGCTGCTGGTATTACAATATTACTAACAGACC
GAAATTTAAATACTACCTTCTTTGATCCTGCAGGAGGTGGAGACCCAAT
>Hypostomus_sp_SZ610
GACATTGGCACCCTTTACTTAGTATTTGGTGCCTGAGCCGGAATGGTTGGCACAGCTCT
CAGCCTTTTAATTCGGGCTGAGCTAAGCCAACCCGGCTCCCTACTAGGTGATGACCAAA
TTTATAATGTCATCGTTACTGCACATGCCTTCGTAATAATTTTCTTTATAGTAATACCAATT
ATAATTGGAGGCTTCGGAAATTGACTAGTCCCACTAATGATTGGAGCACCCGACATAGC
CTTCCCACGAATAAATAATATGAGCTTCTGACTACTCCCCCCATCATTCCTTCTCCTATTA
GCCTCCTCAGGAGTTGAAGCAGGGGCAGGAACAGGTTGAACTGTATATCCACCCCTCG
CTGGAAACCTAGCCCATGCAGGAGCTTCAGTTGATCTAACCATCTTTTCACTTCACCTA
GCTGGTGTTTCTTCAATTTTAGGAGCAATTAACTTTATTACTACTATTATTAATATGAAAC
CCCCAGCCATTTCTCAATACCAAACTCCTTTATTCGTGTGGGCTGTACTTATTACAGCTG
TATTACTCCTGCTTTCACTACCCGTTCTAGCTGCTGGTATTACAATATTACTAACAGACC
GAAATTTAAATACTACCTTCTTTGATCCTGCAGGAGGTGGAGACCCAAT
>Neosalanx_sp_SZ181
GACATTGGCACCCTATATCTAATCTTCGGAGCCTGGGCAGGAATAGTGGGGACGGCCCT
TAGCCTCCTCATCCGGGCCGAACTTAGCCAACCCGGCGCCCTCCTCGGGGACGACCAG
ATCTACAATGTTATCGTCACTGCACACGCCTTCGTAATAATCTTCTTCATAGTCATACCCA

TTCTGATCGGCGGATTTGGGAACTGACTCGTTCCTCTCATGATCGGGGCCCCCGACATA
GCGTTCCCCCGAATAAACAACATGAGCTTCTGACTTCTGCCCCCCTCTTTCCTTCTACT
CCTAGCCTCCTCTGGGGTAGAAGCCGGAGCTGGGACGGGATGAACGGTGTACCCCCCT
CTTTCTGGCAACCTCGCCCATGCTGGAGCCTCGGTGGACCTCACTATCTTCTCCCTCCA
CCTTGCCGGAATCTCCTCTATCCTCGGGGCGATTAATTTCATCACAACCATCATTAACAT
GAAGCCCCCTGCTACTTCTCAGTACCAAACACCCCTGTTCGTTTGGTCTGTCCTAATTA
CTGCCGTCCTACTGCTGCTCTCTCTCCCCGTCCTAGCTGCAGGCATCACTATGCTTCTAA
CGGACCGAAACTTAAACACCACCTTCTTCGACCCAGCAGGCGGGGGGGACCCCAT
>Neosalanx_sp_SZ2011
GACATTGGCACCCTATATCTAATCTTCGGAGCCTGGGCAGGAATAGTGGGGACGGCCCT
TAGCCTCCTCATCCGGGCCGAACTTAGCCAACCCGGCGCCCTCCTCGGGGACGACCAG
ATCTACAATGTTATCGTCACTGCACACGCCTTCGTAATAATCTTCTTCATAGTCATACCCA
TTCTGATCGGCGGATTTGGGAACTGACTCGTTCCTCTCATGATCGGGGCCCCCGACATA
GCGTTCCCCCGAATAAACAACATGAGCTTCTGACTTCTGCCCCCCTCTTTCCTTCTACT
CCTAGCCTCCTCTGGGGTAGAAGCCGGAGCTGGGACGGGATGAACGGTGTACCCCCCT
CTTTCTGGCAACCTCGCCCATGCTGGAGCCTCGGTGGACCTCACTATCTTCTCCCTCCA
CCTTGCCGGAATCTCCTCTATCCTCGGGGCGATTAATTTCATCACAACCATCATTAACAT
GAAGCCCCCTGCTACTTCTCAGTACCAAACACCCCTGTTCGTTTGGTCTGTCCTAATTA
CTGCCGTCCTACTGCTGCTCTCTCTCCCCGTCCTAGCTGCAGGCATCACTATGCTTCTAA
CGGACCGAAACTTAAACACCACCTTCTTCGACCCAGCAGGCGGGGGGGACCCCAT
>Neosalanx_sp_SZ2038
GACATTGGCACCCTATATCTAATCTTCGGAGCCTGGGCAGGAATAGTGGGGACGGCCCT
TAGCCTCCTCATCCGGGCCGAACTTAGCCAACCCGGCGCCCTCCTCGGGGACGACCAG
ATCTACAATGTTATCGTCACTGCACACGCCTTCGTAATAATCTTCTTCATAGTCATACCCA
TTCTGATCGGCGGATTTGGGAACTGACTCGTTCCTCTCATGATCGGGGCCCCCGACATA
GCGTTCCCCCGAATAAACAACATGAGCTTCTGACTTCTGCCCCCCTCTTTCCTTCTACT
CCTAGCCTCCTCTGGGGTAGAAGCCGGAGCTGGGACGGGATGAACGGTGTACCCCCCT
CTTTCTGGCAACCTCGCCCATGCTGGAGCCTCGGTGGACCTCACTATCTTCTCCCTCCA
CCTTGCCGGAATCTCCTCTATCCTCGGGGCGATTAATTTCATCACAACCATCATTAACAT
GAAGCCCCCTGCTACTTCTCAGTACCAAACACCCCTGTTCGTTTGGTCTGTCCTAATTA
CTGCCGTCCTACTGCTGCTCTCTCTCCCCGTCCTAGCTGCAGGCATCACTATGCTTCTAA
CGGACCGAAACTTAAACACCACCTTCTTCGACCCAGCAGGCGGGGGGGACCCCAT
>Neosalanx_sp_SZ2758
GACATTGGCACCCTATATCTAATCTTCGGAGCCTGGGCAGGAATAGTGGGGACGGCCCT
TAGCCTCCTCATCCGGGCCGAACTTAGCCAACCCGGCGCCCTCCTCGGGGACGACCAG
ATCTACAATGTTATCGTCACTGCACACGCCTTCGTAATAATCTTCTTCATAGTCATACCCA
TTCTGATCGGCGGATTTGGGAACTGACTCGTTCCTCTCATGATCGGGGCCCCCGACATA
GCGTTCCCCCGAATAAACAACATGAGCTTCTGACTTCTGCCCCCCTCTTTCCTTCTACT
CCTAGCCTCCTCTGGGGTAGAAGCCGGAGCTGGGACGGGATGAACGGTGTACCCCCCT
CTTTCTGGCAACCTCGCCCATGCTGGAGCCTCGGTGGACCTCACTATCTTCTCCCTCCA
CCTTGCCGGAATCTCCTCTATCCTCGGGGCGATTAATTTCATCACAACCATCATTAACAT
GAAGCCCCCTGCTACTTCTCAGTACCAAACACCCCTGTTCGTTTGGTCTGTCCTAATTA
CTGCCGTCCTACTGCTGCTCTCTCTCCCCGTCCTAGCTGCAGGCATCACTATGCTTCTAA
CGGACCGAAACTTAAACACCACCTTCTTCGACCCAGCAGGCGGGGGGGACCCCAT

>Neosalanx_sp_SZ2843
GACATTGGCACCCTATATCTAATCTTCGGAGCCTGGGCAGGAATAGTGGGGACGGCCCT
TAGCCTCCTCATCCGGGCCGAACTTAGCCAACCCGGCGCCCTCCTCGGGGACGACCAG
ATCTACAATGTTATCGTCACTGCACACGCCTTCGTAATAATCTTCTTCATAGTCATACCCA
TTCTGATCGGCGGATTTGGGAACTGACTCGTTCCTCTCATGATCGGGGCCCCCGACATA
GCGTTCCCCCGAATAAACAACATGAGCTTCTGACTTCTGCCCCCCTCTTTCCTTCTACT
CCTAGCCTCCTCTGGGGTAGAAGCCGGAGCTGGGACGGGATGAACGGTGTACCCCCCT
CTTTCTGGCAACCTCGCCCATGCTGGAGCCTCGGTGGACCTCACTATCTTCTCCCTCCA
CCTTGCCGGAATCTCCTCTATCCTCGGGGCGATTAATTTCATCACAACCATCATTAACAT
GAAGCCCCCTGCTACTTCTCAGTACCAAACACCCCTGTTCGTTTGGTCTGTCCTAATTA
CTGCCGTCCTACTGCTGCTCTCTCTCCCCGTCCTAGCTGCAGGCATCACTATGCTTCTAA
CGGACCGAAACTTAAACACCACCTTCTTCGACCCAGCAGGCGGGGGGGACCCCAT
>Neosalanx_sp_SZ417
GACATTGGCACCCTATATCTAATCTTCGGAGCCTGGGCAGGAATAGTGGGGACGGCCCT
TAGCCTCCTCATCCGGGCCGAACTTAGCCAACCCGGCGCCCTCCTCGGGGACGACCAG
ATCTACAATGTTATCGTCACTGCACACGCCTTCGTAATAATCTTCTTCATAGTCATACCCA
TTCTGATCGGCGGATTTGGGAACTGACTCGTTCCTCTCATGATCGGGGCCCCCGACATA
GCGTTCCCCCGAATAAACAACATGAGCTTCTGACTTCTGCCCCCCTCTTTCCTTCTACT
CCTAGCCTCCTCTGGGGTAGAAGCCGGAGCTGGGACGGGATGAACGGTGTACCCCCCT
CTTTCTGGCAACCTCGCCCATGCTGGAGCCTCGGTGGACCTCACTATCTTCTCCCTCCA
CCTTGCCGGAATCTCCTCTATCCTCGGGGCGATTAATTTCATCACAACCATCATTAACAT
GAAGCCCCCTGCTACTTCTCAGTACCAAACACCCCTGTTCGTTTGGTCTGTCCTAATTA
CTGCCGTCCTACTGCTGCTCTCTCTCCCCGTCCTAGCTGCAGGCATCACTATGCTTCTAA
CGGACCGAAACTTAAACACCACCTTCTTCGACCCAGCAGGCGGGGGGGACCCCAT
>Oreochromis_sp1_SZ141
GACATTGGCACCCTCTATCTAGTATTTGGTGCTTGAGCCGGAATAGTAGGAACTGCACT
AAGCCTCCTAATTCGGGCAGAACTAAGCCAGCCCGGCTCTCTTCTCGGAGACGACCAA
ATCTATAATGTAATTGTTACAGCACATGCTTTCGTAATAATTTTCTTTATAGTAATACCAAT
TATGATTGGAGGCTTTGGAAACTGACTAGTACCCCTCATGATTGGTGCACCAGACATGG
CCTTCCCTCGAATAAATAACATGAGCTTTTGACTTCTCCCCCCCTCATTTCTTCTTCTTC
TCGCCTCATCTGGAGTCGAAGCAGGTGCCGGCACAGGATGGACTGTTTATCCCCCGCT
CGCAGGCAATCTTGCCCACGCTGGACCTTCTGTTGACTTAACCATCTTCTCCCTCCACT
TGGCCGGAGTGTCATCTATTTTAGGTGCAATTAATTTTATCACAACCATTATTAACATGA
AACCCCCTGCCATCTCCCAATATCAAACACCCCTATTTGTGTGATCCGTCCTAATTACCG
CAGTACTACTCCTTCTATCCCTGCCCGTTCTTGCCGCCGGCATCACAATACTTCTAACAG
ACCGAAACCTAAACACAACCTTCTTTGACCCTGCCGGAGGAGGAGACCCCAT
>Oreochromis_sp1_SZ142
GACATTGGCACCCTCTATCTAGTATTTGGTGCTTGAGCCGGAATAGTAGGAACTGCACT
AAGCCTCCTAATTCGGGCAGAACTAAGCCAGCCCGGCTCTCTTCTCGGAGACGACCAA
ATCTATAATGTAATTGTTACAGCACATGCTTTCGTAATAATTTTCTTTATAGTAATACCAAT
TATGATTGGAGGCTTTGGAAACTGACTAGTACCCCTCATGATTGGTGCACCAGACATGG
CCTTCCCTCGAATAAATAACATGAGCTTTTGACTTCTCCCCCCCTCATTTCTTCTTCTTC
TCGCCTCATCTGGAGTCGAAGCAGGTGCCGGCACAGGATGGACTGTTTATCCCCCGCT
CGCAGGCAATCTTGCCCACGCTGGACCTTCTGTTGACTTAACCATCTTCTCCCTCCACT

TGGCCGGAGTGTCATCTATTTTAGGTGCAATTAATTTTATCACAACCATTATTAACATGA
AACCCCCTGCCATCTCCCAATATCAAACACCCCTATTTGTGTGATCCGTCCTAATTACCG
CAGTACTACTCCTTCTATCCCTGCCCGTTCTTGCCGCCGGCATCACAATACTTCTAACAG
ACCGAAACCTAAACACAACCTTCTTTGACCCTGCCGGAGGAGGAGACCCCAT
>Oreochromis_sp1_SZ183
GACATTGGCACCCTCTATCTAGTATTTGGTGCTTGAGCCGGAATAGTAGGAACTGCACT
AAGCCTCCTAATTCGGGCAGAACTAAGCCAGCCCGGCTCTCTTCTCGGAGACGACCAA
ATCTATAATGTAATTGTTACAGCACATGCTTTCGTAATAATTTTCTTTATAGTAATACCAAT
TATGATTGGAGGCTTTGGAAACTGACTAGTACCCCTCATGATTGGTGCACCAGACATGG
CCTTCCCTCGAATAAATAACATGAGCTTTTGACTTCTCCCCCCCTCATTTCTTCTTCTTC
TCGCCTCATCTGGAGTCGAAGCAGGTGCCGGCACAGGATGGACTGTTTATCCCCCGCT
CGCAGGCAATCTTGCCCACGCTGGACCTTCTGTTGACTTAACCATCTTCTCCCTCCACT
TGGCCGGAGTGTCATCTATTTTAGGTGCAATTAATTTTATCACAACCATTATTAACATGA
AACCCCCTGCCATCTCCCAATATCAAACACCCCTATTTGTGTGATCCGTCCTAATTACCG
CAGTACTACTCCTTCTATCCCTGCCCGTTCTTGCCGCCGGCATCACAATACTTCTAACAG
ACCGAAACCTAAACACAACCTTCTTTGACCCTGCCGGAGGAGGAGACCCCAT
>Oreochromis_sp1_SZ197
GACATTGGCACCCTCTATCTAGTATTTGGTGCTTGAGCCGGAATAGTAGGAACTGCACT
AAGCCTCCTAATTCGGGCAGAACTAAGCCAGCCCGGCTCTCTTCTCGGAGACGACCAA
ATCTATAATGTAATTGTTACAGCACATGCTTTCGTAATAATTTTCTTTATAGTAATACCAAT
TATGATTGGAGGCTTTGGAAACTGACTAGTACCCCTCATGATTGGTGCACCAGACATGG
CCTTCCCTCGAATAAATAACATGAGCTTTTGACTTCTCCCCCCCTCATTTCTTCTTCTTC
TCGCCTCATCTGGAGTCGAAGCAGGTGCCGGCACAGGATGGACTGTTTATCCCCCGCT
CGCAGGCAATCTTGCCCACGCTGGACCTTCTGTTGACTTAACCATCTTCTCCCTCCACT
TGGCCGGAGTGTCATCTATTTTAGGTGCAATTAATTTTATCACAACCATTATTAACATGA
AACCCCCTGCCATCTCCCAATATCAAACACCCCTATTTGTGTGATCCGTCCTAATTACCG
CAGTACTACTCCTTCTATCCCTGCCCGTTCTTGCCGCCGGCATCACAATACTTCTAACAG
ACCGAAACCTAAACACAACCTTCTTTGACCCTGCCGGAGGAGGAGACCCCAT
>Oreochromis_sp1_SZ503
GACATTGGCACCCTCTATCTAGTATTTGGTGCTTGAGCCGGAATAGTAGGAACTGCACT
AAGCCTCCTAATTCGGGCAGAACTAAGCCAGCCCGGCTCTCTTCTCGGAGACGACCAA
ATCTATAATGTAATTGTTACAGCACATGCTTTCGTAATAATTTTCTTTATAGTAATACCAAT
TATGATTGGAGGCTTTGGAAACTGACTAGTACCCCTCATGATTGGTGCACCAGACATGG
CCTTCCCTCGAATAAATAACATGAGCTTTTGACTTCTCCCCCCCTCATTTCTTCTTCTTC
TCGCCTCATCTGGAGTCGAAGCAGGTGCCGGCACAGGATGGACTGTTTATCCCCCGCT
CGCAGGCAATCTTGCCCACGCTGGACCTTCTGTTGACTTAACCATCTTCTCCCTCCACT
TGGCCGGAGTGTCATCTATTTTAGGTGCAATTAATTTTATCACAACCATTATTAACATGA
AACCCCCTGCCATCTCCCAATATCAAACACCCCTATTTGTGTGATCCGTCCTAATTACCG
CAGTACTACTCCTTCTATCCCTGCCCGTTCTTGCCGCCGGCATCACAATACTTCTAACAG
ACCGAAACCTAAACACAACCTTCTTTGACCCTGCCGGAGGAGGAGACCCCAT
>Oreochromis_sp2_SZ513
GACATTGGCACCCTCTATCTAGTATTTGGTGCTTGAGCCGGAATAGTAGGAACTGCATT
AAGCCTCCTAATTCGGGCAGAACTAAGCCAGCCCGGCTCTCTCCTCGGAGACGACCAG
ATTTATAATGTAATTGTTACAGCACATGCTTTCGTAATAATTTTCTTTATAGTAATGCCAAT

TATAATTGGAGGTTTTGGAAACTGACTAGTGCCACTAATGATTGGTGCACCAGACATGG
CCTTCCCTCGAATAAATAACATGAGTTTTTGACTCCTCCCCCCCTCATTTCTCCTTCTCC
TCGCCTCATCCGGGGTCGAAGCAGGGGCCGGTACAGGATGGACTGTTTATCCCCCACT
CGCAGGCAATCTCGCCCATGCTGGGCCTTCCGTTGACTTAACCATCTTCTCCCTCCACT
TGGCCGGGGTGTCATCTATTTTAGGTGCAATTAATTTTATTACAACCATTATTAACATAAA
ACCCCCTGCCATCTCCCAATATCAAACACCCCTCTTTGTATGATCCGTTCTAATTACCGC
AGTACTACTCCTACTATCCCTACCCGTTCTTGCCGCCGGCATCACAATACTTCTAACAGA
CCGAAACCTAAACACAACCTTCTTTGACCCTGCCGGAGGAGGAGACCCCAT
>Oreochromis_sp2_SZ689
GACATTGGCACCCTCTATCTAGTATTTGGTGCTTGAGCCGGAATAGTAGGAACTGCATT
AAGCCTCCTAATTCGGGCAGAACTAAGCCAGCCCGGCTCTCTCCTCGGAGACGACCAG
ATTTATAATGTAATTGTTACAGCACATGCTTTCGTAATAATTTTCTTTATAGTAATGCCAAT
TATAATTGGAGGTTTTGGAAACTGACTAGTGCCACTAATGATTGGTGCACCAGACATGG
CCTTCCCTCGAATAAATAACATGAGTTTTTGACTCCTCCCCCCCTCATTTCTCCTTCTCC
TCGCCTCATCCGGGGTCGAAGCAGGGGCCGGTACAGGATGGACTGTTTATCCCCCACT
CGCAGGCAATCTCGCCCATGCTGGGCCTTCCGTTGACTTAACCATCTTCTCCCTCCACT
TGGCCGGGGTGTCATCTATTTTAGGTGCAATTAATTTTATTACAACCATTATTAACATAAA
ACCCCCTGCCATCTCCCAATATCAAACACCCCTCTTTGTATGATCCGTTCTAATTACCGC
AGTACTACTCCTACTATCCCTACCCGTTCTTGCCGCCGGCATCACAATACTTCTAACAGA
CCGAAACCTAAACACAACCTTCTTTGACCCTGCCGGAGGAGGAGACCCCAT
>Oreochromis_sp3_SZ296
GACATTGGCACCCTCTATCTAGTATTTGGTGCTTGAGCCGGAATAGTAGGAACCGCGCT
AAGCCTCCTAATTCGGGCAGAACTAAGCCAGCCCGGCTCTCTCCTCGGAGACGACCAG
ATTTATAATGTAATTGTTACAGCACATGCTTTTGTAATAATTTTCTTTATAGTAATGCCAAT
TATGATTGGAGGCTTTGGAAACTGACTAGTACCACTCATGATTGGTGCTCCAGATATGG
CCTTCCCTCGAATGAACAACATGAGTTTCTGACTCCTCCCTCCCTCATTCCTCCTCCTCC
TCGCCTCATCTGGAGTCGAAGCAGGTGCCGGCACAGGGTGAACTGTTTACCCCCCGCT
CGCAGGCAATCTTGCCCATGCTGGGCCTTCTGTCGACTTAACCATCTTCTCCCTCCACT
TGGCCGGGGTGTCATCTATTCTAGGCGCAATTAATTTCATTACAACAATCATTAACATGA
AACCCCCCGCCATCTCTCAATATCAAACACCTCTATTTGTATGGTCCGTTCTAATTACCG
CAGTATTACTTCTTCTATCCCTACCCGTTCTTGCCGCCGGCATCACAATACTTCTCACAG
ACCGAAACCTAAACACAACCTTCTTTGATCCTGCCGGAGGAGGAGACCCCAT
>Rhinogobius_sp_1_SZ497
GACATTGGCACCCTCTATCTTGTATTTGGTGCCTGAGCTGGAATAGTAGGTACGGCCCT
AAGCCTCCTCATTCGAGCCGAGCTAAGCCAACCCGGAGCCCTTCTGGGTGATGACCAG
ATTTACAATGTAATCGTAACAGCTCATGCTTTCGTAATAATTTTCTTTATAGTAATACCAA
TTATAATTGGAGGGTTTGGAAACTGACTAATTCCTCTGATGATCGGCGCTCCCGACATG
GCTTTCCCCCGAATGAACAACATGAGCTTTTGACTCCTGCCCCCTTCCTTCTTACTCCT
CCTGGCTTCTTCGGGAGTTGAAGCCGGGGCAGGTACCGGATGAACTGTCTACCCGCCC
CTCGCCGGGAACCTCGCCCATGCCGGCGCCTCTGTTGACTTAACCATTTTCTCTCTCCA
CTTGGCAGGCATTTCCTCTATTCTAGGGGCCATTAACTTCATTACGACCATCCTAAATAT
GAAGCCCCCTGCAATCTCACAATACCAAACCCCCCTATTCGTGTGGGCTGTGCTAATTA
CAGCTGTCCTTTTACTTCTTTCCCTCCCCGTTCTTGCCGCCGGCATTACAATGCTTCTTA
CAGACCGAAACCTAAACACGACCTTCTTTGACCCGGCAGGGGGAGGAGACCCAAT

>Rhinogobius_sp_1_SZ70
GACATTGGCACCCTCTATCTTGTATTTGGTGCCTGAGCTGGAATAGTAGGTACGGCCCT
AAGCCTCCTCATTCGAGCCGAGCTAAGCCAGCCCGGAGCCCTTCTGGGTGATGACCAG
ATTTACAATGTAATCGTAACAGCTCATGCTTTCGTAATAATTTTCTTTATAGTAATACCAA
TTATAATTGGAGGGTTTGGAAACTGACTAATTCCTCTGATGATCGGCGCTCCCGACATG
GCTTTCCCCCGAATGAACAACATGAGCTTTTGACTCCTGCCCCCTTCCTTCTTACTCCT
CCTGGCTTCTTCGGGAGTTGAAGCCGGGGCAGGTACCGGATGAACTGTCTACCCGCCC
CTCGCCGGGAACCTCGCCCATGCCGGCGCCTCTGTTGACTTAACCATTTCTCTCTCCA
CTTGGCAGGCATTTCCTCTATTCTAGGGGCCATTAACTTCATTACGACCATCCTAAATAT
GAAGCCCCCTGCAATCTCACAATACCAAACCCCCCTATTCGTGTGGGCTGTACTAATTA
CAGCTGTCCTTTTACTTCTTTCCCTCCCCGTTCTTGCCGCCGGCATTACAATACTTCTTA
CAGACCGAAACCTAAACACGACCTTCTTTGACCCGGCAGGGGGAGGAGACCCAAT
>Rhinogobius_sp1_SZ104
GACATTGGCACCCTCTATCTTGTATTTGGTGCCTGAGCTGGAATAGTAGGTACGGCCCT
AAGCCTCCTCATTCGAGCCGAGCTAAGCCAGCCCGGAGCCCTTCTGGGTGATGACCAG
ATTTACAATGTAATCGTAACAGCTCATGCTTTCGTAATAATTTTCTTTATAGTAATACCAA
TTATAATTGGAGGGTTTGGAAACTGACTAATTCCTCTGATGATCGGCGCTCCCGACATG
GCTTTCCCCCGAATGAACAACATGAGCTTTTGACTCCTGCCCCCTTCCTTCTTACTCCT
CCTGGCTTCTTCGGGAGTTGAAGCCGGGGCAGGTACCGGATGAACTGTCTACCCGCCC
CTCGCCGGGAACCTCGCCCATGCCGGCGCCTCTGTTGACTTAACCATTTCTCTCTCCA
CTTGGCAGGCATTTCCTCTATTCTAGGGGCCATTAACTTCATTACGACCATCCTAAATAT
GAAGCCCCCTGCAATCTCACAATACCAAACCCCCCTATTCGTGTGGGCTGTACTAATTA
CAGCTGTCCTTTTACTTCTTTCCCTCCCCGTTCTTGCCGCCGGCATTACAATGCTTCTTA
CAGACCGAAACCTAAACACGACCTTCTTTGACCCGGCAGGGGGAGGAGACCCAAT
>Rhinogobius_sp1_SZ105
GACATTGGCACCCTCTATCTTGTATTTGGTGCCTGAGCTGGAATAGTAGGTACGGCCCT
AAGCCTCCTCATTCGAGCCGAGCTAAGCCAGCCCGGAGCCCTTCTGGGTGATGACCAG
ATTTACAATGTAATCGTAACAGCTCATGCTTTCGTAATAATTTTCTTTATAGTAATACCAA
TTATAATTGGAGGGTTTGGAAACTGACTAATTCCTCTGATGATCGGCGCTCCCGACATG
GCTTTCCCCCGAATGAACAACATGAGCTTTTGACTCCTGCCCCCTTCCTTCTTACTCCT
CCTGGCTTCTTCGGGAGTTGAAGCCGGGGCAGGTACCGGATGAACTGTCTACCCGCCC
CTCGCCGGGAACCTCGCCCATGCCGGCGCCTCTGTTGACTTAACCATTTCTCTCTCCA
CTTGGCAGGCATTTCCTCTATTCTAGGGGCCATTAACTTCATTACGACCATCCTAAATAT
GAAGCCCCCTGCAATCTCACAATACCAAACCCCCCTATTCGTGTGGGCTGTACTAATTA
CAGCTGTCCTTTTACTTCTTTCCCTCCCCGTTCTTGCCGCCGGCATTACAATGCTTCTTA
CAGACCGAAACCTAAACACGACCTTCTTTGACCCGGCAGGGGGAGGAGACCCAAT
>Rhinogobius_sp1_SZ107
GACATTGGCACCCTCTATCTTGTATTTGGTGCCTGAGCTGGAATAGTAGGTACGGCCCT
AAGCCTCCTCATTCGAGCCGAGCTAAGCCAGCCCGGAGCCCTTCTGGGTGATGACCAG
ATTTACAATGTAATCGTAACAGCTCATGCTTTCGTAATAATTTTCTTTATAGTAATACCAA
TTATAATTGGAGGGTTTGGAAACTGACTAATTCCTCTGATGATCGGCGCTCCCGACATG
GCTTTCCCCCGAATGAACAACATGAGCTTTTGACTCCTGCCCCCTTCCTTCTTACTCCT
CCTGGCTTCTTCGGGAGTTGAAGCCGGGGCAGGTACCGGATGAACTGTCTACCCGCCC
CTCGCCGGGAACCTCGCCCATGCCGGCGCCTCTGTTGACTTAACCATTTCTCTCTCCA

CTTGGCAGGCATTTCCTCTATTCTAGGGGCCATTAACTTCATTACGACCATCCTAAATAT
GAAGCCCCCTGCAATCTCACAATACCAAACCCCCCTATTCGTGTGGGCTGTACTAATTA
CAGCTGTCCTTTTACTTCTTTCCCTCCCCGTTCTTGCCGCCGGCATTACAATGCTTCTTA
CAGACCGAAACCTAAACACGACCTTCTTTGACCCGGCAGGGGGAGGAGACCCAAT
>Rhinogobius_sp1_SZ135
GACATTGGCACCCTCTATCTTGTATTTGGTGCCTGAGCTGGAATAGTAGGTACGGCCCT
AAGCCTCCTCATTCGAGCCGAGCTAAGCCAGCCCGGAGCCCTTCTGGGTGATGACCAG
ATTTACAATGTAATCGTAACAGCTCATGCTTTCGTAATAATTTTCTTTATAGTAATACCAA
TTATAATTGGAGGGTTTGGAAACTGACTAATTCCTCTGATGATCGGCGCTCCCGACATG
GCTTTCCCCCGAATGAACAACATGAGCTTTTGACTCCTGCCCCCTTCCTTCTTACTCCT
CCTGGCTTCTTCGGGAGTTGAAGCCGGGGCAGGTACCGGATGAACTGTCTACCCGCCC
CTCGCCGGGAACCTCGCCCATGCCGGCGCCTCTGTTGACTTAACCATTTTCTCTCTCCA
CTTGGCAGGCATTTCCTCTATTCTAGGGGCCATTAACTTCATTACGACCATCCTAAATAT
GAAGCCCCCTGCAATCTCACAATACCAAACCCCCCTATTCGTGTGGGCTGTACTAATTA
CAGCTGTCCTTTTACTTCTTTCCCTCCCCGTTCTTGCCGCCGGCATTACAATGCTTCTTA
CAGACCGAAACCTAAACACGACCTTCTTTGACCCGGCAGGGGGAGGAGACCCAAT
>Rhinogobius_sp1_SZ140
GACATTGGCACCCTCTATCTTGTATTTGGTGCCTGAGCTGGAATAGTAGGTACGGCCCT
AAGCCTCCTCATTCGAGCCGAGCTAAGCCAGCCCGGAGCCCTTCTGGGTGATGACCAG
ATTTACAATGTAATCGTAACAGCTCATGCTTTCGTAATAATTTTCTTTATAGTAATACCAA
TTATAATTGGAGGGTTTGGAAACTGACTAATTCCTCTGATGATCGGCGCTCCCGACATG
GCTTTCCCCCGAATGAACAACATGAGCTTTTGACTCCTGCCCCCTTCCTTCTTACTCCT
CCTGGCTTCTTCGGGAGTTGAAGCCGGGGCAGGTACCGGATGAACTGTCTACCCGCCC
CTCGCCGGGAACCTCGCCCATGCCGGCGCCTCTGTTGACTTAACCATTTTCTCTCTCCA
CTTGGCAGGCATTTCCTCTATTCTAGGGGCCATTAACTTCATTACGACCATCCTAAATAT
GAAGCCCCCTGCAATCTCACAATACCAAACCCCCCTATTCGTGTGGGCTGTACTAATTA
CAGCTGTCCTTTTACTTCTTTCCCTCCCCGTTCTTGCCGCCGGCATTACAATGCTTCTTA
CAGACCGAAACCTAAACACGACCTTCTTTGACCCGGCAGGGGGAGGAGACCCAAT
>Rhinogobius_sp1_SZ209
GACATTGGCACCCTCTATCTTGTATTTGGTGCCTGAGCTGGAATAGTAGGTACGGCCCT
AAGCCTCCTCATTCGAGCCGAGCTAAGCCAGCCCGGAGCCCTTCTGGGTGATGACCAG
ATTTACAATGTAATCGTAACAGCTCATGCTTTCGTAATAATTTTCTTTATAGTAATACCAA
TTATAATTGGAGGGTTTGGAAACTGACTAATTCCTCTGATGATCGGCGCTCCCGACATG
GCTTTCCCCCGAATGAACAACATGAGCTTTTGACTCCTGCCCCCTTCCTTCTTACTCCT
CCTGGCTTCTTCGGGAGTTGAAGCCGGGGCAGGTACCGGATGAACTGTCTACCCGCCC
CTCGCCGGGAACCTCGCCCATGCCGGCGCCTCTGTTGACTTAACCATTTTCTCTCTCCA
CTTGGCAGGCATTTCCTCTATTCTAGGGGCCATTAACTTCATTACGACCATCCTAAATAT
GAAGCCCCCTGCAATCTCACAATACCAAACCCCCCTATTCGTGTGGGCTGTACTAATTA
CAGCTGTCCTTTTACTTCTTTCCCTCCCCGTTCTTGCCGCCGGCATTACAATGCTTCTTA
CAGACCGAAACCTAAACACGACCTTCTTTGACCCGGCAGGGGGAGGAGACCCAAT
>Rhinogobius_sp1_SZ224
GACATTGGCACCCTCTATCTTGTATTTGGTGCCTGAGCTGGAATAGTAGGTACGGCCCT
AAGCCTCCTCATTCGAGCCGAGCTAAGCCAGCCCGGAGCCCTTCTGGGTGATGACCAG
ATTTACAATGTAATCGTAACAGCTCATGCTTTCGTAATAATTTTCTTTATAGTAATACCAA

TTATAATTGGAGGGTTTGGAAACTGACTAATTCCTCTGATGATCGGCGCTCCCGACATG
GCTTTCCCCCGAATGAACAACATGAGCTTTTGACTCCTGCCCCCTTCCTTCTTACTCCT
CCTGGCTTCTTCGGGAGTTGAAGCCGGGGCAGGTACCGGATGAACTGTCTACCCGCCC
CTCGCCGGGAACCTCGCCCATGCCGGCGCCTCTGTTGACTTAACCATTTCTCTCTCCA
CTTGGCAGGCATTTCCTCTATTCTAGGGGCCATTAACTTCATTACGACCATCCTAAATAT
GAAGCCCCCTGCAATCTCACAATACCAAACCCCCCTATTCGTGTGGGCTGTACTAATTA
CAGCTGTCCTTTTACTTCTTTCCCTCCCCGTTCTTGCCGCCGGCATTACAATGCTTCTTA
CAGACCGAAACCTAAACACGACCTTCTTTGACCCGGCAGGGGGAGGAGACCCAAT
>Rhinogobius_sp1_SZ230
GACATTGGCACCCTCTATCTTGTATTTGGTGCCTGAGCTGGAATAGTAGGTACGGCCCT
AAGCCTCCTCATTCGAGCCGAGCTAAGCCAGCCCGGAGCCCTTCTGGGTGATGACCAG
ATTTACAATGTAATCGTAACAGCTCATGCTTTCGTAATAATTTTCTTTATAGTAATACCAA
TTATAATTGGAGGGTTTGGAAACTGACTAATTCCTCTGATGATCGGCGCTCCCGACATG
GCTTTCCCCCGAATGAACAACATGAGCTTTTGACTCCTGCCCCCTTCCTTCTTACTCCT
CCTGGCTTCTTCGGGAGTTGAAGCCGGGGCAGGTACCGGATGAACTGTCTACCCGCCC
CTCGCCGGGAACCTCGCCCATGCCGGCGCCTCTGTTGACTTAACCATTTCTCTCTCCA
CTTGGCAGGCATTTCCTCTATTCTAGGGGCCATTAACTTCATTACGACCATCCTAAATAT
GAAGCCCCCTGCAATCTCACAATACCAAACCCCCCTATTCGTGTGGGCTGTACTAATTA
CAGCTGTCCTTTTACTTCTTTCCCTCCCCGTTCTTGCCGCCGGCATTACAATGCTTCTTA
CAGACCGAAACCTAAACACGACCTTCTTTGACCCGGCAGGGGGAGGAGACCCAAT
>Rhinogobius_sp1_SZ362
GACATTGGCACCCTCTATCTTGTATTTGGTGCCTGAGCTGGAATAGTAGGTACGGCCCT
AAGCCTCCTCATTCGAGCCGAGCTAAGCCAACCCGGAGCCCTTCTGGGTGATGACCAG
ATTTACAATGTAATCGTAACAGCTCATGCTTTCGTAATAATTTTCTTTATAGTAATACCAA
TTATAATTGGAGGGTTTGGAAACTGACTAATTCCTCTGATGATCGGCGCTCCCGACATG
GCTTTCCCCCGAATGAACAACATGAGCTTTTGACTCCTGCCCCCTTCCTTCTTACTCCT
CCTGGCTTCTTCGGGAGTTGAAGCCGGGGCAGGTACCGGATGAACTGTCTACCCGCCC
CTCGCCGGGAACCTCGCCCATGCCGGCGCCTCTGTTGACTTAACCATTTCTCTCTCCA
CTTGGCAGGCATTTCCTCTATTCTAGGGGCCATTAACTTCATTACGACCATCCTAAATAT
GAAGCCCCCTGCAATCTCACAATACCAAACCCCCCTATTCGTGTGGGCTGTGCTAATTA
CAGCTGTCCTTTTACTTCTTTCCCTCCCCGTTCTTGCCGCCGGCATTACAATGCTTCTTA
CAGACCGAAACCTAAACACGACCTTCTTTGACCCGGCAGGGGGAGGAGACCCAAT
>Rhinogobius_sp1_SZ472
GACATTGGCACCCTCTATCTTGTATTTGGTGCCTGAGCTGGAATAGTAGGTACGGCCCT
AAGCCTCCTCATTCGAGCCGAGCTAAGCCAGCCCGGAGCCCTTCTGGGTGATGACCAG
ATTTACAATGTAATCGTAACAGCTCATGCTTTCGTAATAATTTTCTTTATAGTAATACCAA
TTATAATTGGAGGGTTTGGAAACTGACTAATTCCTCTGATGATCGGCGCTCCCGACATG
GCTTTCCCCCGAATGAACAACATGAGCTTTTGACTCCTGCCCCCTTCCTTCTTACTCCT
CCTGGCTTCTTCGGGAGTTGAAGCCGGGGCAGGTACCGGATGAACTGTCTACCCGCCC
CTCGCCGGGAACCTCGCCCATGCCGGCGCCTCTGTTGACTTAACCATTTCTCTCTCCA
CTTGGCAGGCATTTCCTCTATTCTAGGGGCCATTAACTTCATTACGACCATCCTAAATAT
GAAGCCCCCTGCAATCTCACAATACCAAACCCCCCTATTCGTGTGGGCTGTACTAATTA
CAGCTGTCCTTTTACTTCTTTCCCTCCCCGTTCTTGCCGCCGGCATTACAATGCTTCTTA
CAGACCGAAACCTAAACACGACCTTCTTTGACCCGGCAGGGGGAGGAGACCCAAT

>Rhinogobius_sp1_SZ50
GACATTGGCACCCTCTATCTTGTATTTGGTGCCTGAGCTGGAATAGTAGGTACGGCCCT
AAGCCTCCTCATTCGAGCCGAGCTAAGCCAGCCCGGAGCCCTTCTGGGTGATGACCAG
ATTTACAATGTAATCGTAACAGCTCATGCTTTCGTAATAATTTTCTTTATAGTAATACCAA
TTATAATTGGAGGGTTTGGAAACTGACTAATTCCTCTGATGATCGGCGCTCCCGACATG
GCTTTCCCCCGAATGAACAACATGAGCTTTTGACTCCTGCCCCCTTCCTTCTTACTCCT
CCTGGCTTCTTCGGGAGTTGAAGCCGGGGCAGGTACCGGATGAACTGTCTACCCGCCC
CTCGCCGGGAACCTCGCCCATGCCGGCGCCTCTGTTGACTTAACCATTTCTCTCTCCA
CTTGGCAGGCATTTCCTCTATTCTAGGGGCCATTAACTTCATTACGACCATCCTAAATAT
GAAGCCCCCTGCAATCTCACAATACCAAACCCCCCTATTCGTGTGGGCTGTACTAATTA
CAGCTGTCCTTTTACTTCTTTCCCTCCCCGTTCTTGCCGCCGGCATTACAATGCTTCTTA
CAGACCGAAACCTAAACACGACCTTCTTTGACCCGGCAGGGGGAGGAGACCCAAT
>Rhinogobius_sp1_SZ92
GACATTGGCACCCTCTATCTTGTATTTGGTGCCTGAGCTGGAATAGTAGGTACGGCCCT
AAGCCTCCTCATTCGAGCCGAGCTAAGCCAGCCCGGAGCCCTTCTGGGTGATGACCAG
ATTTACAATGTAATCGTAACAGCTCATGCTTTCGTAATAATTTTCTTTATAGTAATACCAA
TTATAATTGGAGGGTTTGGAAACTGACTAATTCCTCTGATGATCGGCGCTCCCGACATG
GCTTTCCCCCGAATGAACAACATGAGCTTTTGACTCCTGCCCCCTTCCTTCTTACTCCT
CCTGGCTTCTTCGGGAGTTGAAGCCGGGGCAGGTACCGGATGAACTGTCTACCCGCCC
CTCGCCGGGAACCTCGCCCATGCCGGCGCCTCTGTTGACTTAACCATTTCTCTCTCCA
CTTGGCAGGCATTTCCTCTATTCTAGGGGCCATTAACTTCATTACGACCATCCTAAATAT
GAAGCCCCCTGCAATCTCACAATACCAAACCCCCCTATTCGTGTGGGCTGTACTAATTA
CAGCTGTCCTTTTACTTCTTTCCCTCCCCGTTCTTGCCGCCGGCATTACAATGCTTCTTA
CAGACCGAAACCTAAACACGACCTTCTTTGACCCGGCAGGGGGAGGAGACCCAAT
>Rhinogobius_sp2_SZ115
GACATTGGCACCCTCTATCTTGTATTTGGTGCCTGAGCTGGAATAGTAGGTACGGCCCTT
AGCCTCCTCATTCGAGCCGAGCTAAGCCAGCCCGGAGCCCTTCTGGGTGATGACCAGA
TTTACAATGTAATCGTAACAGCTCATGCTTTCGTAATAATTTTCTTTATAGTAATACCAAT
TATAATTGGAGGGTTTGGAAACTGACTAATTCCTCTGATGATCGGCGCTCCCGACATGG
CTTTCCCCCGAATGAACAACATGAGCTTTTGACTCCTGCCCCCTTCCTTCTTACTCCTCC
TGGCTTCTTCGGGAGTTGAAGCCGGGGCAGGTACCGGATGAACTGTCTACCCGCCCCT
CGCCGGGAACCTCGCCCATGCCGGCGCCTCTGTTGACTTAACCATTTCTCCCTCCACT
TGGCAGGCATTTCCTCTATTCTAGGGGCCATTAACTTCATTACGACCATCCTAAATATGA
AGCCCCCTGCAATCTCACAATACCAGACCCCCCTATTCGTGTGGGCTGTACTAATTACA
GCTGTCCTTTTACTTCTTTCCCTCCCCGTTCTTGCCGCCGGCATTACAATGCTTCTTACA
GACCGAAATCTAAACACGACCTTCTTTGACCCGGCAGGGGGAGGAGACCCAAT
>Rhinogobius_sp2_SZ179
GACATTGGCACCCTCTATCTTGTATTTGGTGCCTGAGCTGGAATAGTAGGTACGGCCCTT
AGCCTCCTCATTCGAGCCGAGCTAAGCCAGCCCGGAGCCCTTCTGGGTGATGACCAGA
TTTACAATGTAATCGTAACAGCTCATGCTTTCGTAATAATTTTCTTTATAGTAATACCAAT
TATAATTGGAGGGTTTGGAAACTGACTAATTCCTCTGATGATCGGCGCTCCCGACATGG
CTTTCCCCCGAATGAACAACATGAGCTTTTGACTCCTGCCCCCTTCCTTCTTACTCCTCC
TGGCTTCTTCGGGAGTTGAAGCCGGGGCAGGTACCGGATGAACTGTCTACCCGCCCCT
CGCCGGGAACCTCGCCCATGCCGGCGCCTCTGTTGACTTAACCATTTCTCCCTCCACT

TGGCAGGCATTTCCTCTATTCTAGGGGCCATTAACTTCATTACGACCATCCTAAATATGA
AGCCCCCTGCAATCTCACAATACCAGACCCCCCTATTCGTGTGGGCTGTACTAATTACA
GCTGTCCTTTTACTTCTTTCCCTCCCCGTTCTTGCCGCCGGCATTACAATGCTTCTTACA
GACCGAAATCTAAACACGACCTTCTTTGACCCGGCAGGGGGAGGAGACCCAAT
>Rhinogobius_sp3_SZ129
GACATTGGCACCCTCTATCTTGTATTCGGTGCCTGAGCTGGAATAGTGGGAACGGCCCT
AAGCCTCCTAATTCGAGCCGAACTAAGTCAACCCGGGGCCCTCCTAGGGGACGACCAA
ATTTATAATGTAATCGTGACAGCTCATGCTTTCGTAATAATTTTCTTTATAGTAATACCAA
TTATGATCGGAGGGTTTGGAAACTGACTGATTCCTCTAATGATCGGCGCTCCCGATATA
GCCTTCCCTCGAATAAACAACATAAGCTTTTGACTCCTACCCCCTTCCTTCCTTCTCCTC
CTGGCCTCTTCCGGGGTTGAAGCTGGGGCAGGTACCGGGTGAACTGTTTACCCCCCTC
TCGCCGGGAATCTTGCCCACGCGGGAGCCTCTGTAGACCTTACCATTTTCTCCCTTCAC
CTGGCAGGTATCTCATCTATTCTTGGGGCAATTAACTTTATTACAACAATCCTGAATATG
AAACCCCCCGCAATTTCACAATATCAAACCCCTCTGTTCGTCTGAGCCGTTCTAATTAC
AGCTGTCCTGCTACTTCTTTCCCTTCCCGTACTGGCTGCAGGGATTACAATACTCCTTAC
AGACCGCAACTTAAACACAACCTTTTTCGACCCGGCAGGAGGAGGAGATCCCAT
>Rhinogobius_sp3_SZ205
GACATTGGCACCCTTTATCTTGTATTCGGTGCCTGAGCTGGAATAGTAGGAACGGCCCT
AAGCCTCCTAATTCGAGCCGAACTAAGTCAACCCGGGGCCCTCCTAGGGGACGACCAA
ATTTATAATGTAATCGTGACAGCTCATGCTTTCGTAATAATTTTCTTTATAGTAATACCAA
TTATGATCGGAGGGTTTGGAAACTGACTGATTCCTCTAATGATCGGCGCTCCCGATATA
GCCTTCCCTCGAATGAACAACATAAGCTTTTGACTCCTACCCCCCTCCTTCCTTCTCCTC
CTGGCCTCTTCCGGGGTTGAAGCTGGGGCAGGTACCGGGTGAACTGTTTACCCCCCTC
TTGCCGGGAACCTTGCCCACGCGGGAGCCTCTGTAGACCTTACCATTTTCTCCCTCCAC
CTGGCAGGTATCTCATCTATTCTTGGGGCAATTAACTTTATTACAACAATCCTGAATATG
AAACCCCCCGCAATTTCACAATATCAGACCCCTCTGTTCGTCTGGGCCGTTCTAATTAC
AGCTGTCCTGCTACTTCTTTCCCTTCCCGTACTGGCTGCAGGAATTACAATGCTCCTTAC
AGACCGCAACTTAAACACAACCTTTTTCGACCCGGCAGGCGGAGGAGATCCCAT
>Rhinogobius_sp3_SZ282
GACATTGGCACCCTTTATCTTGTATTCGGTGCCTGAGCTGGAATAGTAGGAACGGCCCT
AAGCCTCCTAATTCGAGCCGAACTAAGTCAACCCGGGGCCCTCCTAGGGGACGACCAA
ATTTATAATGTAATCGTGACAGCTCATGCTTTCGTAATAATTTTCTTTATAGTAATACCAA
TTATGATCGGAGGGTTTGGAAACTGACTGATTCCTCTAATAATCGGCGCTCCCGATATAG
CCTTCCCTCGAATGAACAACATAAGCTTTTGACTCCTACCCCCCTCCTTCCTTCTCCTCC
TGGCCTCTTCCGGGGTTGAAGCTGGGGCAGGTACCGGGTGAACTGTTTACCCCCCTCT
TGCCGGGAACCTTGCCCACGCGGGAGCCTCTGTAGACCTTACCATTTTCTCCCTCCACC
TGGCAGGTATCTCATCTATTCTTGGGGCAATTAACTTTATTACAACAATCCTGAATATGA
AACCCCCCGCAATTTCACAATATCAGACCCCTCTGTTCGTCTGGGCCGTTCTAATTACA
GCTGTCCTGCTACTTCTTTCCCTTCCCGTACTGGCTGCAGGAATTACAATGCTCCTTACA
GACCGCAACTTAAACACAACCTTTTTCGACCCGGCAGGCGGAGGAGATCCCAT
>Rhinogobius_sp3_SZ29
GACATTGGCACCCTCTATCTTGTATTCGGTGCCTGAGCTGGAATAGTGGGAACGGCCCT
AAGCCTCCTAATTCGAGCCGAACTAAGTCAACCCGGGGCCCTCCTAGGGGACGACCAA
ATTTATAATGTAATCGTGACAGCTCATGCTTTCGTAATAATTTTCTTTATAGTAATACCAA

TTATGATCGGAGGGTTTGGAAACTGACTGATTCCTCTAATGATCGGCGCTCCCGATATA
GCCTTCCCTCGAATAAACAACATAAGCTTTTGACTCCTACCCCCTTCCTTCCTTCTCCTC
CTGGCCTCTTCCGGGGTTGAAGCTGGGGCAGGTACCGGGTGAACTGTTTACCCCCCTC
TCGCCGGGAATCTTGCCCACGCGGGAGCCTCTGTAGACCTTACCATTTCTCCCTTCAC
CTGGCAGGTATCTCATCTATTCTTGGGGCAATTAACTTTATTACAACAATCCTGAATATG
AAACCCCCCGCAATTTCACAATATCAAACCCCTCTGTTCGTCTGAGCCGTTCTAATTAC
AGCTGTCCTGCTACTTCTTTCCCTTCCCGTACTGGCTGCAGGGATTACAATACTCCTTAC
AGACCGCAACTTAAACACAACCTTTTTCGACCCGGCAGGAGGAGGAGATCCCAT
>Rhinogobius_sp3_SZ314
GACATTGGCACCCTTTATCTTGTATTCGGTGCCTGAGCTGGAATAGTAGGAACGGCCCT
AAGCCTCCTAATTCGAGCCGAACTAAGTCAACCCGGGGCCCTCCTAGGGGACGACCAA
ATTTATAATGTAATCGTGACAGCTCATGCTTTCGTAATAATTTTCTTTATAGTAATACCAA
TTATGATCGGAGGGTTTGGAAACTGACTGATTCCTCTAATGATCGGCGCTCCCGATATA
GCCTTCCCTCGAATGAACAACATAAGCTTTTGACTCCTACCCCCCTCCTTCCTTCTCCTC
CTGGCCTCTTCCGGGGTTGAAGCTGGGGCAGGTACCGGGTGAACTGTTTACCCCCCTC
TTGCCGGGAACCTTGCCCACGCGGGAGCCTCTGTAGACCTTACCATTTCTCCCTCCAC
CTGGCAGGTATCTCATCTATTCTTGGGGCAATTAACTTTATTACAACAATCCTGAATATG
AAACCCCCCGCAATTTCACAATATCAGACCCCTCTGTTCGTCTGGGCCGTTCTAATTAC
AGCTGTCCTGCTACTTCTTTCCCTTCCCGTACTGGCTGCAGGAATTACAATGCTCCTTAC
AGACCGCAACTTAAACACAACCTTTTTCGACCCGGCAGGCGGAGGAGATCCCAT
>Rhinogobius_sp3_SZ316
GACATTGGCACCCTTTATCTTGTATTCGGTGCCTGAGCTGGAATAGTAGGAACGGCCCT
AAGCCTCCTAATTCGAGCCGAACTAAGTCAACCCGGGGCCCTCCTAGGGGACGACCAA
ATTTATAATGTAATCGTGACAGCTCATGCTTTCGTAATAATTTTCTTTATAGTAATACCAA
TTATGATCGGAGGGTTTGGAAACTGACTGATTCCTCTAATGATCGGCGCTCCCGATATA
GCCTTCCCTCGAATGAACAACATAAGCTTTTGACTCCTACCCCCCTCCTTCCTTCTCCTC
CTGGCCTCTTCCGGGGTTGAAGCTGGGGCAGGTACCGGGTGAACTGTTTACCCCCCTC
TTGCCGGGAACCTTGCCCACGCGGGAGCCTCTGTAGACCTTACCATTTCTCCCTCCAC
CTGGCAGGTATCTCATCTATTCTTGGGGCAATTAACTTTATTACAACAATCCTGAATATG
AAACCCCCCGCAATTTCACAATATCAGACCCCTCTGTTCGTCTGGGCCGTTCTAATTAC
AGCTGTCCTGCTACTTCTTTCCCTTCCCGTACTGGCTGCAGGAATTACAATGCTCCTTAC
AGACCGCAACTTAAACACAACCTTTTTCGACCCGGCAGGCGGAGGAGATCCCAT
>Rhinogobius_sp3_SZ318
GACATTGGCACCCTTTATCTTGTATTCGGTGCCTGGGCTGGAATAGTAGGAACGGCCCT
AAGCCTCCTAATTCGAGCCGAACTAAGTCAACCCGGGGCCCTCCTAGGGGACGACCAA
ATTTATAATGTAATCGTGACAGCTCATGCTTTCGTAATAATTTTCTTTATAGTAATACCAA
TTATGATCGGAGGGTTTGGAAACTGACTGATTCCTCTAATGATCGGCGCTCCCGATATA
GCCTTCCCTCGAATGAACAACATAAGCTTTTGACTCCTACCCCCCTCCTTCCTTCTCCTC
CTGGCCTCTTCCGGGGTTGAAGCTGGGGCAGGTACCGGGTGAACTGTTTACCCCCCTC
TTGCCGGGAACCTTGCCCACGCGGGAGCCTCTGTAGACCTTACCATTTCTCCCTCCAC
CTGGCAGGTATCTCATCTATTCTTGGGGCAATTAACTTTATTACAACAATCCTGAATATG
AAACCCCCCGCAATTTCACAATATCAGACCCCTCTGTTCGTCTGGGCCGTTCTAATTAC
AGCTGTCCTGCTACTTCTTTCCCTTCCCGTACTGGCTGCAGGAATTACAATGCTCCTTAC
AGACCGCAACTTAAACACAACCTTTTTCGACCCGGCAGGAGGAGGAGATCCCAT

>Rhinogobius_sp3_SZ623
GACATTGGCACCCTTTATCTTGTATTCGGTGCCTGAGCTGGAATAGTAGGAACGGCCCT
AAGCCTCCTAATTCGAGCCGAACTAAGTCAACCCGGGGCCCTCCTAGGGGACGACCAA
ATTTATAATGTAATCGTGACAGCTCATGCTTTCGTAATAATTTTCTTTATAGTAATACCAA
TTATGATCGGAGGGTTTGGAAACTGACTGATTCCCTTAATGATCGGCGCTCCCGATATA
GCCTTCCCTCGAATGAACAACATAAGCTTTTGACTCCTACCCCCCTCCTTCCTTCTCCTC
CTGGCCTCTTCCGGGGTTGAAGCTGGGGCAGGTACCGGGTGAACTGTTTACCCCCCTC
TTGCCGGGAACCTTGCCCACGCGGGAGCCTCTGTAGACCTTACCATTTTCTCCCTTCAC
CTGGCAGGTATCTCATCTATTCTTGGGGCAATTAACTTTATTACAACAATCCTGAATATG
AAACCCCCCGCAATTTCACAATATCAAACCCCTCTGTTCGTCTGAGCCGTTCTAATTAC
AGCTGTCCTGCTACTTCTTTCCCTCCCCGTACTGGCTGCAGGAATTACAATGCTCCTTAC
AGACCGCAACTTAAACACAACCTTTTTCGACCCGGCAGGGGGAGGAGATCCCAT
>Rhinogobius_sp3_SZ626
GACATTGGCACCCTTTATCTTGTATTCGGTGCCTGGGCTGGAATAGTAGGAACGGCCCT
AAGCCTCCTAATTCGAGCCGAACTAAGTCAACCCGGGGCCCTCCTAGGGGACGACCAA
ATTTATAATGTAATCGTGACAGCTCATGCTTTCGTAATAATTTTCTTTATAGTAATACCAA
TTATGATCGGAGGGTTTGGAAACTGACTGATTCCTCTAATGATCGGCGCTCCCGATATA
GCCTTCCCTCGAATGAACAACATAAGCTTTTGACTCCTACCCCCCTCCTTCCTTCTCCTC
CTGGCCTCTTCCGGGGTTGAAGCTGGGGCAGGTACCGGGTGAACTGTTTACCCCCCTC
TTGCCGGGAACCTTGCCCACGCGGGAGCCTCTGTAGACCTTACCATTTTCTCCCTCCAC
CTGGCAGGTATCTCATCTATTCTTGGGGCAATTAACTTTATTACAACAATCCTGAATATG
AAACCCCCCGCAATTTCACAATATCAGACCCCTCTGTTCGTCTGGGCCGTTCTAATTAC
AGCTGTCCTGCTACTTCTTTCCCTTCCCGTACTGGCTGCAGGAATTACAATGCTCCTTAC
AGACCGCAACTTAAACACAACCTTTTTCGACCCGGCAGGAGGAGGAGACCCCAT
>Rhinogobius_sp3_SZ631
GACATTGGCACCCTTTATCTTGTATTCGGTGCCTGAGCTGGAATAGTAGGAACGGCCCT
AAGCCTCCTAATTCGAGCCGAACTAAGTCAACCCGGGGCCCTCCTAGGGGACGACCAA
ATTTATAATGTAATCGTGACAGCTCATGCTTTCGTAATAATTTTCTTTATAGTAATACCAA
TTATGATCGGAGGGTTTGGAAACTGACTGATTCCTCTAATAATCGGCGCTCCCGATATAG
CCTTCCCTCGAATGAACAACATAAGCTTTTGACTCCTACCCCCCTCCTTCCTTCTCCTCC
TGGCCTCTTCCGGGGTTGAAGCTGGGGCAGGTACCGGGTGAACTGTTTACCCCCCTCT
TGCCGGGAACCTTGCCCACGCGGGAGCCTCTGTAGACCTTACCATTTTCTCCCTCCACC
TGGCAGGTATCTCATCTATTCTTGGGGCAATTAACTTTATTACAACAATCCTGAATATGA
AACCCCCCGCAATTTCACAATATCAGACCCCTCTGTTCGTCTGGGCCGTTCTAATTACA
GCTGTCCTGCTACTTCTTTCCCTTCCCGTACTGGCTGCAGGAATTACAATGCTCCTTACA
GACCGCAACTTAAACACAACCTTTTTCGACCCGGCAGGCGGAGGAGATCCCAT
>Rhinogobius_sp3_SZ670
GACATTGGCACCCTTTATCTTGTATTCGGTGCCTGAGCTGGAATAGTAGGAACGGCCCT
AAGCCTCCTAATTCGAGCCGAACTAAGTCAACCCGGGGCCCTCCTAGGGGACGACCAA
ATTTATAATGTAATCGTGACAGCTCATGCTTTCGTAATAATTTTCTTTATAGTAATACCAA
TTATGATCGGAGGGTTTGGAAACTGACTGATTCCCTTAATGATCGGCGCTCCCGATATA
GCCTTCCCTCGAATGAACAACATAAGCTTTTGACTCCTACCCCCCTCCTTCCTTCTCCTC
CTGGCCTCTTCCGGGGTTGAAGCTGGGGCAGGTACCGGGTGAACTGTTTACCCCCCTC
TTGCCGGGAACCTTGCCCACGCGGGAGCCTCTGTAGACCTTACCATTTTCTCCCTTCAC

CTGGCAGGTATCTCATCTATTCTTGGGGCAATTAACTTTATTACAACAATCCTGAATATA
AAACCCCCCGCAATTTCACAATATCAAACCCCTCTGTTCGTCTGAGCCGTTCTAATTAC
AGCCGTCCTGCTACTTCTTTCCCTCCCCGTACTGGCTGCAGGAATTACAATGCTCCTTA
CAGACCGCAACTTAAACACAACCTTTTTCGACCCGGCAGGGGGAGGAGATCCCAT
>Rhinogobius_sp4_SZ136
GACATTGGCACCCTTTATCTTGTATTTGGTGCCTGAGCTGGAATAGTGGGTACGGCCTT
GAGCCTCCTTATTCGAGCCGAACTAAGCCAGCCCGGAGCCCTTCTGGGCGATGACCAA
ATCTACAACGTTATCGTTACAGCTCATGCCTTCGTAATAATTTTCTTTATAGTAATACCAA
TCATGATTGGAGGATTCGGAAACTGGCTAGTCCCCCTGATAATTGGCGCACCAGACATG
GCCTTCCCTCGAATGAACAACATAAGCTTCTGACTTCTGCCCCCCTCGTTCCTACTACTA
TTAGCTTCTTCAGGAGTTGAAGCGGGAGCAGGCACCGGATGAACTGTCTACCCCCCAC
TAGCCGGAAACCTCGCCCATGCCGGAGCCTCCGTTGACTTAACCATTTTCTCCCTTCAT
CTGGCTGGTATTTCCTCCATCCTTGGGGCCATTAACTTCATCACAACTATTATTAACATG
AAGCCTCCTGCAATCTCACAATACCAGACCCCCCTTTTCGTGTGAGCAGTTCTCATTAC
AGCCGTACTCCTACTTCTCTCCCTTCCGGTTCTTGCTGCCGGCATCACAATACTTCTTAC
AGACCGGAACCTAAATACAACCTTCTTTGACCCGGCAGGCGGAGGAGACCCAAT
>Rhinogobius_sp4_SZ196
GACATTGGCACCCTTTATCTTGTATTTGGTGCCTGAGCTGGAATAGTGGGTACGGCCTT
GAGCCTCCTTATTCGAGCCGAACTAAGCCAGCCCGGAGCCCTTCTGGGCGATGACCAA
ATCTACAACGTTATCGTTACAGCTCATGCCTTCGTAATAATTTTCTTTATAGTAATACCAA
TCATGATTGGAGGATTCGGAAACTGGCTAGTCCCCCTGATAATTGGCGCACCAGACATG
GCCTTCCCTCGAATGAACAACATAAGCTTCTGACTTCTGCCCCCCTCGTTCCTACTACTA
TTAGCTTCTTCAGGAGTTGAAGCGGGAGCAGGCACCGGATGAACTGTCTACCCCCCAC
TAGCCGGAAACCTCGCCCATGCCGGAGCCTCCGTTGACTTAACCATTTTCTCCCTTCAT
CTGGCTGGTATTTCCTCCATCCTTGGGGCCATTAACTTCATCACAACTATTATTAACATG
AAGCCTCCTGCAATCTCACAATACCAGACCCCCCTTTTCGTGTGAGCAGTTCTCATTAC
AGCCGTACTCCTACTTCTCTCCCTTCCGGTTCTTGCTGCCGGCATCACAATACTTCTTAC
AGACCGGAACCTAAATACAACCTTCTTTGACCCGGCAGGCGGAGGAGACCCAAT
>Rhinogobius_sp4_SZ220
GACATTGGCACCCTTTATCTTGTATTTGGTGCCTGAGCTGGAATAGTGGGTACGGCCTT
GAGCCTCCTTATTCGAGCCGAACTAAGCCAGCCCGGAGCCCTTCTGGGCGATGACCAA
ATCTACAACGTTATCGTTACAGCTCATGCCTTCGTAATAATTTTCTTTATAGTAATACCAA
TCATGATTGGAGGATTCGGAAACTGGCTAGTCCCCCTGATAATTGGCGCACCAGACATG
GCCTTCCCTCGAATGAACAACATAAGCTTCTGACTTCTGCCCCCCTCGTTCCTACTACTA
TTAGCTTCTTCAGGAGTTGAAGCGGGAGCAGGCACCGGATGAACTGTCTACCCCCCAC
TAGCCGGAAACCTCGCCCATGCCGGAGCCTCCGTTGACTTAACCATTTTCTCCCTTCAT
CTGGCTGGTATTTCCTCCATCCTTGGGGCCATTAACTTCATCACAACTATTATTAACATG
AAGCCTCCTGCAATCTCACAATACCAGACCCCCCTTTTCGTGTGAGCAGTTCTCATTAC
AGCCGTACTCCTACTTCTCTCCCTTCCGGTTCTTGCTGCCGGCATCACAATACTTCTTAC
AGACCGGAACCTAAATACAACCTTCTTTGACCCGGCAGGCGGAGGAGACCCAAT
>Rhinogobius_sp4_SZ227
GACATTGGCACCCTTTATCTTGTATTTGGTGCCTGAGCTGGAATAGTGGGTACGGCCTT
GAGCCTCCTTATTCGAGCCGAACTAAGCCAGCCCGGAGCCCTTCTGGGCGATGACCAA
ATCTACAACGTTATCGTTACAGCTCATGCCTTCGTAATAATTTTCTTTATAGTAATACCAA

TCATGATTGGAGGATTCGGAAACTGGCTAGTCCCCCTGATAATTGGCGCACCAGACATG
GCCTTCCCTCGAATGAACAACATAAGCTTCTGACTTCTGCCCCCCTCGTTCCTACTACTA
TTAGCTTCTTCAGGAGTTGAAGCGGGAGCAGGCACCGGATGAACTGTCTACCCCCCAC
TAGCCGGAAACCTCGCCCATGCCGGAGCCTCCGTTGACTTAACCATTTCTCCCTTCAT
CTGGCTGGTATTTCCTCCATCCTTGGGGCCATTAACTTCATCACAACTATTATTAACATG
AAGCCTCCTGCAATCTCACAATACCAGACCCCCCTTTTCGTGTGAGCAGTTCTCATTAC
AGCCGTACTCCTACTTCTCTCCCTTCCGGTTCTTGCTGCCGGCATCACAATACTTCTTAC
AGACCGGAACCTAAATACAACCTTCTTTGACCCGGCAGGCGGAGGAGACCCAAT
>Rhinogobius_sp4_SZ2736
GACATTGGCACCCTTTATCTTGTATTTGGTGCCTGAGCTGGAATAGTGGGTACGGCCTT
GAGCCTCCTTATTCGAGCCGAACTAAGCCAGCCCGGAGCCCTTCTGGGCGATGACCAA
ATCTACAACGTTATCGTTACAGCTCATGCCTTCGTAATAATTTTCTTTATAGTAATACCAA
TCATGATTGGAGGATTCGGAAACTGGCTAGTCCCCCTGATAATTGGCGCACCAGACATG
GCCTTCCCTCGAATGAACAACATAAGCTTCTGACTTCTGCCCCCCTCGTTCCTACTACTA
TTAGCTTCTTCAGGAGTTGAAGCGGGAGCAGGCACCGGATGAACTGTCTACCCCCCAC
TAGCCGGAAACCTCGCCCaTGCCGGAGCCTCCGTTGACTTAACCATTTCTCCCTTCAT
CTGGCTGGTATTTCCTCCATCCTTGGGGCCATTAACTTCATCACAACTATTATTAACATG
AAGCCTCCTGCAATCTCACAATACCAGACCCCCCTTTTCGTGTGAGCAGTTCTCATTAC
AGCCGTACTCCTACTTCTCTCCCTTCCGGTTCTTGCTGCCGGCATCACAATACTTCTTAC
AGACCGGAACCTAAATACAACCTTCTTTGACCCGGCAGGCGGAGGAGACCCAAT
>Rhinogobius_sp4_SZ2761
GACATTGGCACCCTTTATCTTGTATTTGGTGCCTGAGCTGGAATAGTGGGTACGGCCTT
GAGCCTCCTTATTCGAGCCGAACTAAGCCAGCCCGGAGCCCTTCTGGGCGATGACCAA
ATCTACAACGTTATCGTTACAGCTCATGCCTTCGTAATAATTTTCTTTATAGTAATACCAA
TCATGATTGGAGGATTCGGAAACTGGCTAGTCCCCCTGATAATTGGCGCACCAGACATG
GCCTTCCCTCGAATGAACAACATAAGCTTCTGACTTCTGCCCCCCTCGTTCCTACTACTA
TTAGCTTCTTCAGGAGTTGAAGCGGGAGCAGGCACCGGATGAACTGTCTACCCCCCAC
TAGCCGGAAACCTCGCCCATGCCGGAGCCTCCGTTGACTTAACCATTTCTCCCTTCAT
CTGGCTGGTATTTCCTCCATCCTTGGGGCCATTAACTTCATCACAACTATTATTAACATG
AAGCCTCCTGCAATCTCACAATACCAGACCCCCCTTTTCGTGTGAGCAGTTCTCATTAC
AGCCGTACTCCTACTTCTCTCCCTTCCGGTTCTTGCTGCCGGCATCACAATACTTCTTAC
AGACCGGAACCTAAATACAACCTTCTTTGACCCGGCAGGCGGAGGAGACCCAAT
>Rhinogobius_sp4_SZ2804
GACATTGGCACCCTTTATCTTGTATTTGGTGCCTGAGCTGGAATAGTGGGTACGGCCTT
GAGCCTCCTTATTCGAGCCGAACTAAGCCAGCCCGGAGCCCTTCTGGGCGATGACCAA
ATCTACAACGTTATCGTTACAGCTCATGCCTTCGTAATAATTTTCTTTATAGTAATACCAA
TCATGATTGGAGGATTCGGAAACTGGCTAGTCCCCCTGATAATTGGCGCACCAGACATG
GCCTTCCCTCGAATGAACAACATAAGCTTCTGACTTCTGCCCCCCTCGTTCCTACTACTA
TTAGCTTCTTCAGGAGTTGAAGCGGGAGCAGGCACCGGATGAACTGTCTACCCCCCAC
TAGCCGGAAACCTCGCCCATGCCGGAGCCTCCGTTGACTTAACCATTTCTCCCTTCAT
CTGGCTGGTATTTCCTCCATCCTTGGGGCCATTAACTTCATCACAACTATTATTAACATG
AAGCCTCCTGCAATCTCACAATACCAGACCCCCCTTTTCGTGTGAGCAGTTCTCATTAC
AGCCGTACTCCTACTTCTCTCCCTTCCGGTTCTTGCTGCCGGCATCACAATACTTCTTAC
AGACCGGAACCTAAATACAACCTTCTTTGACCCGGCAGGCGGAGGAGACCCAAT

>Rhinogobius_sp4_SZ2882
GACATTGGCACCCTTTATCTTGTATTTGGTGCCTGAGCTGGAATAGTGGGTACGGCCTT
GAGCCTCCTTATTCGAGCCGAATTAAGCCAGCCCGGAGCCCTTCTGGGCGATGACCAA
ATCTATAACGTTATCGTTACAGCTCATGCCTTCGTAATAATTTTCTTTATAGTAATGCCAA
TCATGATTGGAGGCTTCGGAAACTGGCTAGTCCCCCTGATAATTGGCGCACCAGACATG
GCCTTCCCTCGAATGAACAACATAAGTTTCTGACTTCTACCCCCCTCGTTCCTACTACTA
TTAGCTTCTTCAGGAGTTGAAGCGGGAGCAGGCACCGGATGGACTGTCTACCCCCCAC
TAGCCGGAAACCTCGCCCATGCCGGAGCCTCCGTTGACTTAACCATTTTCTCCCTTCAT
CTGGCTGGTATTTCCTCCATCCTTGGGGCCATTAACTTCATCACAACTATTATTAACATG
AAGCCTCCTGCAATCTCACAATACCAAACCCCTCTTTTCGTGTGAGCAGTTCTCATTAC
AGCCGTACTCCTACTTCTCTCCCTTCCGGTTCTTGCTGCCGGCATCACAATACTTCTCAC
AGACCGGAACCTAAATACAACCTTCTTTGACCCGGCAGGGGGAGGAGACCCAAT
>Rhinogobius_sp4_SZ2883
GACATTGGCACCCTTTATCTTGTATTTGGTGCCTGAGCTGGAATAGTGGGTACGGCCTT
GAGCCTCCTTATTCGAGCCGAATTAAGCCAGCCCGGAGCCCTTCTGGGCGATGACCAA
ATCTATAACGTTATCGTTACAGCTCATGCCTTCGTAATAATTTTCTTTATAGTAATGCCAA
TCATGATTGGAGGCTTCGGAAACTGGCTAGTCCCCCTGATAATTGGCGCACCAGACATG
GCCTTCCCTCGAATGAACAACATAAGTTTCTGACTTCTACCCCCCTCGTTCCTACTACTA
TTAGCTTCTTCAGGAGTTGAAGCGGGAGCAGGCACCGGATGGACTGTCTACCCCCCAC
TAGCCGGAAACCTCGCCCATGCCGGAGCCTCCGTTGACTTAACCATTTTCTCCCTTCAT
CTGGCTGGTATTTCCTCCATCCTTGGGGCCATTAACTTCATCACAACTATTATTAACATG
AAGCCTCCTGCAATCTCACAATACCAAACCCCTCTTTTCGTGTGAGCAGTTCTCATTAC
AGCCGTACTCCTACTTCTCTCCCTTCCGGTTCTTGCTGCCGGCATCACAATACTTCTCAC
AGACCGGAACCTAAATACAACCTTCTTTGACCCGGCAGGGGGAGGAGACCCAAT
>Rhinogobius_sp4_SZ291
GACATTGGCACCCTTTATCTTGTATTTGGTGCCTGAGCTGGAATAGTGGGTACGGCCTT
GAGCCTCCTTATTCGAGCCGAACTAAGCCAGCCCGGAGCCCTTCTGGGCGATGACCAA
ATCTACAACGTTATCGTTACAGCTCATGCCTTCGTAATAATTTTCTTTATAGTAATACCAA
TCATGATTGGAGGATTCGGAAACTGGCTAGTCCCCCTGATAATTGGCGCACCAGACATG
GCCTTCCCTCGAATGAACAACATAAGCTTCTGACTTCTGCCCCCCTCGTTCCTACTACTA
TTAGCTTCTTCAGGAGTTGAAGCGGGAGCAGGCACCGGATGAACTGTCTACCCCCCAC
TAGCCGGAAACCTCGCCCATGCCGGAGCCTCCGTTGACTTAACCATTTTCTCCCTTCAT
CTGGCTGGTATTTCCTCCATCCTTGGGGCCATTAACTTCATCACAACTATTATTAACATG
AAGCCTCCTGCAATCTCACAATACCAGACCCCCCTTTTCGTGTGAGCAGTTCTCATTAC
AGCCGTACTCCTACTTCTCTCCCTTCCGGTTCTTGCTGCCGGCATCACAATACTTCTTAC
AGACCGGAACCTAAATACAACCTTCTTTGACCCGGCAGGCGGAGGAGACCCAAT
>Rhinogobius_sp4_SZ321
GACATTGGCACCCTTTATCTTGTATTTGGTGCCTGAGCTGGAATAGTGGGTACGGCCTT
GAGCCTCCTTATTCGAGCCGAACTAAGCCAGCCCGGAGCCCTTCTGGGCGATGACCAA
ATCTACAACGTTATCGTTACAGCTCATGCCTTCGTAATAATTTTCTTTATAGTAATACCAA
TCATGATTGGAGGATTCGGAAACTGGCTAGTCCCCCTGATAATTGGCGCACCAGACATG
GCCTTCCCTCGAATGAACAACATAAGCTTCTGACTTCTGCCCCCCTCGTTCCTACTACTA
TTAGCTTCTTCAGGAGTTGAAGCGGGAGCAGGCACCGGATGAACTGTCTACCCCCCAC
TAGCCGGAAACCTCGCCCATGCCGGAGCCTCCGTTGACTTAACCATTTTCTCCCTTCAT

CTGGCTGGTATTTCCTCCATCCTTGGGGCCATTAACTTCATCACAACTATTATTAACATG
AAGCCTCCTGCAATCTCACAATACCAGACCCCCCTTTTCGTGTGAGCAGTTCTCATTAC
AGCCGTACTCCTACTTCTCTCCCTTCCGGTTCTTGCTGCCGGCATCACAATACTTCTTAC
AGACCGGAACCTAAATACAACCTTCTTTGACCCGGCAGGCGGAGGAGACCCAAT
>Rhinogobius_sp4_SZ322
GACATTGGCACCCTTTATCTTGTATTTGGTGCCTGAGCTGGAATAGTGGGTACGGCCTT
GAGCCTCCTTATTCGAGCCGAACTAAGCCAGCCCGGAGCCCTTCTGGGCGATGACCAA
ATCTACAACGTTATCGTTACAGCTCATGCCTTCGTAATAATTTTCTTTATAGTAATACCAA
TCATGATTGGAGGATTCGGAAACTGGCTAGTCCCCCTGATAATTGGCGCACCAGACATG
GCCTTCCCTCGAATGAACAACATAAGCTTCTGACTTCTGCCCCCCTCGTTCCTACTACTA
TTAGCTTCTTCAGGAGTTGAAGCGGGAGCAGGCACCGGATGAACTGTCTACCCCCCAC
TAGCCGGAAACCTCGCCCATGCCGGAGCCTCCGTTGACTTAACCATTTTCTCCCTTCAT
CTGGCTGGTATTTCCTCCATCCTTGGGGCCATTAACTTCATCACAACTATTATTAACATG
AAGCCTCCTGCAATCTCACAATACCAGACCCCCCTTTTCGTGTGAGCAGTTCTCATTAC
AGCCGTACTCCTACTTCTCTCCCTTCCGGTTCTTGCTGCCGGCATCACAATACTTCTTAC
AGACCGGAACCTAAATACAACCTTCTTTGACCCGGCAGGCGGAGGAGACCCAAT
>Rhinogobius_sp4_SZ335
GACATTGGCACCCTTTATCTTGTATTTGGTGCCTGAGCTGGAATAGTGGGTACGGCCTT
GAGCCTCCTTATTCGAGCCGAACTAAGCCAGCCCGGAGCCCTTCTGGGCGATGACCAA
ATCTACAACGTTATCGTTACAGCTCATGCCTTCGTAATAATTTTCTTTATAGTAATACCAA
TCATGATTGGAGGATTCGGAAACTGGCTAGTCCCCCTGATAATTGGCGCACCAGACATG
GCCTTCCCTCGAATGAACAACATAAGCTTCTGACTTCTGCCCCCCTCGTTCCTACTACTA
TTAGCTTCTTCAGGAGTTGAAGCGGGAGCAGGCACCGGATGAACTGTCTACCCCCCAC
TAGCCGGAAACCTCGCCCATGCCGGAGCCTCCGTTGACTTAACCATTTTCTCCCTTCAT
CTGGCTGGTATTTCCTCCATCCTTGGGGCCATTAACTTCATCACAACTATTATTAACATG
AAGCCTCCTGCAATCTCACAATACCAGACCCCCCTTTTCGTGTGAGCAGTTCTCATTAC
AGCCGTACTCCTACTTCTCTCCCTTCCGGTTCTTGCTGCCGGCATCACAATACTTCTTAC
AGACCGGAACCTAAATACAACCTTCTTTGACCCGGCAGGCGGAGGAGACCCAAT
>Rhinogobius_sp4_SZ403
GACATTGGCACCCTTTATCTTGTATTTGGTGCCTGAGCTGGAATAGTGGGTACGGCCTT
GAGCCTCCTTATTCGAGCCGAACTAAGCCAGCCCGGAGCCCTTCTGGGCGATGACCAA
ATCTACAACGTTATCGTTACAGCTCATGCCTTCGTAATAATTTTCTTTATAGTAATACCAA
TCATGATTGGAGGATTCGGAAACTGGCTAGTCCCCCTGATAATTGGCGCACCAGACATG
GCCTTCCCTCGAATGAACAACATAAGCTTCTGACTTCTGCCCCCCTCGTTCCTACTACTA
TTAGCTTCTTCAGGAGTTGAAGCGGGAGCAGGCACCGGATGAACTGTCTACCCCCCAC
TAGCCGGAAACCTCGCCCATGCCGGAGCCTCCGTTGACTTAACCATTTTCTCCCTTCAT
CTGGCTGGTATTTCCTCCATCCTTGGGGCCATTAACTTCATCACAACTATTATTAACATG
AAGCCTCCTGCAATCTCACAATACCAGACCCCCCTTTTCGTGTGAGCAGTTCTCATTAC
AGCCGTACTCCTACTTCTCTCCCTTCCGGTTCTTGCTGCCGGCATCACAATACTTCTTAC
AGACCGGAACCTAAATACAACCTTCTTTGACCCGGCAGGCGGAGGAGACCCAAT
>Rhinogobius_sp4_SZ52
GACATTGGCACCCTTTATCTTGTATTTGGTGCCTGAGCTGGAATAGTGGGTACGGCCTT
GAGCCTCCTTATTCGAGCCGAACTAAGCCAGCCCGGAGCCCTTCTGGGCGATGACCAA
ATCTACAACGTTATCGTTACAGCTCATGCCTTCGTAATAATTTTCTTTATAGTAATACCAA

TCATGATTGGAGGATTCGGAAACTGGCTAGTCCCCCTGATAATTGGCGCACCAGACATG
GCCTTCCCTCGAATGAACAACATAAGCTTCTGACTTCTGCCCCCCTCGTTCCTACTACTA
TTAGCTTCTTCAGGAGTTGAAGCGGGAGCAGGCACCGGATGAACTGTCTACCCCCCAC
TAGCCGGAAACCTCGCCCATGCCGGAGCCTCCGTTGACTTAACCATTTTCTCCCTTCAT
CTGGCTGGTATTTCCTCCATCCTTGGGGCCATTAACTTCATCACAACTATTATTAACATG
AAGCCTCCTGCAATCTCACAATACCAGACCCCCCTTTTCGTGTGAGCAGTTCTCATTAC
AGCCGTACTCCTACTTCTCTCCCTTCCGGTTCTTGCTGCCGGCATCACAATACTTCTTAC
AGACCGGAACCTAAATACAACCTTCTTTGACCCGGCAGGCGGAGGAGACCCAAT
>Rhinogobius_sp4_SZ538
GACATTGGCACCCTTTATCTTGTATTTGGTGCCTGAGCTGGAATAGTGGGTACGGCCTT
GAGCCTCCTTATTCGAGCCGAACTAAGCCAGCCCGGAGCCCTTCTGGGCGATGACCAA
ATCTACAACGTTATCGTTACAGCTCATGCCTTCGTAATAATTTTCTTTATAGTAATACCAA
TCATGATTGGAGGATTCGGAAACTGGCTAGTCCCCCTGATAATTGGCGCACCAGACATG
GCCTTCCCTCGAATGAACAACATAAGCTTCTGACTTCTGCCCCCCTCGTTCCTACTACTA
TTAGCTTCTTCAGGAGTTGAAGCGGGAGCAGGCACCGGATGAACTGTCTACCCCCCAC
TAGCCGGAAACCTCGCCCATGCCGGAGCCTCCGTTGACTTAACCATTTTCTCCCTTCAT
CTGGCTGGTATTTCCTCCATCCTTGGGGCCATTAACTTCATCACAACTATTATTAACATG
AAGCCTCCTGCAATCTCACAATACCAGACCCCCCTTTTCGTGTGAGCAGTTCTCATTAC
AGCCGTACTCCTACTTCTCTCCCTTCCGGTTCTTGCTGCCGGCATCACAATACTTCTTAC
AGACCGGAACCTAAATACAACCTTCTTTGACCCGGCAGGCGGAGGAGACCCAAT
>Rhinogobius_sp4_SZ577
GACATTGGCACCCTTTATCTTGTATTTGGTGCCTGAGCTGGAATAGTGGGTACGGCCTT
GAGCCTCCTTATTCGAGCCGAACTAAGCCAGCCCGGAGCCCTTCTGGGCGATGACCAA
ATCTACAACGTTATCGTTACAGCTCATGCCTTCGTAATAATTTTCTTTATAGTAATACCAA
TCATGATTGGAGGATTCGGAAACTGGCTAGTCCCCCTGATAATTGGCGCACCAGACATG
GCCTTCCCTCGAATGAACAACATAAGCTTCTGACTTCTGCCCCCCTCGTTCCTACTACTA
TTAGCTTCTTCAGGAGTTGAAGCGGGAGCAGGCACCGGATGAACTGTCTACCCCCCAC
TAGCCGGAAACCTCGCCCATGCCGGAGCCTCCGTTGACTTAACCATTTTCTCCCTTCAT
CTGGCTGGTATTTCCTCCATCCTTGGGGCCATTAACTTCATCACAACTATTATTAACATG
AAGCCTCCTGCAATCTCACAATACCAGACCCCCCTTTTCGTGTGAGCAGTTCTCATTAC
AGCCGTACTCCTACTTCTCTCCCTTCCGGTTCTTGCTGCCGGCATCACAATACTTCTTAC
AGACCGGAACCTAAATACAACCTTCTTTGACCCGGCAGGCGGAGGAGACCCAAT
>Rhinogobius_sp4_SZ7
GACATTGGCACCCTTTATCTTGTATTTGGTGCCTGAGCTGGAATAGTGGGTACGGCCTT
GAGCCTCCTTATTCGAGCCGAACTAAGCCAGCCCGGAGCCCTTCTGGGCGATGACCAA
ATCTACAACGTTATCGTTACAGCTCATGCCTTCGTAATAATTTTCTTTATAGTAATACCAA
TCATGATTGGAGGATTCGGAAACTGGCTAGTCCCCCTGATAATTGGCGCACCAGACATG
GCCTTCCCTCGAATGAACAACATAAGCTTCTGACTTCTGCCCCCCTCGTTCCTACTACTA
TTAGCTTCTTCAGGAGTTGAAGCGGGAGCAGGCACCGGATGAACTGTCTACCCCCCAC
TAGCCGGAAACCTCGCCCATGCCGGAGCCTCCGTTGACTTAACCATTTTCTCCCTTCAT
CTGGCTGGTATTTCCTCCATCCTTGGGGCCATTAACTTCATCACAACTATTATTAACATG
AAGCCTCCTGCAATCTCACAATACCAGACCCCCCTTTTCGTGTGAGCAGTTCTCATTAC
AGCCGTACTCCTACTTCTCTCCCTTCCGGTTCTTGCTGCCGGCATCACAATACTTCTTAC
AGACCGGAACCTAAATACAACCTTCTTTGACCCGGCAGGCGGAGGAGACCCAAT

>Rhinogobius_sp4_SZ724
GACATTGGCACCCTTTATCTTGTATTTGGTGCCTGAGCTGGAATAGTGGGTACGGCCTT
GAGCCTCCTTATTCGAGCCGAACTAAGCCAGCCCGGAGCCCTTCTGGGCGATGACCAA
ATCTACAACGTTATCGTTACAGCTCATGCCTTCGTAATAATTTTCTTTATAGTAATACCAA
TCATGATTGGAGGATTCGGAAACTGGCTAGTCCCCCTGATAATTGGCGCACCAGACATG
GCCTTCCCTCGAATGAACAACATAAGCTTCTGACTTCTGCCCCCCTCGTTCCTACTACTA
TTAGCTTCTTCAGGAGTTGAAGCGGGAGCAGGCACCGGATGAACTGTCTACCCCCCAC
TAGCCGGAAACCTCGCCCATGCCGGAGCCTCCGTTGACTTAACCATTTTCTCCCTTCAT
CTGGCTGGTATTTCCTCCATCCTTGGGGCCATTAACTTCATCACAACTATTATTAACATG
AAGCCTCCTGCAATCTCACAATACCAGACCCCCCTTTTCGTGTGAGCAGTTCTCATTAC
AGCCGTACTCCTACTTCTCTCCCTTCCGGTTCTTGCTGCCGGCATCACAATACTTCTTAC
AGACCGGAACCTAAATACAACCTTCTTTGACCCGGCAGGCGGAGGAGACCCAAT
>Schistura_sp_SZ2755
GACATTGGCACCCTCTACTTAGTATTTGGTGCCTGAGCCGGAATAGTTGGTACTGCCCT
AAGCCTACTAATTCGAGCTGAGTTAAGCCAACCAGGATCTCTCCTCGGAGATGACCAA
ATTTACAATGTTATTGTTACCGCACACGCCTTTGTTATAATTTTCTTTATAGTAATACCAA
TTCTCATTGGAGGCTTTGGCAACTGACTCGTCCCCCTAATAATTGGGGCCCCTGACATA
GCATTTCCTCGAATAAATAATATAAGCTTCTGACTTCTACCCCCATCATTCCTTTTACTAT
TAGCCTCATCTGGCGTTGAAGCCGGGGCCGGGACAGGATGGACGGTTTATCCTCCACT
AGCAGGCAACTTAGCCCATGCAGGTGCATCCGTAGACCTAACCATTTTCTCCCTCCACC
TAGCGGGTGTGTCGTCTATTTTAGGGGCAATCAATTTCATTACAACAACAATTAATATAA
AACCCCCAGCTATTTCCCAATATCAAACCCCCTTGTTTGTATGAGCCGTGCTTGTAACTG
CTGTCCTTCTTCTCCTATCTCTACCAGTCCTAGCCGCCGGAATCACAATACTTCTAACTG
ATCGAAACTTAAATACCACATTCTTTGACCCGGCGGGAGGAGGAGACCCTAT
>Schistura_sp_SZ367
GACATTGGCACCCTCTACTTAGTATTTGGTGCCTGAGCCGGAATAGTTGGTACTGCCCT
AAGCCTACTAATTCGAGCTGAGTTAAGCCAACCAGGATCTCTCCTCGGAGATGACCAA
ATTTACAATGTTATTGTTACTGCACACGCCTTTGTTATAATTTTCTTTATAGTAATACCAAT
TCTCATTGGAGGCTTTGGCAACTGACTCGTCCCCCTAATAATCGGGGCCCCTGACATAG
CATTTCCTCGAATAAATAATATAAGCTTCTGACTTCTACCCCCATCATTCCTTTTACTATT
AGCCTCATCTGGTGTTGAAGCCGGGGCCGGGACAGGATGGACGGTTTATCCTCCACTA
GCAGGCAACTTAGCCCATGCAGGTGCATCCGTAGACCTAACCATTTTCTCCCTCCACCT
AGCGGGTGTGTCGTCTATTTTAGGAGCAATCAATTTCATTACAACAACAATTAATATAAA
ACCCCCAGCTATTTCCCAATATCAAACCCCCTTGTTTGTATGAGCCGTGCTTGTAACTGC
TGTCCTTCTTCTCCTATCTCTACCAGTCCTAGCCGCCGGAATCACAATACTTCTAACTGA
TCGAAACTTAAATACCACATTCTTTGACCCGGCGGGAGGAGGAGACCCTAT
>Sicyopterus_sp._SZ124
GACATTGGCACCCTCTATCTTGTATTTGGTGCATGGGCCGGGATAGTAGGAACTGCATT
AAGCCTACTAATCCGAGCAGAACTAAGCCAACCAGGCGCCCTCCTGGGAGATGACCA
GATTTATAATGTGATCGTAACAGCACATGCCTTTGTAATGATTTTCTTTATAGTCATACCA
ATTATAATCGGGGGCTTTGGAAACTGACTGATCCCCCTAATAATTGGTGCCCCCGACAT
GGCCTTCCCCCGAATAAATAACATAAGCTTTTGACTACTCCCGCCCTCATTCTTGTTACT
CCTAGCATCCTCAGGCGTAGAGGCTGGGGCCGGGACAGGATGAACAGTCTATCCCCCC
CTAGCAGGAAACCTCGCCCACGCCGGGGCATCCGTAGACTTAACAATCTTTTCCCTCCA

TTTAGCCGGAATTTCTTCTATCTTGGGAGCCATTAATTTATTACGACCATCCTTAATATA
AAACCTCCCGCCATCTCCCAGTATCAGACCCCTCTGTTTGTTTGAGCAGTACTAATTAC
AGCAGTCCTCCTTTTACTTTCCCTGCCTGTTCTAGCAGCCGGAATTACAATACTACTGAC
AGACCGAAACTTAAACACGACTTTCTTTGACCCCGCAGGAGGGGGAGACCCCAT
>Sicyopterus_sp._SZ210
GACATTGGCACCCTCTATCTTGTATTTGGTGCATGGGCCGGGATAGTAGGAACTGCATT
AAGCCTACTAATCCGAGCAGAACTAAGCCAACCAGGCGCCCTCCTGGGAGATGACCA
GATTTATAATGTGATCGTAACAGCACATGCCTTTGTAATGATTTTCTTTATAGTCATACCA
ATTATAATCGGGGGCTTTGGAAACTGACTGATCCCCCTAATAATTGGTGCCCCCGACAT
GGCCTTCCCCCGAATAAATAACATAAGCTTTTGACTACTCCCGCCCTCATTCTTGTTACT
CCTAGCATCCTCAGGCGTAGAGGCTGGGGCCGGGACAGGATGAACAGTCTATCCCCCC
CTAGCAGGAAACCTCGCCCACGCCGGGGCATCCGTAGACTTAACAATCTTTTCCCTCCA
TTTAGCCGGAATTTCTTCTATCTTGGGAGCCATTAATTTATTACGACCATCCTTAATATA
AAACCTCCCGCCATCTCCCAGTATCAGACCCCTCTGTTTGTTTGAGCAGTACTAATTAC
AGCAGTCCTCCTTTTACTTTCCCTGCCTGTTCTAGCAGCCGGAATTACAATACTACTGAC
AGACCGAAACTTAAACACGACTTTCTTTGACCCCGCAGGAGGGGGAGACCCCAT
>Siniperca_sp_SZ12
GACATTGGCACCCTCTATCTAGTATTTGGTGCCTGAGCCGGAATAGTGGGCACAGCCCT
AAGCCTGCTCATTCGAGCAGAACTAAGCCAACCAGGCGCCCTCCTAGGAGACGACCA
GATTTATAATGTAATTGTTACAGCACATGCATTCGTAATAATTTTCTTTATAGTAATGCCA
ATTATAATCGGAGGATTCGGAAACTGACTCGTACCACTAATAATTGGTGCCCCAGACAT
AGCATTCCCTCGAATGAATAACATGAGCTTCTGACTACTCCCACCGTCCTTCCTTCTCCT
TCTTGCCTCTTCCGGAGTAGAAGCTGGGGCCGGAACGGGATGAACCGTTTACCCACCC
CTGGCGGGTAACTTAGCCCATGCAGGGGCATCCGTCGACCTGACCATTTTTCTCTCCA
CTTAGCAGGAATTTCTTCCATCCTTGGAGCTATCAACTTCATTACAACTATTATTAACAT
GAAACCCCCTGCCATCTCCCAATACCAAACCCCTCTGTTCGTGTGGGCAGTCCTGATTA
CTGCTGTACTTCTTCTGCTCTCCCTACCCGTCCTGGCTGCTGGCATTACAATGCTTCTTA
CAGACCGAAACCTTAACACCACCTTCTTCGACCCCGCTGGAGGAGGTGACCCAAT
>Siniperca_sp_SZ126
GACATTGGCACCCTCTATCTAGTATTTGGTGCCTGAGCCGGAATAGTGGGCACAGCCCT
AAGCCTGCTCATTCGAGCAGAACTAAGCCAACCAGGCGCCCTCCTAGGAGACGACCA
GATTTATAATGTAATTGTTACAGCACATGCATTCGTAATAATTTTCTTTATAGTAATGCCA
ATTATAATCGGAGGATTCGGAAACTGACTCGTACCACTAATAATTGGTGCCCCAGACAT
AGCATTCCCTCGAATGAATAACATGAGCTTCTGACTACTCCCACCGTCCTTCCTTCTCCT
TCTTGCCTCTTCCGGAGTAGAAGCTGGGGCCGGAACGGGATGAACCGTTTACCCACCC
CTGGCGGGTAACTTAGCCCATGCAGGGGCATCCGTCGACCTGACCATTTTTCTCTCCA
CTTAGCAGGAATTTCTTCCATCCTTGGAGCTATCAACTTCATTACAACTATTATTAACAT
GAAACCCCCTGCCATCTCCCAATACCAAACCCCTCTGTTCGTGTGGGCAGTCCTGATTA
CTGCTGTACTTCTTCTGCTCTCCCTACCCGTCCTGGCTGCTGGCATTACAATGCTTCTTA
CAGACCGAAACCTTAACACCACCTTCTTCGACCCCGCTGGAGGAGGTGACCCAAT
>Siniperca_sp_SZ13
GACATTGGCACCCTCTATCTAGTATTTGGTGCCTGAGCCGGAATAGTGGGCACAGCCCT
AAGCCTGCTCATTCGAGCAGAACTAAGCCAACCAGGCGCCCTCCTAGGAGACGACCA
GATTTATAATGTAATTGTTACAGCACATGCATTCGTAATAATTTTCTTTATAGTAATGCCA

ATTATAATCGGAGGATTCGGAAACTGACTCGTACCACTAATAATTGGTGCCCCAGACAT
AGCATTCCCTCGAATGAATAACATGAGCTTCTGACTACTCCCACCGTCCTTCCTTCTCCT
TCTTGCCTCTTCCGGAGTAGAAGCTGGGGCCGGAACGGGATGAACCGTTTACCCACCC
CTGGCGGGTAACTTAGCCCATGCAGGGGCATCCGTCGACCTGACCATTTTTTCTCTCCA
CTTAGCAGGAATTTCTTCCATCCTTGGAGCTATCAACTTCATTACAACTATTATTAACAT
GAAACCCCCTGCCATCTCCCAATACCAAACCCCTCTGTTCGTGTGGGCAGTCCTGATTA
CTGCTGTACTTCTTCTGCTCTCCCTACCCGTCCTGGCTGCTGGCATTACAATGCTTCTTA
CAGACCGAAACCTTAACACCACCTTCTTCGACCCCGCTGGAGGAGGTGACCCAAT
>Siniperca_sp_SZ14
GACATTGGCACCCTCTATCTAGTATTTGGTGCCTGAGCCGGAATAGTGGGCACAGCCCT
AAGCCTGCTCATTCGAGCAGAACTAAGCCAACCAGGCGCCCTCCTAGGAGACGACCA
GATTTATAATGTAATTGTTACAGCACATGCATTCGTAATAATTTTCTTTATAGTAATGCCA
ATTATAATCGGAGGATTCGGAAACTGACTCGTACCACTAATAATTGGTGCCCCAGACAT
AGCATTCCCTCGAATGAATAACATGAGCTTCTGACTACTCCCACCGTCCTTCCTTCTCCT
TCTTGCCTCTTCCGGAGTAGAAGCTGGGGCCGGAACGGGATGAACCGTTTACCCACCC
CTGGCGGGTAACTTAGCCCATGCAGGGGCATCCGTCGACCTGACCATTTTTTCTCTCCA
CTTAGCAGGAATTTCTTCCATCCTTGGAGCTATCAACTTCATTACAACTATTATTAACAT
GAAACCCCCTGCCATCTCCCAATACCAAACCCCTCTGTTCGTATGGGCAGTCCTGATTA
CTGCTGTACTTCTTCTGCTCTCCCTACCCGTCCTGGCTGCTGGCATTACAATGCTTCTTA
CAGACCGAAACCTTAACACCACCTTCTTCGACCCCGCTGGAGGAGGTGACCCAAT
>Siniperca_sp_SZ186
GACATTGGCACCCTCTATCTAGTATTTGGTGCCTGAGCCGGAATAGTGGGCACAGCCCT
AAGCCTGCTCATTCGAGCAGAACTAAGCCAACCAGGCGCCCTCCTAGGAGACGACCA
GATTTATAATGTAATTGTTACAGCACATGCATTCGTAATAATTTTCTTTATAGTAATGCCA
ATTATAATCGGAGGATTCGGAAACTGACTCGTACCACTAATAATTGGTGCCCCAGACAT
AGCATTCCCTCGAATGAATAACATGAGCTTCTGACTACTCCCACCGTCCTTCCTTCTCCT
TCTTGCCTCTTCCGGAGTAGAAGCTGGGGCCGGAACGGGATGAACCGTTTACCCACCC
CTGGCGGGTAACTTAGCCCATGCAGGGGCATCCATCGACCTGACCATTTTTTCTCTCCA
CTTAGCAGGAATTTCTTCCATCCTTGGAGCTATCAACTTCATTACAACTATTATTAACAT
GAAACCCCCTGCCATCTCCCAATACCAAACCCCTCTGTTCGTGTGGGCAGTCCTGATTA
CTGCTGTACTTCTTCTGCTCTCCCTACCCGTCCTGGCTGCTGGCATTACAATGCTTCTTA
CAGACCGAAACCTTAACACCACCTTCTTCGACCCCGCTGGAGGAGGTGACCCAAT
>Siniperca_sp_SZ1992
GACATTGGCACCCTCTATCTAGTATTTGGTGCTTGAGCCGGAATAGTGGGCACAGCCCT
AAGCCTGCTCATTCGAGCAGAACTAAGCCAACCAGGCGCCCTCCTAGGAGACGACCA
GATTTATAATGTAATTGTTACAGCACATGCATTCGTAATAATTTTCTTTATAGTAATGCCA
ATTATAATCGGAGGATTCGGAAACTGACTCGTACCACTAATAATTGGTGCCCCAGACAT
AGCATTCCCTCGAATGAATAACATGAGCTTCTGACTACTCCCACCGTCCTTCCTTCTCCT
TCTTGCCTCTTCCGGAGTAGAAGCTGGGGCCGGAACGGGATGAACCGTTTACCCACCC
CTGGCGGGTAACTTAGCCCATGCAGGGGCATCCGTCGACCTGACCATTTTTTCTCTCCA
CTTAGCAGGAATTTCTTCCATCCTTGGAGCTATCAACTTCATTACAACTATTATTAACAT
GAAACCCCCTGCCATCTCCCAATACCAAACCCCTCTGTTCGTGTGGGCAGTCCTGATTA
CTGCTGTACTTCTTCTGCTCTCCCTACCCGTCCTGGCTGCTGGCATTACAATGCTTCTTA
CAGACCGAAACCTTAACACCACCTTCTTCGACCCCGCTGGAGGAGGTGACCCAAT

>Siniperca_sp_SZ203
GACATTGGCACCCTCTATCTAGTATTTGGTGCCTGAGCCGGAATAGTGGGCACAGCCCT
AAGCCTGCTCATTCGAGCAGAACTAAGCCAACCAGGCGCCCTCCTAGGAGACGACCA
GATTTATAATGTAATTGTTACAGCACATGCATTCGTAATAATTTTCTTTATAGTAATGCCA
ATTATAATCGGAGGATTCGGAAACTGACTCGTACCACTAATAATTGGTGCCCCAGACAT
AGCATTCCCTCGAATGAATAACATGAGCTTCTGACTACTCCCACCGTCCTTCCTTCTCCT
TCTTGCCTCTTCCGGAGTAGAAGCTGGGGCCGGAACGGGATGAACCGTTTACCCACCC
CTGGCGGGTAACTTAGCCCATGCAGGGGCATCCGTCGACCTGACCATTTTTTCTCTCCA
CTTAGCAGGAATTTCTTCCATCCTTGGAGCTATCAACTTCATTACAACTATTATTAACAT
GAAACCCCCTGCCATCTCTCAATACCAAACCCCTCTGTTCGTGTGGGCAGTCCTGATTA
CTGCTGTACTTCTTCTGCTCTCCCTACCCGTCCTGGCTGCTGGCATTACAATGCTTCTTA
CAGACCGAAACCTTAACACCACCTTCTTCGACCCCGCTGGAGGAGGTGACCCAAT
>Siniperca_sp_SZ21
GACATTGGCACCCTCTATCTAGTATTTGGTGCCTGAGCCGGAATAGTGGGCACAGCCCT
AAGCCTGCTCATTCGAGCAGAACTAAGCCAACCAGGCGCCCTCCTAGGAGACGACCA
GATTTATAATGTAATTGTTACAGCACATGCATTCGTAATAATTTTCTTTATAGTAATGCCA
ATTATAATCGGAGGATTCGGAAACTGACTCGTACCACTAATAATTGGTGCCCCAGACAT
AGCATTCCCTCGAATGAATAACATGAGCTTCTGACTACTCCCACCGTCCTTCCTTCTCCT
TCTTGCCTCTTCCGGAGTAGAAGCTGGGGCCGGAACGGGATGAACCGTTTACCCACCC
CTGGCGGGTAACTTAGCCCATGCAGGGGCATCCGTCGACCTGACCATTTTTTCTCTCCA
CTTAGCAGGAATTTCTTCCATCCTTGGAGCTATCAACTTCATTACAACTATTATTAACAT
GAAACCCCCTGCCATCTCCCAATACCAAACCCCTCTGTTCGTGTGGGCAGTCCTGATTA
CTGCTGTACTTCTTCTGCTCTCCCTACCCGTCCTGGCTGCTGGCATTACAATGCTTCTTA
CAGACCGAAACCTTAACACCACCTTCTTCGACCCCGCTGGAGGAGGTGACCCAAT
>Siniperca_sp_SZ22
GACATTGGCACCCTCTATCTAGTATTTGGTGCCTGAGCCGGAATAGTGGGCACAGCCCT
AAGCCTGCTCATTCGAGCAGAACTAAGCCAACCAGGCGCCCTCCTAGGAGACGACCA
GATTTATAATGTAATTGTTACAGCACATGCATTCGTAATAATTTTCTTTATAGTAATGCCA
ATTATAATCGGAGGATTCGGAAACTGACTCGTACCACTAATAATTGGTGCCCCAGACAT
AGCATTCCCTCGAATGAATAACATGAGCTTCTGACTACTCCCACCGTCCTTCCTTCTCCT
TCTTGCCTCTTCCGGAGTAGAAGCTGGGGCCGGAACGGGATGAACCGTTTACCCACCC
CTGGCGGGTAACTTAGCCCATGCAGGGGCATCCGTCGACCTGACCATTTTTTCTCTCCA
CTTAGCAGGAATTTCTTCCATCCTTGGAGCTATCAACTTCATTACAACTATTATTAACATA
AAACCCCCTGCCATCTCCCAATACCAAACCCCTCTGTTCGTGTGGGCAGTCCTGATTAC
TGCTGTACTTCTTCTGCTCTCCCTACCCGTCCTGGCTGCTGGCATTACAATGCTTCTTAC
AGACCGAAACCTTAACACCACCTTCTTCGACCCCGCTGGAGGAGGTGACCCAAT
>Siniperca_sp_SZ225
GACATTGGCACCCTCTATCTAGTATTTGGTGCCTGAGCCGGAATAGTGGGCACAGCCCT
AAGCCTGCTCATTCGAGCAGAACTAAGCCAACCAGGCGCCCTCCTAGGAGACGACCA
GATTTATAATGTAATTGTTACAGCACATGCATTCGTAATAATTTTCTTTATAGTAATGCCA
ATTATAATCGGAGGATTCGGAAACTGACTCGTACCACTAATAATTGGTGCCCCAGACAT
AGCATTCCCTCGAATGAATAACATGAGCTTCTGACTACTCCCACCGTCCTTCCTTCTCCT
TCTTGCCTCTTCCGGAGTAGAAGCTGGGGCCGGAACGGGATGAACCGTTTACCCACCC
CTGGCGGGTAACTTAGCCCATGCAGGGGCATCCGTCGACCTGACCATTTTTTCTCTCCA

CTTAGCAGGAATTTCTTCCATCCTTGGAGCTATCAACTTCATTACAACTATTATTAACAT
GAAACCCCCTGCCATCTCCCAATACCAAACCCCTCTGTTCGTGTGGGCAGTCCTGATTA
CTGCTGTACTTCTTCTGCTCTCCCTACCCGTCCTGGCTGCTGGCATTACAATGCTTCTTA
CAGACCGAAACCTTAACACCACCTTCTTCGACCCCGCTGGAGGAGGTGACCCAAT
>Siniperca_sp_SZ226
GACATTGGCACCCTCTATCTAGTATTTGGTGCCTGAGCCGGAATAGTGGGCACAGCCCT
AAGCCTGCTCATTCGAGCAGAACTAAGCCAACCAGGCGCCCTCCTAGGAGACGACCA
GATTTATAATGTAATTGTTACAGCACATGCATTCGTAATAATTTTCTTTATAGTAATGCCA
ATTATAATCGGAGGATTCGGAAACTGACTCGTACCACTAATAATTGGTGCCCCAGACAT
AGCATTCCCTCGAATGAATAACATGAGCTTCTGACTACTCCCACCGTCCTTCCTTCTCCT
TCTTGCCTCTTCCGGAGTAGAAGCTGGGGCCGGAACGGGATGAACCGTTTACCCACCC
CTGGCGGGTAACTTAGCCCATGCAGGGGCATCCGTCGACCTGACCATTTTTTCTCTCCA
CTTAGCAGGAATTTCTTCCATCCTTGGAGCTATCAACTTCATTACAACTATTATTAACAT
GAAACCCCCTGCCATCTCCCAATACCAAACCCCTCTGTTCGTGTGGGCAGTCCTGATTA
CTGCTGTACTTCTTCTGCTCTCCCTACCCGTCCTGGCTGCTGGCATTACAATGCTTCTTA
CAGACCGAAACCTTAACACCACCTTCTTCGACCCCGCTGGAGGAGGTGACCCAAT
>Siniperca_sp_SZ233
GACATTGGCACCCTCTATCTAGTATTTGGTGCCTGAGCCGGAATAGTGGGCACAGCCCT
AAGCCTGCTCATTCGAGCAGAACTAAGCCAACCAGGCGCCCTCCTAGGAGACGACCA
GATTTATAATGTAATTGTTACAGCACATGCATTCGTAATAATTTTCTTTATAGTAATGCCA
ATTATAATCGGAGGATTCGGAAACTGACTCGTACCACTAATAATTGGTGCCCCAGACAT
AGCATTCCCTCGAATGAATAACATGAGCTTCTGACTACTCCCACCGTCCTTCCTTCTCCT
TCTTGCCTCTTCCGGAGTAGAAGCTGGGGCCGGAACGGGATGAACCGTTTACCCACCC
CTGGCGGGTAACTTAGCCCATGCAGGGGCATCCGTCGACCTGACCATTTTTTCTCTCCA
CTTAGCAGGAATTTCTTCCATCCTTGGAGCTATCAACTTCATTACAACTATTATTAACAT
GAAACCCCCTGCCATCTCCCAATACCAAACCCCTCTGTTCGTGTGGGCAGTCCTGATTA
CTGCTGTACTTCTTCTGCTCTCCCTACCCGTCCTGGCTGCTGGCATTACAATGCTTCTTA
CAGACCGAAACCTTAACACCACCTTCTTCGACCCCGCTGGAGGAGGTGACCCAAT
>Siniperca_sp_SZ254
GACATTGGCACCCTCTATCTAGTATTTGGTGCCTGAGCCGGAATAGTGGGCACAGCCCT
AAGCCTGCTCATTCGAGCAGAACTAAGCCAACCAGGCGCCCTCCTAGGAGACGACCA
GATTTATAATGTAATTGTTACAGCACATGCATTCGTAATAATTTTCTTTATAGTAATGCCA
ATTATAATCGGAGGATTCGGAAACTGACTCGTACCACTAATAATTGGTGCCCCAGACAT
AGCATTCCCTCGAATGAATAACATGAGCTTCTGACTACTCCCACCGTCCTTCCTTCTCCT
TCTTGCCTCTTCCGGAGTAGAAGCTGGGGCCGGAACGGGATGAACCGTTTACCCACCC
CTGGCGGGTAACTTAGCCCATGCAGGGGCATCCGTCGACCTGACCATTTTTTCTCTCCA
CTTAGCAGGAATTTCTTCCATCCTTGGAGCTATCAACTTCATTACAACTATTATTAACAT
GAAACCCCCTGCCATCTCCCAATACCAAACCCCTCTGTTCGTGTGGGCAGTCCTGATTA
CTGCTGTACTTCTTCTGCTCTCCCTACCCGTCCTGGCTGCTGGCATTACAATGCTTCTTA
CAGACCGAAACCTTAACACCACCTTCTTCGACCCCGCTGGAGGAGGTGACCCAAT
>Siniperca_sp_SZ256
GACATTGGCACCCTCTATCTAGTATTTGGTGCCTGAGCCGGAATAGTGGGCACAGCCCT
AAGCCTGCTCATTCGAGCAGAACTAAGCCAACCAGGCGCCCTCCTAGGAGACGACCA
GATTTATAATGTAATTGTTACAGCACATGCATTCGTAATAATTTTCTTTATAGTAATGCCA

ATTATAATCGGAGGATTCGGAAACTGACTCGTACCACTAATAATTGGTGCCCCAGACAT
AGCATTCCCTCGAATGAATAACATGAGCTTCTGACTACTCCCACCGTCCTTCCTTCTCCT
TCTTGCCTCTTCCGGAGTAGAAGCTGGGGCCGGAACGGGATGAACCGTTTACCCACCC
CTGGCGGGTAACTTAGCCCATGCAGGGGCATCCGTCGACCTGACCATTTTTTCTCTCCA
CTTAGCAGGAATTTCTTCCATCCTTGGAGCTATCAACTTCATTACAACTATTATTAACATA
AAACCCCCTGCCATCTCCCAATACCAAACCCCTCTGTTCGTGTGGGCAGTCCTGATTAC
TGCTGTACTTCTTCTGCTCTCCCTACCCGTCCTGGCTGCTGGCATTACAATGCTTCTTAC
AGACCGAAACCTTAACACCACCTTCTTCGACCCCGCTGGAGGAGGTGACCCAAT
>Siniperca_sp_SZ26
GACATTGGCACCCTCTATCTAGTATTTGGTGCCTGAGCCGGAATAGTGGGCACAGCCCT
AAGCCTGCTCATTCGAGCAGAACTAAGCCAACCAGGCGCCCTCCTAGGAGACGACCA
GATTTATAATGTAATTGTTACAGCACATGCATTCGTAATAATTTTCTTTATAGTAATGCCA
ATTATAATCGGAGGATTCGGAAACTGACTCGTACCACTAATAATTGGTGCCCCAGACAT
AGCATTCCCTCGAATGAATAACATGAGCTTCTGACTACTCCCACCGTCCTTCCTTCTCCT
TCTTGCCTCTTCCGGAGTAGAAGCTGGGGCCGGAACGGGATGAACCGTTTACCCACCC
CTGGCGGGTAACTTAGCCCATGCAGGGGCATCCGTCGACCTGACCATTTTTTCTCTCCA
CTTAGCAGGAATTTCTTCCATCCTTGGAGCTATCAACTTCATTACAACTATTATTAACAT
GAAACCCCCTGCCATCTCCCAATACCAAACCCCTCTGTTCGTGTGGGCAGTCCTGATTA
CTGCTGTACTTCTTCTGCTCTCCCTACCCGTCCTGGCTGCTGGCATTACAATGCTTCTTA
CAGACCGAAACCTTAACACCACCTTCTTCGACCCCGCTGGAGGAGGTGACCCAAT
>Siniperca_sp_SZ266
GACATTGGCACCCTCTATCTAGTATTTGGTGCCTGAGCCGGAATAGTGGGCACAGCCCT
AAGCCTGCTCATTCGAGCAGAACTAAGCCAACCAGGCGCCCTCCTAGGAGACGACCA
GATTTATAATGTAATTGTTACAGCACATGCATTCGTAATAATTTTCTTTATAGTAATGCCA
ATTATAATCGGAGGATTCGGAAACTGACTCGTACCACTAATAATTGGTGCCCCAGACAT
AGCATTCCCTCGAATGAATAACATGAGCTTCTGACTACTCCCACCGTCCTTCCTTCTCCT
TCTTGCCTCTTCCGGAGTAGAAGCTGGGGCCGGAACGGGATGAACCGTTTACCCACCC
CTGGCGGGTAACTTAGCCCATGCAGGGGCATCCGTCGACCTGACCATTTTTTCTCTCCA
CTTAGCAGGAATTTCTTCCATCCTTGGAGCTATCAACTTCATTACAACTATTATTAACAT
GAAACCCCCTGCCATCTCCCAATACCAAACCCCTCTGTTCGTGTGGGCAGTCCTGATTA
CTGCTGTACTTCTTCTGCTCTCCCTACCCGTCCTGGCTGCTGGCATTACAATGCTTCTTA
CAGACCGAAACCTTAACACCACCTTCTTCGACCCCGCTGGAGGAGGTGACCCAAT
>Siniperca_sp_SZ2675
GACATTGGCACCCTCTATCTAGTATTTGGTGCCTGAGCCGGAATAGTGGGCACAGCCCT
AAGCCTGCTCATTCGAGCAGAACTAAGCCAACCAGGCGCCCTCCTAGGAGACGACCA
GATTTATAATGTAATTGTTACAGCACATGCATTCGTAATAATTTTCTTTATAGTAATGCCA
ATTATAATCGGAGGATTCGGAAACTGACTCGTACCACTAATAATTGGTGCCCCAGACAT
AGCATTCCCTCGAATGAATAACATGAGCTTCTGACTACTCCCACCGTCCTTCCTTCTCCT
TCTTGCCTCTTCCGGAGTAGAAGCTGGGGCCGGAACGGGATGAACCGTTTACCCACCC
CTGGCGGGTAACTTAGCCCATGCAGGGGCATCCGTCGACCTGACCATTTTTTCTCTCCA
CTTAGCAGGAATTTCTTCCATCCTTGGAGCTATCAACTTCATTACAACTATTATTAACAT
GAAACCCCCTGCCATCTCCCAATACCAAACCCCTCTGTTCGTGTGGGCAGTCCTGATTA
CTGCTGTACTTCTTCTGCTCTCCCTACCCGTCCTGGCTGCTGGCATTACAATGCTTCTTA
CAGACCGAAACCTTAACACCACCTTCTTCGACCCCGCTGGAGGAGGTGACCCAAT

>Siniperca_sp_SZ2686
GACATTGGCACCCTCTATCTAGTATTTGGTGCCTGAGCCGGAATAGTGGGCACAGCCCT
AAGCCTGCTCATTCGAGCAGAACTAAGCCAACCAGGCGCCCTCCTAGGAGACGACCA
GATTTATAATGTAATTGTTACAGCACATGCATTCGTAATAATTTTCTTTATAGTAATGCCA
ATTATAATCGGAGGATTCGGAAACTGACTCGTACCACTAATAATTGGTGCCCCAGACAT
AGCATTCCCTCGAATGAATAACATGAGCTTCTGACTACTCCCACCGTCCTTCCTTCTCCT
TCTTGCCTCTTCCGGAGTAGAAGCTGGGGCCGGAACGGGATGAACCGTTTACCCACCC
CTGGCGGGTAACTTAGCCCATGCAGGGGCATCCGTCGACCTGACCATTTTTCTCTCCA
CTTAGCAGGAATTTCTTCCATCCTTGGAGCTATCAACTTCATTACAACTATTATTAACAT
GAAACCCCCTGCCATCTCCCAATACCAAACCCCTCTGTTCGTGTGGGCAGTCCTGATTA
CTGCTGTACTTCTTCTGCTCTCCCTACCCGTCCTGGCTGCTGGCATTACAATGCTTCTTA
CAGACCGAAACCTTAACACCACCTTCTTCGACCCCGCTGGAGGAGGTGACCCAAT
>Siniperca_sp_SZ2687
GACATTGGCACCCTCTATCTAGTATTTGGTGCCTGAGCCGGAATAGTGGGCACAGCCCT
AAGCCTGCTCATTCGAGCAGAACTAAGCCAACCAGGCGCCCTCCTAGGAGACGACCA
GATTTATAATGTAATTGTTACAGCACATGCATTCGTAATAATTTTCTTTATAGTAATGCCA
ATTATAATCGGAGGATTCGGAAACTGACTCGTACCACTAATAATTGGTGCCCCAGACAT
AGCATTCCCTCGAATGAATAACATGAGCTTCTGACTACTCCCACCGTCCTTCCTTCTCCT
TCTTGCCTCTTCCGGAGTAGAAGCTGGGGCCGGAACGGGATGAACCGTTTACCCACCC
CTGGCGGGTAACTTAGCCCATGCAGGGGCATCCGTCGACCTGACCATTTTTCTCTCCA
CTTAGCAGGAATTTCTTCCATCCTTGGAGCTATCAACTTCATTACAACTATTATTAACATA
AAACCCCCTGCCATCTCCCAATACCAAACCCCTCTGTTCGTGTGGGCAGTCCTGATTAC
TGCTGTACTTCTTCTGCTCTCCCTACCCGTCCTGGCTGCTGGCATTACAATGCTTCTTAC
AGACCGAAACCTTAACACCACCTTCTTCGACCCCGCTGGAGGAGGTGACCCAAT
>Siniperca_sp_SZ2695
GACATTGGCACCCTCTATCTAGTATTTGGTGCCTGAGCCGGAATAGTGGGCACAGCCCT
AAGCCTGCTCATTCGAGCAGAACTAAGCCAACCAGGCGCCCTCCTAGGAGACGACCA
GATTTATAATGTAATTGTTACAGCACATGCATTCGTAATAATTTTCTTTATAGTAATGCCA
ATTATAATCGGAGGATTCGGAAACTGACTCGTACCACTAATAATTGGTGCCCCAGACAT
AGCATTCCCTCGAATGAATAACATGAGCTTCTGACTACTCCCACCGTCCTTCCTTCTCCT
TCTTGCCTCTTCCGGAGTAGAAGCTGGGGCCGGAACGGGATGAACCGTTTACCCACCC
CTGGCGGGTAACTTAGCCCATGCAGGGGCATCCGTCGACCTGACCATTTTTCTCTCCA
CTTAGCAGGAATTTCTTCCATCCTTGGAGCTATCAACTTCATTACAACTATTATTAACAT
GAAACCCCCTGCCATCTCTCAATACCAAACCCCTCTGTTCGTGTGGGCAGTCCTGATTA
CTGCTGTACTTCTTCTGCTCTCCCTACCCGTCCTGGCTGCTGGCATTACAATGCTTCTTA
CAGACCGAAACCTTAACACCACCTTCTTCGACCCCGCTGGAGGAGGTGACCCAAT
>Siniperca_sp_SZ27
GACATTGGCACCCTCTATCTAGTATTTGGTGCCTGAGCCGGAATAGTGGGCACAGCCCT
AAGCCTGCTCATTCGAGCAGAACTAAGCCAACCAGGCGCCCTCCTAGGAGACGACCA
GATTTATAATGTAATTGTTACAGCACATGCATTCGTAATAATTTTCTTTATAGTAATGCCA
ATTATAATCGGAGGATTCGGAAACTGACTCGTACCACTAATAATTGGTGCCCCAGACAT
AGCATTCCCTCGAATGAATAACATGAGCTTCTGACTACTCCCACCGTCCTTCCTTCTCCT
TCTTGCCTCTTCCGGAGTAGAAGCTGGGGCCGGAACGGGATGAACCGTTTACCCACCC
CTGGCGGGTAACTTAGCCCATGCAGGGGCATCCGTCGACCTGACCATTTTTCTCTCCA

CTTAGCAGGAATTTCTTCCATCCTTGGAGCTATCAACTTCATTACAACTATTATTAACAT
GAAACCCCCTGCCATCTCCCAATACCAAACCCCTCTGTTCGTGTGGGCAGTCCTGATTA
CTGCTGTACTTCTTCTGCTCTCCCTACCCGTCCTGGCTGCTGGCATTACAATGCTTCTTA
CAGACCGAAACCTTAACACCACCTTCTTCGACCCCGCTGGAGGAGGTGACCCAAT
>Siniperca_sp_SZ2701
GACATTGGCACCCTCTATCTAGTATTTGGTGCCTGAGCCGGAATAGTGGGCACAGCCCT
AAGCCTGCTCATTCGAGCAGAACTAAGCCAACCAGGCGCCCTCCTAGGAGACGACCA
GATTTATAATGTAATTGTTACAGCACATGCATTCGTAATAATTTTCTTTATAGTAATGCCA
ATTATAATCGGAGGATTCGGAAACTGACTCGTACCACTAATAATTGGTGCCCCAGACAT
AGCATTCCCTCGAATGAATAACATGAGCTTCTGACTACTCCCACCGTCCTTCCTTCTCCT
TCTTGCCTCTTCCGGAGTAGAAGCTGGGGCCGGAACGGGATGAACCGTTTACCCACCC
CTGGCGGGTAACTTAGCCCATGCAGGGGCATCCGTCGACCTGACCATTTTTTCTCTCCA
CTTAGCAGGAATTTCTTCCATCCTTGGAGCTATCAACTTCATTACAACTATTATTAACAT
GAAACCCCCTGCCATCTCCCAATACCAAACCCCTCTGTTCGTGTGGGCAGTCCTGATTA
CTGCTGTACTTCTTCTGCTCTCCCTACCCGTCCTGGCTGCTGGCATTACAATGCTTCTTA
CAGACCGAAACCTTAACACCACCTTCTTCGACCCCGCTGGAGGAGGTGACCCAAT
>Siniperca_sp_SZ2738
GACATTGGCACCCTCTATCTAGTATTTGGTGCCTGAGCCGGAATAGTGGGCACAGCCCT
AAGCCTGCTCATTCGAGCAGAACTAAGCCAACCAGGCGCCCTCCTAGGAGACGACCA
GATTTATAATGTAATTGTTACAGCACATGCATTCGTAATAATTTTCTTTATAGTAATGCCA
ATTATAATCGGAGGATTCGGAAACTGACTCGTACCACTAATAATTGGTGCCCCAGACAT
AGCATTCCCTCGAATGAATAACATGAGCTTCTGACTACTCCCACCGTCCTTCCTTCTCCT
TCTTGCCTCTTCCGGAGTAGAAGCTGGGGCCGGAACGGGATGAACCGTTTACCCACCC
CTGGCGGGTAACTTAGCCCATGCAGGGGCATCCGTCGACCTGACCATTTTTTCTCTCCA
CTTAGCAGGAATTTCTTCCATCCTTGGAGCTATCAACTTCATTACAACTATTATTAACAT
GAAACCCCCTGCCATCTCCCAATACCAAACCCCTCTGTTCGTGTGGGCAGTCCTGATTA
CTGCTGTACTTCTTCTGCTCTCCCTACCCGTCCTGGCTGCTGGCATTACAATGCTTCTTA
CAGACCGAAACCTTAACACCACCTTCTTCGACCCCGCTGGAGGAGGTGACCCAAT
>Siniperca_sp_SZ2741
GACATTGGCACCCTCTATCTAGTATTTGGTGCCTGAGCCGGAATAGTGGGCACAGCCCT
AAGCCTGCTCATTCGAGCAGAACTAAGCCAACCAGGCGCCCTCCTAGGAGACGACCA
GATTTATAATGTAATTGTTACAGCACATGCATTCGTAATAATTTTCTTTATAGTAATGCCA
ATTATAATCGGAGGATTCGGAAACTGACTCGTACCACTAATAATTGGTGCCCCAGACAT
AGCATTCCCTCGAATGAATAACATGAGCTTCTGACTACTCCCACCGTCCTTCCTTCTCCT
TCTTGCCTCTTCCGGAGTAGAAGCTGGGGCCGGAACGGGATGAACCGTTTACCCACCC
CTGGCGGGTAACTTAGCCCATGCAGGGGCATCCGTCGACCTGACCATTTTTTCTCTCCA
CTTAGCAGGAATTTCTTCCATCCTTGGAGCTATCAACTTCATTACAACTATTATTAACAT
GAAACCCCCTGCCATCTCCCAATACCAAACCCCTCTGTTCGTGTGGGCAGTCCTGATTA
CTGCTGTACTTCTTCTGCTCTCCCTACCCGTCCTGGCTGCTGGCATTACAATGCTTCTTA
CAGACCGAAACCTTAACACCACCTTCTTCGACCCCGCTGGAGGAGGTGACCCAAT
>Siniperca_sp_SZ2750
GACATTGGCACCCTCTATCTAGTATTTGGTGCCTGAGCCGGAATAGTGGGCACAGCCCT
AAGCCTGCTCATTCGAGCAGAACTAAGCCAACCAGGCGCCCTCCTAGGAGACGACCA
GATTTATAATGTAATTGTTACAGCACATGCATTCGTAATAATTTTCTTTATAGTAATGCCA

ATTATAATCGGAGGATTCGGAAACTGACTCGTACCACTAATAATTGGTGCCCCAGACAT
AGCATTCCCTCGAATGAATAACATGAGCTTCTGACTACTCCCACCGTCCTTCCTTCTCCT
TCTTGCCTCTTCCGGAGTAGAAGCTGGGGCCGGAACGGGATGAACCGTTTACCCACCC
CTGGCGGGTAACTTAGCCCATGCAGGGGCATCCGTCGACCTGACCATTTTTCTCTCCA
CTTAGCAGGAATTTCTTCCATCCTTGGAGCTATCAACTTCATTACAACTATTATTAACAT
GAAACCCCCTGCCATCTCCCAATACCAAACCCCTCTGTTCGTGTGGGCAGTCCTGATTA
CTGCTGTACTTCTTCTGCTCTCCCTACCCGTCCTGGCTGCTGGCATTACAATGCTTCTTA
CAGACCGAAACCTTAACACCACCTTCTTCGACCCCGCTGGAGGAGGTGACCCAAT
>Siniperca_sp_SZ276
GACATTGGCACCCTCTATCTAGTATTTGGTGCCTGAGCCGGAATAGTGGGCACAGCCCT
AAGCCTGCTCATTCGAGCAGAACTAAGCCAACCAGGCGCCCTCCTAGGAGACGACCA
GATTTATAATGTAATTGTTACAGCACATGCATTCGTAATAATTTTCTTTATAGTAATGCCA
ATTATAATCGGAGGATTCGGAAACTGACTCGTACCACTAATAATTGGTGCCCCAGACAT
AGCATTCCCTCGAATGAATAACATGAGCTTCTGACTACTCCCACCGTCCTTCCTTCTCCT
TCTTGCCTCTTCCGGAGTAGAAGCTGGGGCCGGAACGGGATGAACCGTTTACCCACCC
CTGGCGGGTAACTTAGCCCATGCAGGGGCATCCGTCGACCTGACCATTTTTCTCTCCA
CTTAGCAGGAATTTCTTCCATCCTTGGAGCTATCAACTTCATTACAACTATTATTAACAT
GAAACCCCCTGCCATCTCCCAATACCAAACCCCTCTGTTCGTGTGGGCAGTCCTGATTA
CTGCTGTACTTCTTCTGCTCTCCCTACCCGTCCTGGCTGCTGGCATTACAATGCTTCTTA
CAGACCGAAACCTTAACACCACCTTCTTCGACCCCGCTGGAGGAGGTGACCCAAT
>Siniperca_sp_SZ2760
GACATTGGCACCCTCTATCTAGTATTTGGTGCCTGAGCCGGAATAGTGGGCACAGCCCT
AAGCCTGCTCATTCGAGCAGAACTAAGCCAACCAGGCGCCCTCCTAGGAGACGACCA
GATTTATAATGTAATTGTTACAGCACATGCATTCGTAATAATTTTCTTTATAGTAATGCCA
ATTATAATCGGAGGATTCGGAAACTGACTCGTACCACTAATAATTGGTGCCCCAGACAT
AGCATTCCCTCGAATGAATAACATGAGCTTCTGACTACTCCCACCGTCCTTCCTTCTCCT
TCTTGCCTCTTCCGGAGTAGAAGCTGGGGCCGGAACGGGATGAACCGTTTACCCACCC
CTGGCGGGTAACTTAGCCCATGCAGGGGCATCCGTCGACCTGACCATTTTTCTCTCCA
CTTAGCAGGAATTTCTTCCATCCTTGGAGCTATCAACTTCATTACAACTATTATTAACAT
GAAACCCCCTGCCATCTCCCAATACCAAACCCCTCTGTTCGTGTGGGCAGTCCTGATTA
CTGCTGTACTTCTTCTGCTCTCCCTACCCGTCCTGGCTGCTGGCATTACAATGCTTCTTA
CAGACCGAAACCTTAACACCACCTTCTTCGACCCCGCTGGAGGAGGTGACCCAAT
>Siniperca_sp_SZ2769
GACATTGGCACCCTCTATCTAGTATTTGGTGCCTGAGCCGGAATAGTGGGCACAGCCCT
AAGCCTGCTCATTCGAGCAGAACTAAGCCAACCAGGCGCCCTCCTAGGAGACGACCA
GATTTATAATGTAATTGTTACAGCACATGCATTCGTAATAATTTTCTTTATAGTAATGCCA
ATTATAATCGGAGGATTCGGAAACTGACTCGTACCACTAATAATTGGTGCCCCAGACAT
AGCATTCCCTCGAATGAATAACATGAGCTTCTGACTACTCCCACCGTCCTTCCTTCTCCT
TCTTGCCTCTTCCGGAGTAGAAGCTGGGGCCGGAACGGGATGAACCGTTTACCCACCC
CTGGCGGGTAACTTAGCCCATGCAGGGGCATCCGTCGACCTGACCATTTTTCTCTCCA
CTTAGCAGGAATTTCTTCCATCCTTGGAGCTATCAACTTCATTACAACTATTATTAACAT
GAAACCCCCTGCCATCTCCCAATACCAAACCCCTCTGTTCGTGTGGGCAGTCCTGATTA
CTGCTGTACTTCTTCTGCTCTCCCTACCCGTCCTGGCTGCTGGCATTACAATGCTTCTTA
CAGACCGAAACCTTAACACCACCTTCTTCGACCCCGCTGGAGGAGGTGACCCAAT

>Siniperca_sp_SZ2812
GACATTGGCACCCTCTATCTAGTATTTGGTGCCTGAGCCGGAATAGTGGGCACAGCCCT
AAGCCTGCTCATTCGAGCAGAACTAAGCCAACCAGGCGCCCTCCTAGGAGACGACCA
GATTTATAATGTAATTGTTACAGCACATGCATTCGTAATAATTTTCTTTATAGTAATGCCA
ATTATAATCGGAGGATTCGGAAACTGACTCGTACCACTAATAATTGGTGCCCCAGACAT
AGCATTCCCTCGAATGAATAACATGAGCTTCTGACTACTCCCACCGTCCTTCCTTCTCCT
TCTTGCCTCTTCCGGAGTAGAAGCTGGGGCCGGAACGGGATGAACCGTTTACCCACCC
CTGGCGGGTAACTTAGCCCATGCAGGGGCATCCGTCGACCTGACCATTTTTTCTCTCCA
CTTAGCAGGAATTTCTTCCATCCTTGGAGCTATCAACTTCATTACAACTATTATTAACAT
GAAACCCCCTGCCATCTCCCAATACCAAACCCCTCTGTTCGTGTGGGCAGTCCTGATTA
CTGCTGTACTTCTTCTGCTCTCCCTACCCGTCCTGGCTGCTGGCATTACAATGCTTCTTA
CAGACCGAAACCTTAACACCACCTTCTTCGACCCCGCTGGAGGAGGTGACCCAAT
>Siniperca_sp_SZ2841
GACATTGGCACCCTCTATCTAGTATTTGGTGCCTGAGCCGGAATAGTGGGCACAGCCCT
AAGCCTGCTCATTCGAGCAGAACTAAGCCAACCAGGCGCCCTCCTAGGAGACGACCA
GATTTATAATGTAATTGTTACAGCACATGCATTCGTAATAATTTTCTTTATAGTAATGCCA
ATTATAATCGGAGGATTCGGAAACTGACTCGTACCACTAATAATTGGTGCCCCAGACAT
AGCATTCCCTCGAATGAATAACATGAGCTTCTGACTACTCCCACCGTCCTTCCTTCTCCT
TCTTGCCTCTTCCGGAGTAGAAGCTGGGGCCGGAACGGGATGAACCGTTTACCCACCC
CTGGCGGGTAACTTAGCCCATGCAGGGGCATCCGTCGACCTGACCATTTTTTCTCTCCA
CTTAGCAGGAATTTCTTCCATCCTTGGAGCTATCAACTTCATTACAACTATTATTAACAT
GAAACCCCCTGCCATCTCCCAATACCAAACCCCTCTGTTCGTGTGGGCAGTCCTGATTA
CTGCTGTACTTCTTCTGCTCTCCCTACCCGTCCTGGCTGCTGGCATTACAATGCTTCTTA
CAGACCGAAACCTTAACACCACCTTCTTCGACCCCGCTGGAGGAGGTGACCCAAT
>Siniperca_sp_SZ2857
GACATTGGCACCCTCTATCTAGTATTTGGTGCCTGAGCCGGAATAGTGGGCACAGCCCT
AAGCCTGCTCATTCGAGCAGAACTAAGCCAACCAGGCGCCCTCCTAGGAGACGACCA
GATTTATAATGTAATTGTTACAGCACATGCATTCGTAATAATTTTCTTTATAGTAATGCCA
ATTATAATCGGAGGATTCGGAAACTGACTCGTACCACTAATAATTGGTGCCCCAGACAT
AGCATTCCCTCGAATGAATAACATGAGCTTCTGACTACTCCCACCGTCCTTCCTTCTCCT
TCTTGCCTCTTCCGGAGTAGAAGCTGGGGCCGGAACGGGATGAACCGTTTACCCACCC
CTGGCGGGTAACTTAGCCCATGCAGGGGCATCCGTCGACCTGACCATTTTTTCTCTCCA
CTTAGCAGGAATTTCTTCCATCCTTGGAGCTATCAACTTCATTACAACTATTATTAACAT
GAAACCCCCTGCCATCTCCCAATACCAAACCCCTCTGTTCGTGTGGGCAGTCCTGATTA
CTGCTGTACTTCTTCTGCTCTCCCTACCCGTCCTGGCTGCTGGCATTACAATGCTTCTTA
CAGACCGAAACCTTAACACCACCTTCTTCGACCCCGCTGGAGGAGGTGACCCAAT
>Siniperca_sp_SZ2943
GACATTGGCACCCTCTATCTAGTATTTGGTGCCTGAGCCGGAATAGTGGGCACAGCCCT
AAGCCTGCTCATTCGAGCAGAACTAAGCCAACCAGGCGCCCTCCTAGGAGACGACCA
GATTTATAATGTAATTGTTACAGCACATGCATTCGTAATAATTTTCTTTATAGTAATGCCA
ATTATAATCGGAGGATTCGGAAACTGACTCGTACCACTAATAATTGGTGCCCCAGACAT
AGCATTCCCTCGAATGAATAACATGAGCTTCTGACTACTCCCACCGTCCTTCCTTCTCCT
TCTTGCCTCTTCCGGAGTAGAAGCTGGGGCCGGAACGGGATGAACCGTTTACCCACCC
CTGGCGGGTAACTTAGCCCATGCAGGGGCATCCGTCGACCTGACCATTTTTTCTCTCCA

CTTAGCAGGAATTTCTTCCATCCTTGGAGCTATCAACTTCATTACAACTATTATTAACATA
AAACCCCCTGCCATCTCCCAATACCAAACCCCTCTGTTCGTGTGGGCAGTCCTGATTAC
TGCTGTACTTCTTCTGCTCTCCCTACCCGTCCTGGCTGCTGGCATTACAATGCTTCTTAC
AGACCGAAACCTTAACACCACCTTCTTCGACCCCGCTGGAGGAGGTGACCCAAT
>Siniperca_sp_SZ2953
GACATTGGCACCCTCTATCTAGTATTTGGTGCCTGAGCCGGAATAGTGGGCACAGCCCT
AAGCCTGCTCATTCGAGCAGAACTAAGCCAACCAGGCGCCCTCCTAGGAGACGACCA
GATTTATAATGTAATTGTTACAGCACATGCATTCGTAATAATTTTCTTTATAGTAATGCCA
ATTATAATCGGAGGATTCGGAAACTGACTCGTACCACTAATAATTGGTGCCCCAGACAT
AGCATTCCCTCGAATGAATAACATGAGCTTCTGACTACTCCCACCGTCCTTCCTTCTCCT
TCTTGCCTCTTCCGGAGTAGAAGCTGGGGCCGGAACGGGATGAACCGTTTACCCACCC
CTGGCGGGTAACTTAGCCCATGCAGGGGCATCCGTCGACCTGACCATTTTTCTCTCCA
CTTAGCAGGAATTTCTTCCATCCTTGGAGCTATCAACTTCATTACAACTATTATTAACAT
GAAACCCCCTGCCATCTCCCAATACCAAACCCCTCTGTTCGTGTGGGCAGTCCTGATTA
CTGCTGTACTTCTTCTGCTCTCCCTACCCGTCCTGGCTGCTGGCATTACAATGCTTCTTA
CAGACCGAAACCTTAACACCACCTTCTTCGACCCCGCTGGAGGAGGTGACCCAAT
>Siniperca_sp_SZ2967
GACATTGGCACCCTCTATCTAGTATTTGGTGCCTGAGCCGGAATAGTGGGCACAGCCCT
AAGCCTGCTCATTCGAGCAGAACTAAGCCAACCAGGCGCCCTCCTAGGAGACGACCA
GATTTATAATGTAATTGTTACAGCACATGCATTCGTAATAATTTTCTTTATAGTAGTGCCA
ATTATAATCGGAGGATTCGGAAACTGACTCGTACCACTAATAATTGGTGCCCCAGACAT
AGCATTCCCTCGAATGAATAACATGAGCTTCTGACTACTCCCACCGTCCTTCCTTCTCCT
TCTTGCCTCTTCCGGAGTAGAAGCTGGGGCCGGAACGGGATGAACCGTTTACCCACCC
CTGGCGGGTAACTTAGCCCATGCAGGGGCATCCGTCGACCTGACCATTTTTCTCTCCA
CTTAGCAGGAATTTCTTCCATCCTTGGAGCTATCAACTTCATTACAACTATTATTAACAT
GAAACCCCCTGCCATCTCCCAATACCAAACCCCTCTGTTCGTGTGGGCAGTCCTGATTA
CTGCTGTACTTCTTCTGCTCTCCCTACCCGTCCTGGCTGCTGGCATTACAATGCTTCTTA
CAGACCGAAACCTTAACACCACCTTCTTCGACCCCGCTGGAGGAGGTGACCCAAT
>Siniperca_sp_SZ2968
GACATTGGCACCCTCTATCTAGTATTTGGTGCCTGAGCCGGAATAGTGGGCACAGCCCT
AAGCCTGCTCATTCGAGCAGAACTAAGCCAACCAGGCGCCCTCCTAGGAGACGACCA
GATTTATAATGTAATTGTTACAGCACATGCATTCGTAATAATTTTCTTTATAGTAATGCCA
ATTATAATCGGAGGATTCGGAAACTGACTCGTACCACTAATAATTGGTGCCCCAGACAT
AGCATTCCCTCGAATGAATAACATGAGCTTCTGACTACTCCCACCGTCCTTCCTTCTCCT
TCTTGCCTCTTCCGGAGTAGAAGCTGGGGCCGGAACGGGATGAACCGTTTACCCACCC
CTGGCGGGTAACTTAGCCCATGCAGGGGCATCCGTCGACCTGACCATTTTTCTCTCCA
CTTAGCAGGAATTTCTTCCATCCTTGGAGCTATCAACTTCATTACAACTATTATTAACAT
GAAACCCCCTGCCATCTCCCAATACCAAACCCCTCTGTTCGTGTGGGCAGTCCTGATTA
CTGCTGTACTTCTTCTGCTCTCCCTACCCGTCCTGGCTGCTGGCATTACAATGCTTCTTA
CAGACCGAAACCTTAACACCACCTTCTTCGACCCCGCTGGAGGAGGTGACCCAAT
>Siniperca_sp_SZ2972
GACATTGGCACCCTCTATCTAGTATTTGGTGCCTGAGCCGGAATAGTGGGCACAGCCCT
AAGCCTGCTCATTCGAGCAGAACTAAGCCAACCAGGCGCCCTCCTAGGAGACGACCA
GATTTATAATGTAATTGTTACAGCACATGCATTCGTAATAATTTTCTTTATAGTAATGCCA

ATTATAATCGGAGGATTCGGAAACTGACTCGTACCACTAATAATTGGTGCCCCAGACAT
AGCATTCCCTCGAATGAATAACATGAGCTTCTGACTACTCCCACCGTCCTTCCTTCTCCT
TCTTGCCTCTTCCGGAGTAGAAGCTGGGGCCGGAACGGGATGAACCGTTTACCCACCC
CTGGCGGGTAACTTAGCCCATGCAGGGGCATCCGTCGACCTGACCATTTTTCTCTCCA
CTTAGCAGGAATTTCTTCCATCCTTGGAGCTATCAACTTCATTACAACTATTATTAACAT
GAAACCCCCTGCCATCTCCCAATACCAAACCCCTCTGTTCGTGTGGGCAGTCCTGATTA
CTGCTGTACTTCTTCTGCTCTCCCTACCCGTCCTGGCTGCTGGCATTACAATGCTTCTTA
CAGACCGAAACCTTAACACCACCTTCTTCGACCCCGCTGGAGGAGGTGACCCAAT
>Siniperca_sp_SZ2979
GACATTGGCACCCTCTATCTAGTATTTGGTGCCTGAGCCGGAATAGTGGGCACAGCCCT
AAGCCTGCTCATTCGAGCAGAACTAAGCCAACCAGGCGCCCTCCTAGGAGACGACCA
GATTTATAATGTAATTGTTACAGCACATGCATTCGTAATAATTTTCTTTATAGTAATGCCA
ATTATAATCGGAGGATTCGGAAACTGACTCGTACCACTAATAATTGGTGCCCCAGACAT
AGCATTCCCTCGAATGAATAACATGAGCTTCTGACTACTCCCACCGTCCTTCCTTCTCCT
TCTTGCCTCTTCCGGAGTAGAAGCTGGGGCCGGAACGGGATGAACCGTTTACCCACCC
CTGGCGGGTAACTTAGCCCATGCAGGGGCATCCGTCGACCTGACCATTTTTCTCTCCA
CTTAGCAGGAATTTCTTCCATCCTTGGAGCTATCAACTTCATTACAACTATTATTAACAT
GAAACCCCCTGCCATCTCCCAATACCAAACCCCTCTGTTCGTGTGGGCAGTCCTGATTA
CTGCTGTACTTCTTCTGCTCTCCCTACCCGTCCTGGCTGCTGGCATTACAATGCTTCTTA
CAGACCGAAACCTTAACACCACCTTCTTCGACCCCGCTGGAGGAGGTGACCCAAT
>Siniperca_sp_SZ2984
GACATTGGCACCCTCTATCTAGTATTTGGTGCCTGAGCCGGAATAGTGGGCACAGCCCT
AAGCCTGCTCATTCGAGCAGAACTAAGCCAACCAGGCGCCCTCCTAGGAGACGACCA
GATTTATAATGTAATTGTTACAGCACATGCATTCGTAATAATTTTCTTTATAGTAATGCCA
ATTATAATCGGAGGATTCGGAAACTGACTCGTACCACTAATAATTGGTGCCCCAGACAT
AGCATTCCCTCGAATGAATAACATGAGCTTCTGACTACTCCCACCGTCCTTCCTTCTCCT
TCTTGCCTCTTCCGGAGTAGAAGCTGGGGCCGGAACGGGATGAACCGTTTACCCACCC
CTGGCGGGTAACTTAGCCCATGCAGGGGCATCCGTCGACCTGACCATTTTTCTCTCCA
CTTAGCAGGAATTTCTTCCATCCTTGGAGCTATCAACTTCATTACAACTATTATTAACAT
GAAACCCCCTGCCATCTCCCAATACCAAACCCCTCTGTTCGTGTGGGCAGTCCTGATTA
CTGCTGTACTTCTTCTGCTCTCCCTACCCGTCCTGGCTGCTGGCATTACAATGCTTCTTA
CAGACCGAAACCTTAACACCACCTTCTTCGACCCCGCTGGAGGAGGTGACCCAAT
>Siniperca_sp_SZ3
GACATTGGCACCCTCTATCTAGTATTTGGTGCCTGAGCCGGAATAGTGGGCACAGCCCT
AAGCCTGCTCATTCGAGCAGAACTAAGCCAACCAGGCGCCCTCCTAGGAGACGACCA
GATTTATAATGTAATTGTTACAGCACATGCATTCGTAATAATTTTCTTTATAGTAATGCCA
ATTATAATCGGAGGATTCGGAAACTGACTCGTACCACTAATAATTGGTGCCCCAGACAT
AGCATTCCCTCGAATGAATAACATGAGCTTCTGACTACTCCCACCGTCCTTCCTTCTCCT
TCTTGCCTCTTCCGGAGTAGAAGCTGGGGCCGGAACGGGATGAACCGTTTACCCACCC
CTGGCGGGTAACTTAGCCCATGCAGGGGCATCCGTCGACCTGACCATTTTTCTCTCCA
CTTAGCAGGAATTTCTTCCATCCTTGGAGCTATCAACTTCATTACAACTATTATTAACAT
GAAACCCCCTGCCATCTCCCAATACCAAACCCCTCTGTTCGTGTGGGCAGTCCTGATTA
CTGCTGTACTTCTTCTGCTCTCCCTACCCGTCCTGGCTGCTGGCATTACAATGCTTCTTA
CAGACCGAAACCTTAACACCACCTTCTTCGACCCCGCTGGAGGAGGTGACCCAAT

>Siniperca_sp_SZ32
GACATTGGCACCCTCTATCTAGTATTTGGTGCCTGAGCCGGAATAGTGGGCACAGCCCT
AAGCCTGCTCATTCGAGCAGAACTAAGCCAACCAGGCGCCCTCCTAGGAGACGACCA
GATTTATAATGTAATTGTTACAGCACATGCATTCGTAATAATTTTCTTTATAGTAATGCCA
ATTATAATCGGAGGATTCGGAAACTGACTCGTACCACTAATAATTGGTGCCCCAGACAT
AGCATTCCCTCGAATGAATAACATGAGCTTCTGACTACTCCCACCGTCCTTCCTTCTCCT
TCTTGCCTCTTCCGGAGTAGAAGCTGGGGCCGGAACGGGATGAACCGTTTACCCACCC
CTGGCGGGTAACTTAGCCCATGCAGGGGCATCCGTCGACCTGACCATTTTTTCTCTCCA
CTTAGCAGGAATTTCTTCCATCCTTGGAGCTATCAACTTCATTACAACTATTATTAACAT
GAAACCCCCTGCCATCTCTCAATACCAAACCCCTCTGTTCGTGTGGGCAGTCCTGATTA
CTGCTGTACTTCTTCTGCTCTCCCTACCCGTCCTGGCTGCTGGCATTACAATGCTTCTTA
CAGACCGAAACCTTAACACCACCTTCTTCGACCCCGCTGGAGGAGGTGACCCAAT
>Siniperca_sp_SZ334
GACATTGGCACCCTCTATCTAGTATTTGGTGCCTGAGCCGGAATAGTGGGCACAGCCCT
AAGCCTGCTCATTCGAGCAGAACTAAGCCAACCAGGCGCCCTCCTAGGAGACGACCA
GATTTATAATGTAATTGTTACAGCACATGCATTCGTAATAATTTTCTTTATAGTAATGCCA
ATTATAATCGGAGGATTCGGAAACTGACTCGTACCACTAATAATTGGTGCCCCAGACAT
AGCATTCCCTCGAATGAATAACATGAGCTTCTGACTACTCCCACCGTCCTTCCTTCTCCT
TCTTGCCTCTTCCGGAGTAGAAGCTGGGGCCGGAACGGGATGAACCGTTTACCCACCC
CTGGCGGGTAACTTAGCCCATGCAGGGGCATCCGTCGACCTGACCATTTTTTCTCTCCA
CTTAGCAGGAATTTCTTCCATCCTTGGAGCTATCAACTTCATTACAACTATTATTAACAT
GAAACCCCCTGCCATCTCCCAATACCAAACCCCTCTGTTCGTGTGGGCAGTCCTGATTA
CTGCTGTACTTCTTCTGCTCTCCCTACCCGTCCTGGCTGCTGGCATTACAATGCTTCTTA
CAGACCGAAACCTTAACACCACCTTCTTCGACCCCGCTGGAGGAGGTGACCCAAT
>Siniperca_sp_SZ34
GACATTGGCACCCTCTATCTAGTATTTGGTGCCTGAGCCGGAATAGTGGGCACAGCCCT
AAGCCTGCTCATTCGAGCAGAACTAAGCCAACCAGGCGCCCTCCTAGGAGACGACCA
GATTTATAATGTAATTGTTACAGCACATGCATTCGTAATAATTTTCTTTATAGTAATGCCA
ATTATAATCGGAGGATTCGGAAACTGACTCGTACCACTAATAATTGGTGCCCCAGACAT
AGCATTCCCTCGAATGAATAACATGAGCTTCTGACTACTCCCACCGTCCTTCCTTCTCCT
TCTTGCCTCTTCCGGAGTAGAAGCTGGGGCCGGAACGGGATGAACCGTTTACCCACCC
CTGGCGGGTAACTTAGCCCATGCAGGGGCATCCGTCGACCTGACCATTTTTTCTCTCCA
CTTAGCAGGAATTTCTTCCATCCTTGGAGCTATCAACTTCATTACAACTATTATTAACAT
GAAACCCCCTGCCATCTCCCAATACCAAACCCCTCTGTTCGTGTGGGCAGTCCTGATTA
CTGCTGTACTTCTTCTGCTCTCCCTACCCGTCCTGGCTGCTGGCATTACAATGCTTCTTA
CAGACCGAAACCTTAACACCACCTTCTTCGACCCCGCTGGAGGAGGTGACCCAAT
>Siniperca_sp_SZ346
GACATTGGCACCCTCTATCTAGTATTTGGTGCCTGAGCCGGAATAGTGGGCACAGCCCT
AAGCCTGCTCATTCGAGCAGAACTAAGCCAACCAGGCGCCCTCCTAGGAGACGACCA
GATTTATAATGTAATTGTTACAGCACATGCATTCGTAATAATTTTCTTTATAGTAATGCCA
ATTATAATCGGAGGATTCGGAAACTGACTCGTACCACTAATAATTGGTGCCCCAGACAT
AGCATTCCCTCGAATGAATAACATGAGCTTCTGACTACTCCCACCGTCCTTCCTTCTCCT
TCTTGCCTCTTCCGGAGTAGAAGCTGGGGCCGGAACGGGATGAACCGTTTACCCACCC
CTGGCGGGTAACTTAGCCCATGCAGGGGCATCCGTCGACCTGACCATTTTTTCTCTCCA

CTTAGCAGGAATTTCTTCCATCCTTGGAGCTATCAACTTCATTACAACTATTATTAACAT
GAAACCCCCTGCCATCTCCCAATACCAAACCCCTCTGTTCGTGTGGGCAGTCCTGATTA
CTGCTGTACTTCTTCTGCTCTCCCTACCCGTCCTGGCTGCTGGCATTACAATGCTTCTTA
CAGACCGAAACCTTAACACCACCTTCTTCGACCCCGCTGGAGGAGGTGACCCAAT
>Siniperca_sp_SZ352
GACATTGGCACCCTCTATCTAGTATTTGGTGCCTGAGCCGGAATAGTGGGCACAGCCCT
AAGCCTGCTCATTCGAGCAGAACTAAGCCAACCAGGCGCCCTCCTAGGAGACGACCA
GATTTATAATGTAATTGTTACAGCACATGCATTCGTAATAATTTTCTTTATAGTAATGCCA
ATTATAATCGGAGGATTCGGAAACTGACTCGTACCACTAATAATTGGTGCCCCAGACAT
AGCATTCCCTCGAATGAATAACATGAGCTTCTGACTACTCCCACCGTCCTTCCTTCTCCT
TCTTGCCTCTTCCGGAGTAGAAGCTGGGGCCGGAACGGGATGAACCGTTTACCCACCC
CTGGCGGGTAACTTAGCCCATGCAGGGGCATCCGTCGACCTGACCATTTTTTCTCTCCA
CTTAGCAGGAATTTCTTCCATCCTTGGAGCTATCAACTTCATTACAACTATTATTAACAT
GAAACCCCCTGCCATCTCCCAATACCAAACCCCTCTGTTCGTGTGGGCAGTCCTGATTA
CTGCTGTACTTCTTCTGCTCTCCCTACCCGTCCTGGCTGCTGGCATTACAATGCTTCTTA
CAGACCGAAACCTTAACACCACCTTCTTCGACCCCGCTGGAGGAGGTGACCCAAT
>Siniperca_sp_SZ36
GACATTGGCACCCTCTATCTAGTATTTGGTGCCTGAGCCGGAATAGTGGGCACAGCCCT
AAGCCTGCTCATTCGAGCAGAACTAAGCCAACCAGGCGCCCTCCTAGGAGACGACCA
GATTTATAATGTAATTGTTACAGCACATGCATTCGTAATAATTTTCTTTATAGTAATGCCA
ATTATAATCGGAGGATTCGGAAACTGACTCGTACCACTAATAATTGGTGCCCCAGACAT
AGCATTCCCTCGAATGAATAACATGAGCTTCTGACTACTCCCACCGTCCTTCCTTCTCCT
TCTTGCCTCTTCCGGAGTAGAAGCTGGGGCCGGAACGGGATGAACCGTTTACCCACCC
CTGGCGGGTAACTTAGCCCATGCAGGGGCATCCGTCGACCTGACCATTTTTTCTCTCCA
CTTAGCAGGAATTTCTTCCATCCTTGGAGCTATCAACTTCATTACAACTATTATTAACAT
GAAACCCCCTGCCATCTCCCAATACCAAACCCCTCTGTTCGTGTGGGCAGTCCTGATTA
CTGCTGTACTTCTTCTGCTCTCCCTACCCGTCCTGGCTGCTGGCATTACAATGCTTCTTA
CAGACCGAAACCTTAACACCACCTTCTTCGACCCCGCTGGAGGAGGTGACCCAAT
>Siniperca_sp_SZ37
GACATTGGCACCCTCTATCTAGTATTTGGTGCCTGAGCCGGAATAGTGGGCACAGCCCT
AAGCCTGCTCATTCGAGCAGAACTAAGCCAACCAGGCGCCCTCCTAGGAGACGACCA
GATTTATAATGTAATTGTTACAGCACATGCATTCGTAATAATTTTCTTTATAGTAATGCCA
ATTATAATCGGAGGATTCGGAAACTGACTCGTACCACTAATAATTGGTGCCCCAGACAT
AGCATTCCCTCGAATGAATAACATGAGCTTCTGACTACTCCCACCGTCCTTCCTTCTCCT
TCTTGCCTCTTCCGGAGTAGAAGCTGGGGCCGGAACGGGATGAACCGTTTACCCACCC
CTGGCGGGTAACTTAGCCCATGCAGGGGCATCCGTCGACCTGACCATTTTTTCTCTCCA
CTTAGCAGGAATTTCTTCCATCCTTGGAGCTATCAACTTCATTACAACTATTATTAACAT
GAAACCCCCTGCCATCTCCCAATACCAAACCCCTCTGTTCGTGTGGGCAGTCCTGATTA
CTGCTGTACTTCTTCTGCTCTCCCTACCCGTCCTGGCTGCTGGCATTACAATGCTTCTTA
CAGACCGAAACCTTAACACCACCTTCTTCGACCCCGCTGGAGGAGGTGACCCAAT
>Siniperca_sp_SZ38
GACATTGGCACCCTCTATCTAGTATTTGGTGCCTGAGCCGGAATAGTGGGCACAGCCCT
AAGCCTGCTCATTCGAGCAGAACTAAGCCAACCAGGCGCCCTCCTAGGAGACGACCA
GATTTATAATGTAATTGTTACAGCACATGCATTCGTAATAATTTTCTTTATAGTAATGCCA

ATTATAATCGGAGGATTCGGAAACTGACTCGTACCACTAATAATTGGTGCCCCAGACAT
AGCATTCCCTCGAATGAATAACATGAGCTTCTGACTACTCCCACCGTCCTTCCTTCTCCT
TCTTGCCTCTTCCGGAGTAGAAGCTGGGGCCGGAACGGGATGAACCGTTTACCCACCC
CTGGCGGGTAACTTAGCCCATGCAGGGGCATCCGTCGACCTGACCATTTTTCTCTCCA
CTTAGCAGGAATTTCTTCCATCCTTGGAGCTATCAACTTCATTACAACTATTATTAACATA
AAACCCCCTGCCATCTCCCAATACCAAACCCCTCTGTTCGTGTGGGCAGTCCTGATTAC
TGCTGTACTTCTTCTGCTCTCCCTACCCGTCCTGGCTGCTGGCATTACAATGCTTCTTAC
AGACCGAAACCTTAACACCACCTTCTTCGACCCCGCTGGAGGAGGTGACCCAAT
>Siniperca_sp_SZ384
GACATTGGCACCCTCTATCTAGTATTTGGTGCCTGAGCCGGAATAGTGGGCACAGCCCT
AAGCCTGCTCATTCGAGCAGAACTAAGCCAACCAGGCGCCCTCCTAGGAGACGACCA
GATTTATAATGTAATTGTTACAGCACATGCATTCGTAATAATTTTCTTTATAGTAATGCCA
ATTATAATCGGAGGATTCGGAAACTGACTCGTACCACTAATAATTGGTGCCCCAGACAT
AGCATTCCCTCGAATGAATAACATGAGCTTCTGACTACTCCCACCGTCCTTCCTTCTCCT
TCTTGCCTCTTCCGGAGTAGAAGCTGGGGCCGGAACGGGATGAACCGTTTACCCACCC
CTGGCGGGTAACTTAGCCCATGCAGGGGCATCCGTCGACCTGACCATTTTTCTCTCCA
CTTAGCAGGAATTTCTTCCATCCTTGGAGCTATCAACTTCATTACAACTATTATTAACAT
GAAACCCCCTGCCATCTCCCAATACCAAACCCCTCTGTTCGTGTGGGCAGTCCTGATTA
CTGCTGTACTTCTTCTGCTCTCCCTACCCGTCCTGGCTGCTGGCATTACAATGCTTCTTA
CAGACCGAAACCTTAACACCACCTTCTTCGACCCCGCTGGAGGAGGTGACCCAAT
>Siniperca_sp_SZ386
GACATTGGCACCCTCTATCTAGTATTTGGTGCCTGAGCCGGAATAGTGGGCACAGCCCT
AAGCCTGCTCATTCGAGCAGAACTAAGCCAACCAGGCGCCCTCCTAGGAGACGACCA
GATTTATAATGTAATTGTTACAGCACATGCATTCGTAATAATTTTCTTTATAGTAATGCCA
ATTATAATCGGAGGATTCGGAAACTGACTCGTACCACTAATAATTGGTGCCCCAGACAT
AGCATTCCCTCGAATGAATAACATGAGCTTCTGACTACTCCCACCGTCCTTCCTTCTCCT
TCTTGCCTCTTCCGGAGTAGAAGCTGGGGCCGGAACGGGATGAACCGTTTACCCACCC
CTGGCGGGTAACTTAGCCCATGCAGGGGCATCCGTCGACCTGACCATTTTTCTCTCCA
CTTAGCAGGAATTTCTTCCATCCTTGGAGCTATCAACTTCATTACAACTATTATTAACAT
GAAACCCCCTGCCATCTCCCAATACCAAACCCCTCTGTTCGTGTGGGCAGTCCTGATTA
CTGCTGTACTTCTTCTGCTCTCCCTACCCGTCCTGGCTGCTGGCATTACAATGCTTCTTA
CAGACCGAAACCTTAACACCACCTTCTTCGACCCCGCTGGAGGAGGTGACCCAAT
>Siniperca_sp_SZ39
GACATTGGCACCCTCTATCTAGTATTTGGTGCCTGAGCCGGAATAGTGGGCACAGCCCT
AAGCCTGCTCATTCGAGCAGAACTAAGCCAACCAGGCGCCCTCCTAGGAGACGACCA
GATTTATAATGTAATTGTTACAGCACATGCATTCGTAATAATTTTCTTTATAGTAATGCCA
ATTATAATCGGAGGATTCGGAAACTGACTCGTACCACTAATAATTGGTGCCCCAGACAT
AGCATTCCCTCGAATGAATAACATGAGCTTCTGACTACTCCCACCGTCCTTCCTTCTCCT
TCTTGCCTCTTCCGGAGTAGAAGCTGGGGCCGGAACGGGATGAACCGTTTACCCACCC
CTGGCGGGTAACTTAGCCCATGCAGGGGCATCCGTCGACCTGACCATTTTTCTCTCCA
CTTAGCAGGAATTTCTTCCATCCTTGGAGCTATCAACTTCATTACAACTATTATTAACAT
GAAACCCCCTGCCATCTCTCAATACCAAACCCCTCTGTTCGTGTGGGCAGTCCTGATTA
CTGCTGTACTTCTTCTGCTCTCCCTACCCGTCCTGGCTGCTGGCATTACAATGCTTCTTA
CAGACCGAAACCTTAACACCACCTTCTTCGACCCCGCTGGAGGAGGTGACCCAAT

>Siniperca_sp_SZ40
GACATTGGCACCCTCTATCTAGTATTTGGTGCCTGAGCCGGAATAGTGGGCACAGCCCT
AAGCCTGCTCATTCGAGCAGAACTAAGCCAACCAGGCGCCCTCCTAGGAGACGACCA
GATTTATAATGTAATTGTTACAGCACATGCATTCGTAATAATTTTCTTTATAGTAATGCCA
ATTATAATCGGAGGATTCGGAAACTGACTCGTACCACTAATAATTGGTGCCCCAGACAT
AGCATTCCCTCGAATGAATAACATAAGCTTCTGACTACTCCCACCGTCCTTCCTTCTCCT
TCTTGCCTCTTCCGGAGTAGAAGCTGGGGCCGGAACGGGATGAACCGTTTACCCACCC
CTGGCGGGTAACTTAGCCCATGCAGGGGCATCCGTCGACCTGACCATTTTTTCTCTCCA
CTTAGCAGGAATTTCTTCCATCCTTGGAGCTATCAACTTCATTACAACTATTATTAACAT
GAAACCCCCTGCCATCTCCCAATACCAAACCCCTCTGTTCGTGTGGGCAGTCCTGATTA
CTGCTGTACTTCTTCTGCTCTCCCTACCCGTCCTGGCTGCTGGCATTACAATGCTTCTTA
CAGACCGAAACCTTAACACCACCTTCTTCGACCCCGCTGGAGGAGGTGACCCAAT
>Siniperca_sp_SZ41
GACATTGGCACCCTCTATCTAGTATTTGGTGCCTGAGCCGGAATAGTGGGCACAGCCCT
AAGCCTGCTCATTCGAGCAGAACTAAGCCAACCAGGCGCCCTCCTAGGAGACGACCA
GATTTATAATGTAATTGTTACAGCACATGCATTCGTAATAATTTTCTTTATAGTAATGCCA
ATTATAATCGGAGGATTCGGAAACTGACTCGTACCACTAATAATTGGTGCCCCAGACAT
AGCATTCCCTCGAATGAATAACATGAGCTTCTGACTACTCCCACCGTCCTTCCTTCTCCT
TCTTGCCTCTTCCGGAGTAGAAGCTGGGGCCGGAACGGGATGAACCGTTTACCCACCC
CTGGCGGGTAACTTAGCCCATGCAGGGGCATCCGTCGACCTGACCATTTTTTCTCTCCA
CTTAGCAGGAATTTCTTCCATCCTTGGAGCTATCAACTTCATTACAACTATTATTAACAT
GAAACCCCCTGCCATCTCCCAATACCAAACCCCTCTGTTCGTGTGGGCAGTCCTGATTA
CTGCTGTACTTCTTCTGCTCTCCCTACCCGTCCTGGCTGCTGGCATTACAATGCTTCTTA
CAGACCGAAACCTTAACACCACCTTCTTCGACCCCGCTGGAGGAGGTGACCCAAT
>Siniperca_sp_SZ43
GACATTGGCACCCTCTATCTAGTATTTGGTGCCTGAGCCGGAATAGTGGGCACAGCCCT
AAGCCTGCTCATTCGAGCAGAACTAAGCCAACCAGGCGCCCTCCTAGGAGACGACCA
GATTTATAATGTAATTGTTACAGCACATGCATTCGTAATAATTTTCTTTATAGTAATGCCA
ATTATAATCGGAGGATTCGGAAACTGACTCGTACCACTAATAATTGGTGCCCCAGACAT
AGCATTCCCTCGAATGAATAACATGAGCTTCTGACTACTCCCACCGTCCTTCCTTCTCCT
TCTTGCCTCTTCCGGAGTAGAAGCTGGGGCCGGAACGGGATGAACCGTTTACCCACCC
CTGGCGGGTAACTTAGCCCATGCAGGGGCATCCGTCGACCTGACCATTTTTTCTCTCCA
CTTAGCAGGAATTTCTTCCATCCTTGGAGCTATCAACTTCATTACAACTATTATTAACAT
GAAACCCCCTGCCATCTCCCAATACCAAACCCCTCTGTTCGTGTGGGCAGTCCTGATTA
CTGCTGTACTTCTTCTGCTCTCCCTACCCGTCCTGGCTGCTGGCATTACAATGCTTCTTA
CAGACCGAAACCTTAACACCACCTTCTTCGACCCCGCTGGAGGAGGTGACCCAAT
>Siniperca_sp_SZ470
GACATTGGCACCCTCTATCTAGTATTTGGTGCCTGAGCCGGAATAGTGGGCACAGCCCT
AAGCCTGCTCATTCGAGCAGAACTAAGCCAACCAGGCGCCCTCCTAGGAGACGACCA
GATTTATAATGTAATTGTTACAGCACATGCATTCGTAATAATTTTCTTTATAGTAATGCCA
ATTATAATCGGAGGATTCGGAAACTGACTCGTACCACTAATAATTGGTGCCCCAGACAT
AGCATTCCCTCGAATGAATAACATGAGCTTCTGACTACTCCCACCGTCCTTCCTTCTCCT
TCTTGCCTCTTCCGGAGTAGAAGCTGGGGCCGGAACGGGATGAACCGTTTACCCACCC
CTGGCGGGTAACTTAGCCCATGCAGGGGCATCCGTCGACCTGACCATTTTTTCTCTCCA

CTTAGCAGGAATTTCTTCCATCCTTGGAGCTATCAACTTCATTACAACTATTATTAACAT
GAAACCCCCTGCCATCTCTCAATACCAAACCCCTCTGTTCGTGTGGGCAGTCCTGATTA
CTGCTGTACTTCTTCTGCTCTCCCTACCCGTCCTGGCTGCTGGCATTACAATGCTTCTTA
CAGACCGAAACCTTAACACCACCTTCTTCGACCCCGCTGGAGGAGGTGACCCAAT
>Siniperca_sp_SZ522
GACATTGGCACCCTCTATCTAGTATTTGGTGCCTGAGCCGGAATAGTGGGCACAGCCCT
AAGCCTGCTCATTCGAGCAGAACTAAGCCAACCAGGCGCCCTCCTAGGAGACGACCA
GATTTATAATGTAATTGTTACAGCACATGCATTCGTAATAATTTTCTTTATAGTAATGCCA
ATTATAATCGGAGGATTCGGAAACTGACTCGTACCACTAATAATTGGTGCCCCAGACAT
AGCATTCCCTCGAATGAATAACATGAGCTTCTGACTACTCCCACCGTCCTTCCTTCTCCT
TCTTGCCTCTTCCGGAGTAGAAGCTGGGGCCGGAACGGGATGAACCGTTTACCCACCC
CTGGCGGGTAACTTAGCCCATGCAGGGGCATCCGTCGACCTGACCATTTTTTCTCTCCA
CTTAGCAGGAATTTCTTCCATCCTTGGAGCTATCAACTTCATTACAACTATTATTAACAT
GAAACCCCCTGCCATCTCCCAATACCAAACCCCTCTGTTCGTGTGGGCAGTCCTGATTA
CTGCTGTACTTCTTCTGCTCTCCCTACCCGTCCTGGCTGCTGGCATTACAATGCTTCTTA
CAGACCGAAACCTTAACACCACCTTCTTCGACCCCGCTGGAGGAGGTGACCCAAT
>Siniperca_sp_SZ535
GACATTGGCACCCTCTATCTAGTATTTGGTGCCTGAGCCGGAATAGTGGGCACAGCCCT
AAGCCTGCTCATTCGAGCAGAACTAAGCCAACCAGGCGCCCTCCTAGGAGACGACCA
GATTTATAATGTAATTGTTACAGCACATGCATTCGTAATAATTTTCTTTATAGTAATGCCA
ATTATAATCGGAGGATTCGGAAACTGACTCGTACCACTAATAATTGGTGCCCCAGACAT
AGCATTCCCTCGAATGAATAACATGAGCTTCTGACTACTCCCACCGTCCTTCCTTCTCCT
TCTTGCCTCTTCCGGAGTAGAAGCTGGGGCCGGAACGGGATGAACCGTTTACCCACCC
CTGGCGGGTAACTTAGCCCATGCAGGGGCATCCGTCGACCTGACCATTTTTTCTCTCCA
CTTAGCAGGAATTTCTTCCATCCTTGGAGCTATCAACTTCATTACAACTATTATTAACAT
GAAACCCCCTGCCATCTCCCAATACCAAACCCCTCTGTTCGTGTGGGCAGTCCTGATTA
CTGCTGTACTTCTTCTGCTCTCCCTACCCGTCCTGGCTGCTGGCATTACAATGCTTCTTA
CAGACCGAAACCTTAACACCACCTTCTTCGACCCCGCTGGAGGAGGTGACCCAAT
>Siniperca_sp_SZ543
GACATTGGCACCCTCTATCTAGTATTTGGTGCCTGAGCCGGAATAGTGGGCACAGCCCT
AAGCCTGCTCATTCGAGCAGAACTAAGCCAACCAGGCGCCCTCCTAGGAGACGACCA
GATTTATAATGTAATTGTTACAGCACATGCATTCGTAATAATTTTCTTTATAGTAATGCCA
ATTATAATCGGAGGATTCGGAAACTGACTCGTACCACTAATAATTGGTGCCCCAGACAT
AGCATTCCCTCGAATGAATAACATGAGCTTCTGACTACTCCCACCGTCCTTCCTTCTCCT
TCTTGCCTCTTCCGGAGTAGAAGCTGGGGCCGGAACGGGATGAACCGTTTACCCACCC
CTGGCGGGTAACTTAGCCCATGCAGGGGCATCCGTCGACCTGACCATTTTTTCTCTCCA
CTTAGCAGGAATTTCTTCCATCCTTGGAGCTATCAACTTCATTACAACTATTATTAACAT
GAAACCCCCTGCCATCTCCCAATACCAAACCCCTCTGTTCGTGTGGGCAGTCCTGATTA
CTGCTGTACTTCTTCTGCTCTCCCTACCCGTCCTGGCTGCTGGCATTACAATGCTTCTTA
CAGACCGAAACCTTAACACCACCTTCTTCGACCCCGCTGGAGGAGGTGACCCAAT
>Siniperca_sp_SZ544
GACATTGGCACCCTCTATCTAGTATTTGGTGCCTGAGCCGGAATAGTGGGCACAGCCCT
AAGCCTGCTCATTCGAGCAGAACTAAGCCAACCAGGCGCCCTCCTAGGAGACGACCA
GATTTATAATGTAATTGTTACAGCACATGCATTCGTAATAATTTTCTTTATAGTAATGCCA

ATTATAATCGGAGGATTCGGAAACTGACTCGTACCACTAATAATTGGTGCCCCAGACAT
AGCATTCCCTCGAATGAATAACATGAGCTTCTGACTACTCCCACCGTCCTTCCTTCTCCT
TCTTGCCTCTTCCGGAGTAGAAGCTGGGGCCGGAACGGGATGAACCGTTTACCCACCC
CTGGCGGGTAACTTAGCCCATGCAGGGGCATCCGTCGACCTGACCATTTTTCTCTCCA
CTTAGCAGGAATTTCTTCCATCCTTGGAGCTATCAACTTCATTACAACTATTATTAACAT
GAAACCCCCTGCCATCTCCCAATACCAAACCCCTCTGTTCGTGTGGGCAGTCCTGATTA
CTGCTGTACTTCTTCTGCTCTCCCTACCCGTCCTGGCTGCTGGCATTACAATGCTTCTTA
CAGACCGAAACCTTAACACCACCTTCTTCGACCCCGCTGGAGGAGGTGACCCAAT
>Siniperca_sp_SZ545
GACATTGGCACCCTCTATCTAGTATTTGGTGCCTGAGCCGGAATAGTGGGCACAGCCCT
AAGCCTGCTCATTCGAGCAGAACTAAGCCAACCAGGCGCCCTCCTAGGAGACGACCA
GATTTATAATGTAATTGTTACAGCACATGCATTCGTAATAATTTTCTTTATAGTAATGCCA
ATTATAATCGGAGGATTCGGAAACTGACTCGTACCACTAATAATTGGTGCCCCAGACAT
AGCATTCCCTCGAATGAATAACATGAGCTTCTGACTACTCCCACCGTCCTTCCTTCTCCT
TCTTGCCTCTTCCGGAGTAGAAGCTGGGGCCGGAACGGGATGAACCGTTTACCCACCC
CTGGCGGGTAACTTAGCCCATGCAGGGGCATCCGTCGACCTGACCATTTTTCTCTCCA
CTTAGCAGGAATTTCTTCCATCCTTGGAGCTATCAACTTCATTACAACTATTATTAACAT
GAAACCCCCTGCCATCTCCCAATACCAAACCCCTCTGTTCGTGTGGGCAGTCCTGATTA
CTGCTGTACTTCTTCTGCTCTCCCTACCCGTCCTGGCTGCTGGCATTACAATGCTTCTTA
CAGACCGAAACCTTAACACCACCTTCTTCGACCCCGCTGGAGGAGGTGACCCAAT
>Siniperca_sp_SZ558
GACATTGGCACCCTCTATCTAGTATTTGGTGCCTGAGCCGGAATAGTGGGCACAGCCCT
AAGCCTGCTCATTCGAGCAGAACTAAGCCAACCAGGCGCCCTCCTAGGAGACGACCA
GATTTATAATGTAATTGTTACAGCACATGCATTCGTAATAATTTTCTTTATAGTAATGCCA
ATTATAATCGGAGGATTCGGAAACTGACTCGTACCACTAATAATTGGTGCCCCAGACAT
AGCATTCCCTCGAATGAATAACATGAGCTTCTGACTACTCCCACCGTCCTTCCTTCTCCT
TCTTGCCTCTTCCGGAGTAGAAGCTGGGGCCGGAACGGGATGAACCGTTTACCCACCC
CTGGCGGGTAACTTAGCCCATGCAGGGGCATCCGTCGACCTGACCATTTTTCTCTCCA
CTTAGCAGGAATTTCTTCCATCCTTGGAGCTATCAACTTCATTACAACTATTATTAACAT
GAAACCCCCTGCCATCTCCCAATACCAAACCCCTCTGTTCGTGTGGGCAGTCCTGATTA
CTGCTGTACTTCTTCTGCTCTCCCTACCCGTCCTGGCTGCTGGCATTACAATGCTTCTTA
CAGACCGAAACCTTAACACCACCTTCTTCGACCCCGCTGGAGGAGGTGACCCAAT
>Siniperca_sp_SZ564
GACATTGGCACCCTCTATCTAGTATTTGGTGCCTGAGCCGGAATAGTGGGCACAGCCCT
AAGCCTGCTCATTCGAGCAGAACTAAGCCAACCAGGCGCCCTCCTAGGAGACGACCA
GATTTATAATGTAATTGTTACAGCACATGCATTCGTAATAATTTTCTTTATAGTAATGCCA
ATTATAATCGGAGGATTCGGAAACTGACTCGTACCACTAATAATTGGTGCCCCAGACAT
AGCATTCCCTCGAATGAATAACATGAGCTTCTGACTACTCCCACCGTCCTTCCTTCTCCT
TCTTGCCTCTTCCGGAGTAGAAGCTGGGGCCGGAACGGGATGAACCGTTTACCCACCC
CTGGCGGGTAACTTAGCCCATGCAGGGGCATCCGTCGACCTGACCATTTTTCTCTCCA
CTTAGCAGGAATTTCTTCCATCCTTGGAGCTATCAACTTCATTACAACTATTATTAACAT
GAAACCCCCTGCCATCTCTCAATACCAAACCCCTCTGTTCGTGTGGGCAGTCCTGATTA
CTGCTGTACTTCTTCTGCTCTCCCTACCCGTCCTGGCTGCTGGCATTACAATGCTTCTTA
CAGACCGAAACCTTAACACCACCTTCTTCGACCCCGCTGGAGGAGGTGACCCAAT

>Siniperca_sp_SZ565
GACATTGGCACCCTCTATCTAGTATTTGGTGCCTGAGCCGGAATAGTGGGCACAGCCCT
AAGCCTGCTCATTCGAGCAGAACTAAGCCAACCAGGCGCCCTCCTAGGAGACGACCA
GATTTATAATGTAATTGTTACAGCACATGCATTCGTAATAATTTTCTTTATAGTAATGCCA
ATTATAATCGGAGGATTCGGAAACTGACTCGTACCACTAATAATTGGTGCCCCAGACAT
AGCATTCCCTCGAATGAATAACATGAGCTTCTGACTACTCCCACCGTCCTTCCTTCTCCT
TCTTGCCTCTTCCGGAGTAGAAGCTGGGGCCGGAACGGGATGAACCGTTTACCCACCC
CTGGCGGGTAACTTAGCCCATGCAGGGGCATCCGTCGACCTGACCATTTTTTCTCTCCA
CTTAGCAGGAATTTCTTCCATCCTTGGAGCTATCAACTTCATTACAACTATTATTAACAT
GAAACCCCCTGCCATCTCCCAATACCAAACCCCTCTGTTCGTGTGGGCAGTCCTGATTA
CTGCTGTACTTCTTCTGCTCTCCCTACCCGTCCTGGCTGCTGGCATTACAATGCTTCTTA
CAGACCGAAACCTTAACACCACCTTCTTCGACCCCGCTGGAGGAGGTGACCCAAT
>Siniperca_sp_SZ568
GACATTGGCACCCTCTATCTAGTATTTGGTGCCTGAGCCGGAATAGTGGGCACAGCCCT
AAGCCTGCTCATTCGAGCAGAACTAAGCCAACCAGGCGCCCTCCTAGGAGACGACCA
GATTTATAATGTAATTGTTACAGCACATGCATTCGTAATAATTTTCTTTATAGTAATGCCA
ATTATAATCGGAGGATTCGGAAACTGACTCGTACCACTAATAATTGGTGCCCCAGACAT
AGCATTCCCTCGAATGAATAACATGAGCTTCTGACTACTCCCACCGTCCTTCCTTCTCCT
TCTTGCCTCTTCCGGAGTAGAAGCTGGGGCCGGAACGGGATGAACCGTTTACCCACCC
CTGGCGGGTAACTTAGCCCATGCAGGGGCATCCGTCGACCTGACCATTTTTTCTCTCCA
CTTAGCAGGAATTTCTTCCATCCTTGGAGCTATCAACTTCATTACAACTATTATTAACAT
GAAACCCCCTGCCATCTCCCAATACCAAACCCCTCTGTTCGTGTGGGCAGTCCTGATTA
CTGCTGTACTTCTTCTGCTCTCCCTACCCGTCCTGGCTGCTGGCATTACAATGCTTCTTA
CAGACCGAAACCTTAACACCACCTTCTTCGACCCCGCTGGAGGAGGTGACCCAAT
>Siniperca_sp_SZ570
GACATTGGCACCCTCTATCTAGTATTTGGTGCCTGAGCCGGAATAGTGGGCACAGCCCT
AAGCCTGCTCATTCGAGCAGAACTAAGCCAACCAGGCGCCCTCCTAGGAGACGACCA
GATTTATAATGTAATTGTTACAGCACATGCATTCGTAATAATTTTCTTTATAGTAATGCCA
ATTATAATCGGAGGATTCGGAAACTGACTCGTACCACTAATAATTGGTGCCCCAGACAT
AGCATTCCCTCGAATGAATAACATGAGCTTCTGACTACTCCCACCGTCCTTCCTTCTCCT
TCTTGCCTCTTCCGGAGTAGAAGCTGGGGCCGGAACGGGATGAACCGTTTACCCACCC
CTGGCGGGTAACTTAGCCCATGCAGGGGCATCCGTCGACCTGACCATTTTTTCTCTCCA
CTTAGCAGGAATTTCTTCCATCCTTGGAGCTATCAACTTCATTACAACTATTATTAACAT
GAAACCCCCTGCCATCTCCCAATACCAAACCCCTCTGTTCGTGTGGGCAGTCCTGATTA
CTGCTGTACTTCTTCTGCTCTCCCTACCCGTCCTGGCTGCTGGCATTACAATGCTTCTTA
CAGACCGAAACCTTAACACCACCTTCTTCGACCCCGCTGGAGGAGGTGACCCAAT
>Siniperca_sp_SZ603
GACATTGGCACCCTCTATCTAGTATTTGGTGCCTGAGCCGGAATAGTGGGCACAGCCCT
AAGCCTGCTCATTCGAGCAGAACTAAGCCAACCAGGCGCCCTCCTAGGAGACGACCA
GATTTATAATGTAATTGTTACAGCACATGCATTCGTAATAATTTTCTTTATAGTAATGCCA
ATTATAATCGGAGGATTCGGAAACTGACTCGTACCACTAATAATTGGTGCCCCAGACAT
AGCATTCCCTCGAATGAATAACATGAGCTTCTGACTACTCCCACCGTCCTTCCTTCTCCT
TCTTGCCTCTTCCGGAGTAGAAGCTGGGGCCGGAACGGGATGAACCGTTTACCCACCC
CTGGCGGGTAACTTAGCCCATGCAGGGGCATCCGTCGACCTGACCATTTTTTCTCTCCA

CTTAGCAGGAATTTCTTCCATCCTTGGAGCTATCAACTTCATTACAACTATTATTAACAT
GAAACCCCCTGCCATCTCCCAATACCAAACCCCTCTGTTCGTGTGGGCAGTCCTGATTA
CTGCTGTACTTCTTCTGCTCTCCCTACCCGTCCTGGCTGCTGGCATTACAATGCTTCTTA
CAGACCGAAACCTTAACACCACCTTCTTCGACCCCGCTGGAGGAGGTGACCCAAT
>Siniperca_sp_SZ608
GACATTGGCACCCTCTATCTAGTATTTGGTGCCTGAGCCGGAATAGTGGGCACAGCCCT
AAGCCTGCTCATTCGAGCAGAACTAAGCCAACCAGGCGCCCTCCTAGGAGACGACCA
GATTTATAATGTAATTGTTACAGCACATGCATTCGTAATAATTTTCTTTATAGTAATGCCA
ATTATAATCGGAGGATTCGGAAACTGACTCGTACCACTAATAATTGGTGCCCCAGACAT
AGCATTCCCTCGAATGAATAACATGAGCTTCTGACTACTCCCACCGTCCTTCCTTCTCCT
TCTTGCCTCTTCCGGAGTAGAAGCTGGAGCCGGAACGGGATGAACCGTTTACCCACCC
CTGGCGGGTAACTTAGCCCATGCAGGGGCATCCGTCGACCTGACCATTTTTTCTCTCCA
CTTAGCAGGAATTTCTTCCATCCTTGGAGCTATCAACTTCATTACAACTATTATTAACAT
GAAACCCCCTGCCATCTCCCAATACCAAACCCCTCTGTTCGTGTGGGCAGTCCTGATTA
CTGCTGTACTTCTTCTGCTCTCCCTACCCGTCCTGGCTGCTGGCATTACAATGCTTCTTA
CAGACCGAAACCTTAACACCACCTTCTTCGACCCCGCTGGAGGAGGTGACCCAAT
>Siniperca_sp_SZ61
GACATTGGCACCCTCTATCTAGTATTTGGTGCCTGAGCCGGAATAGTGGGCACAGCCCT
AAGCCTGCTCATTCGAGCAGAACTAAGCCAACCAGGCGCCCTCCTAGGAGACGACCA
GATTTATAATGTAATTGTTACAGCACATGCATTCGTAATAATTTTCTTTATAGTAATGCCA
ATTATAATCGGAGGATTCGGAAACTGACTCGTACCACTAATAATTGGTGCCCCAGACAT
AGCATTCCCTCGAATGAATAACATGAGCTTCTGACTACTCCCACCGTCCTTCCTTCTCCT
TCTTGCCTCTTCCGGAGTAGAAGCTGGGGCCGGAACGGGATGAACCGTTTACCCACCC
CTGGCGGGTAACTTAGCCCATGCAGGGGCATCCGTCGACCTGACCATTTTTTCTCTCCA
CTTAGCAGGAATTTCTTCCATCCTTGGAGCTATCAACTTCATTACAACTATTATTAACAT
GAAACCCCCTGCCATCTCCCAATACCAAACCCCTCTGTTCGTGTGGGCAGTCCTGATTA
CTGCTGTACTTCTTCTGCTCTCCCTACCCGTCCTGGCTGCTGGCATTACAATGCTTCTTA
CAGACCGAAACCTTAACACCACCTTCTTCGACCCCGCTGGAGGAGGTGACCCAAT
>Siniperca_sp_SZ654
GACATTGGCACCCTCTATCTAGTATTTGGTGCCTGAGCCGGAATAGTGGGCACAGCCCT
AAGCCTGCTCATTCGAGCAGAACTAAGCCAACCAGGCGCCCTCCTAGGAGACGACCA
GATTTATAATGTAATTGTTACAGCACATGCATTCGTAATAATTTTCTTTATAGTAATGCCA
ATTATAATCGGAGGATTCGGAAACTGACTCGTACCACTAATAATTGGTGCCCCAGACAT
AGCATTCCCTCGAATGAATAACATGAGCTTCTGACTACTCCCACCGTCCTTCCTTCTCCT
TCTTGCCTCTTCCGGAGTAGAAGCTGGGGCCGGAACGGGATGAACCGTTTACCCACCC
CTGGCGGGTAACTTAGCCCATGCAGGGGCATCCGTCGACCTGACCATTTTTTCTCTCCA
CTTAGCAGGAATTTCTTCCATCCTTGGAGCTATCAACTTCATTACAACTATTATTAACAT
GAAACCCCCTGCCATCTCCCAATACCAAACCCCTCTGTTCGTGTGGGCAGTCCTGATTA
CTGCTGTACTTCTTCTGCTCTCCCTACCCGTCCTGGCTGCTGGCATTACAATGCTTCTTA
CAGACCGAAACCTTAACACCACCTTCTTCGACCCCGCTGGAGGAGGTGACCCAAT
>Siniperca_sp_SZ658
GACATTGGCACCCTCTATCTAGTATTTGGTGCCTGAGCCGGAATAGTGGGCACAGCCCT
AAGCCTGCTCATTCGAGCAGAACTAAGCCAACCAGGCGCCCTCCTAGGAGACGACCA
GATTTATAATGTAATTGTTACAGCACATGCATTCGTAATAATTTTCTTTATAGTAATGCCA

ATTATAATCGGAGGATTCGGAAACTGACTCGTACCACTAATAATTGGTGCCCCAGACAT
AGCATTCCCTCGAATGAATAACATGAGCTTCTGACTACTCCCACCGTCCTTCCTTCTCCT
TCTTGCCTCTTCCGGAGTAGAAGCTGGGGCCGGAACGGGATGAACCGTTTACCCACCC
CTGGCGGGTAACTTAGCCCATGCAGGGGCATCCGTCGACCTGACCATTTTTTCTCTCCA
CTTAGCAGGAATTTCTTCCATCCTTGGAGCTATCAACTTCATTACAACTATTATTAACAT
GAAACCCCCTGCCATCTCCCAATACCAAACCCCTCTGTTCGTGTGGGCAGTCCTGATTA
CTGCTGTACTTCTTCTGCTCTCCCTACCCGTCCTGGCTGCTGGCATTACAATGCTTCTTA
CAGACCGAAACCTTAACACCACCTTCTTCGACCCCGCTGGAGGAGGTGACCCAAT
>Siniperca_sp_SZ674
GACATTGGCACCCTCTATCTAGTATTTGGTGCCTGAGCCGGAATAGTGGGCACAGCCCT
AAGCCTGCTCATTCGAGCAGAACTAAGCCAACCAGGCGCCCTCCTAGGAGACGACCA
GATTTATAATGTAATTGTTACAGCACATGCATTCGTAATAATTTTCTTTATAGTAATGCCA
ATTATAATCGGAGGATTCGGAAACTGACTCGTACCACTAATAATTGGTGCCCCAGACAT
AGCATTCCCTCGAATGAATAACATGAGCTTCTGACTACTCCCACCGTCCTTCCTTCTCCT
TCTTGCCTCTTCCGGAGTAGAAGCTGGGGCCGGAACGGGATGAACCGTTTACCCACCC
CTGGCGGGTAACTTAGCCCATGCAGGGGCATCCGTCGACCTGACCATTTTTTCTCTCCA
CTTAGCAGGAATTTCTTCCATCCTTGGAGCTATCAACTTCATTACAACTATTATTAACATA
AAACCCCCTGCCATCTCCCAATACCAAACCCCTCTGTTCGTGTGGGCAGTCCTGATTAC
TGCTGTACTTCTTCTGCTCTCCCTACCCGTCCTGGCTGCTGGCATTACAATGCTTCTTAC
AGACCGAAACCTTAACACCACCTTCTTCGACCCCGCTGGAGGAGGTGACCCAAT
>Siniperca_sp_SZ681
GACATTGGCACCCTCTATCTAGTATTTGGTGCCTGAGCCGGAATAGTGGGCACAGCCCT
AAGCCTGCTCATTCGAGCAGAACTAAGCCAACCAGGCGCCCTCCTAGGAGACGACCA
GATTTATAATGTAATTGTTACAGCACATGCATTCGTAATAATTTTCTTTATAGTAATGCCA
ATTATAATCGGAGGATTCGGAAACTGACTCGTACCACTAATAATTGGTGCCCCAGACAT
AGCATTCCCTCGAATGAATAACATGAGCTTCTGACTACTCCCACCGTCCTTCCTTCTCCT
TCTTGCCTCTTCCGGAGTAGAAGCTGGGGCCGGAACGGGATGAACCGTTTACCCACCC
CTGGCGGGTAACTTAGCCCATGCAGGGGCATCCGTCGACCTGACCATTTTTTCTCTCCA
CTTAGCAGGAATTTCTTCCATCCTTGGAGCTATCAACTTCATTACAACTATTATTAACAT
GAAACCCCCTGCCATCTCCCAATACCAAACCCCTCTGTTCGTGTGGGCAGTCCTGATTA
CTGCTGTACTTCTTCTGCTCTCCCTACCCGTCCTGGCTGCTGGCATTACAATGCTTCTTA
CAGACCGAAACCTTAACACCACCTTCTTCGACCCCGCTGGAGGAGGTGACCCAAT
>Siniperca_sp_SZ686
GACATTGGCACCCTCTATCTAGTATTTGGTGCCTGAGCCGGAATAGTGGGCACAGCCCT
AAGCCTGCTCATTCGAGCAGAACTAAGCCAACCAGGCGCCCTCCTAGGAGACGACCA
GATTTATAATGTAATTGTTACAGCACATGCATTCGTAATAATTTTCTTTATAGTAATGCCA
ATTATAATCGGAGGATTCGGAAACTGACTCGTACCACTAATAATTGGTGCCCCAGACAT
AGCATTCCCTCGAATGAATAACATGAGCTTCTGACTACTCCCACCGTCCTTCCTTCTCCT
TCTTGCCTCTTCCGGAGTAGAAGCTGGGGCCGGAACGGGATGAACCGTTTACCCACCC
CTGGCGGGTAACTTAGCCCATGCAGGGGCATCCGTCGACCTGACCATTTTTTCTCTCCA
CTTAGCAGGAATTTCTTCCATCCTTGGAGCTATCAACTTCATTACAACTATTATTAACAT
GAAACCCCCTGCCATCTCCCAATACCAAACCCCTCTGTTCGTGTGGGCAGTCCTGATTA
CTGCTGTACTTCTTCTGCTCTCCCTACCCGTCCTGGCTGCTGGCATTACAATGCTTCTTA
CAGACCGAAACCTTAACACCACCTTCTTCGACCCCGCTGGAGGAGGTGACCCAAT

>Siniperca_sp_SZ687
GACATTGGCACCCTCTATCTAGTATTTGGTGCCTGAGCCGGAATAGTGGGCACAGCCCT
AAGCCTGCTCATTCGAGCAGAACTAAGCCAACCAGGCGCCCTCCTAGGAGACGACCA
GATTTATAATGTAATTGTTACAGCACATGCATTCGTAATAATTTTCTTTATAGTAATGCCA
ATTATAATCGGAGGATTCGGAAACTGACTCGTACCACTAATAATTGGTGCCCCAGACAT
AGCATTCCCTCGAATGAATAACATGAGCTTCTGACTACTCCCACCGTCCTTCCTTCTCCT
TCTTGCCTCTTCCGGAGTAGAAGCTGGGGCCGGAACGGGATGAACCGTTTACCCACCC
CTGGCGGGTAACTTAGCCCATGCAGGGGCATCCGTCGACCTGACCATTTTTCTCTCCA
CTTAGCAGGAATTTCTTCCATCCTTGGAGCTATCAACTTCATTACAACTATTATTAACAT
GAAACCCCCTGCCATCTCCCAATACCAAACCCCTCTGTTCGTGTGGGCAGTCCTGATTA
CTGCTGTACTTCTTCTGCTCTCCCTACCCGTCCTGGCTGCTGGCATTACAATGCTTCTTA
CAGACCGAAACCTTAACACCACCTTCTTCGACCCCGCTGGAGGAGGTGACCCAAT
>Siniperca_sp_SZ688
GACATTGGCACCCTCTATCTAGTATTTGGTGCCTGAGCCGGAATAGTGGGCACAGCCCT
AAGCCTGCTCATTCGAGCAGAACTAAGCCAACCAGGCGCCCTCCTAGGAGACGACCA
GATTTATAATGTAATTGTTACAGCACATGCATTCGTAATAATTTTCTTTATAGTAATGCCA
ATTATAATCGGAGGATTCGGAAACTGACTCGTACCACTAATAATTGGTGCCCCAGACAT
AGCATTCCCTCGAATGAATAACATGAGCTTCTGACTACTCCCACCGTCCTTCCTTCTCCT
TCTTGCCTCTTCCGGAGTAGAAGCTGGGGCCGGAACGGGATGAACCGTTTACCCACCC
CTGGCGGGTAACTTAGCCCATGCAGGGGCATCCGTCGACCTGACCATTTTTCTCTCCA
CTTAGCAGGAATTTCTTCCATCCTTGGAGCTATCAACTTCATTACAACTATTATTAACAT
GAAACCCCCTGCCATCTCCCAATACCAAACCCCTCTGTTCGTGTGGGCAGTCCTGATTA
CTGCTGTACTTCTTCTGCTCTCCCTACCCGTCCTGGCTGCTGGCATTACAATGCTTCTTA
CAGACCGAAACCTTAACACCACCTTCTTCGACCCCGCTGGAGGAGGTGACCCAAT
>Siniperca_sp_SZ697
GACATTGGCACCCTCTATCTAGTATTTGGTGCCTGAGCCGGAATAGTGGGCACAGCCCT
AAGCCTGCTCATTCGAGCAGAACTAAGCCAACCAGGCGCCCTCCTAGGAGACGACCA
GATTTATAATGTAATTGTTACAGCACATGCATTCGTAATAATTTTCTTTATAGTAATGCCA
ATTATAATCGGAGGATTCGGAAACTGACTCGTACCACTAATAATTGGTGCCCCAGACAT
AGCATTCCCTCGAATGAATAACATGAGCTTCTGACTACTCCCACCGTCCTTCCTTCTCCT
TCTTGCCTCTTCCGGAGTAGAAGCTGGGGCCGGAACGGGATGAACCGTTTACCCACCC
CTGGCGGGTAACTTAGCCCATGCAGGGGCATCCGTCGACCTGACCATTTTTCTCTCCA
CTTAGCAGGAATTTCTTCCATCCTTGGAGCTATCAACTTCATTACAACTATTATTAACATA
AAACCCCCTGCCATCTCCCAATACCAAACCCCTCTGTTCGTGTGGGCAGTCCTGATTAC
TGCTGTACTTCTTCTGCTCTCCCTACCCGTCCTGGCTGCTGGCATTACAATGCTTCTTAC
AGACCGAAACCTTAACACCACCTTCTTCGACCCCGCTGGAGGAGGTGACCCAAT
>Siniperca_sp_SZ703
GACATTGGCACCCTCTATCTAGTATTTGGTGCCTGAGCCGGAATAGTGGGCACAGCCCT
AAGCCTGCTCATTCGAGCAGAACTAAGCCAACCAGGCGCCCTCCTAGGAGACGACCA
GATTTATAATGTAATTGTTACAGCACATGCATTCGTAATAATTTTCTTTATAGTAATGCCA
ATTATAATCGGAGGATTCGGAAACTGACTCGTACCACTAATAATTGGTGCCCCAGACAT
AGCATTCCCTCGAATGAATAACATGAGCTTCTGACTACTCCCACCGTCCTTCCTTCTCCT
TCTTGCCTCTTCCGGAGTAGAAGCTGGGGCCGGAACGGGATGAACCGTTTACCCACCC
CTGGCGGGTAACTTAGCCCATGCAGGGGCATCCGTCGACCTGACCATTTTTCTCTCCA

CTTAGCAGGAATTTCTTCCATCCTTGGAGCTATCAACTTCATTACAACTATTATTAACAT
GAAACCCCCTGCCATCTCCCAATACCAAACCCCTCTGTTCGTGTGGGCAGTCCTGATTA
CTGCTGTACTTCTTCTGCTCTCCCTACCCGTCCTGGCTGCTGGCATTACAATGCTTCTTA
CAGACCGAAACCTTAACACCACCTTCTTCGACCCCGCTGGAGGAGGTGACCCAAT
>Siniperca_sp_SZ712
GACATTGGCACCCTCTATCTAGTATTTGGTGCCTGAGCCGGAATAGTGGGCACAGCCCT
AAGCCTGCTCATTCGAGCAGAACTAAGCCAACCAGGCGCCCTCCTAGGAGACGACCA
GATTTATAATGTAATTGTTACAGCACATGCATTCGTAATAATTTTCTTTATAGTAATGCCA
ATTATAATCGGAGGATTCGGAAACTGACTCGTACCACTAATAATTGGTGCCCCAGACAT
AGCATTCCCTCGAATGAATAACATGAGCTTCTGACTACTCCCACCGTCCTTCCTTCTCCT
TCTTGCCTCTTCCGGAGTAGAAGCTGGGGCCGGAACGGGATGAACCGTTTACCCACCC
CTGGCGGGTAACTTAGCCCATGCAGGGGCATCCGTCGACCTGACCATTTTTTCTCTCCA
CTTAGCAGGAATTTCTTCCATCCTTGGAGCTATCAACTTCATTACAACTATTATTAACAT
GAAACCCCCTGCCATCTCCCAATACCAAACCCCTCTGTTCGTGTGGGCAGTCCTGATTA
CTGCTGTACTTCTTCTGCTCTCCCTACCCGTCCTGGCTGCTGGCATTACAATGCTTCTTA
CAGACCGAAACCTTAACACCACCTTCTTCGACCCCGCTGGAGGAGGTGACCCAAT
>Siniperca_sp_SZ9
GACATTGGCACCCTCTATCTAGTATTTGGTGCCTGAGCCGGAATAGTGGGCACAGCCCT
AAGCCTGCTCATTCGAGCAGAACTAAGCCAACCAGGCGCCCTCCTAGGAGACGACCA
GATTTATAATGTAATTGTTACAGCACATGCATTCGTAATAATTTTCTTTATAGTAATGCCA
ATTATAATCGGAGGATTCGGAAACTGACTCGTACCACTAATAATTGGTGCCCCAGACAT
AGCATTCCCTCGAATGAATAACATGAGCTTCTGACTACTCCCACCGTCCTTCCTTCTCCT
TCTTGCCTCTTCCGGAGTAGAAGCTGGGGCCGGAACGGGATGAACCGTTTACCCACCC
CTGGCGGGTAACTTAGCCCATGCAGGGGCATCCGTCGACCTGACCATTTTTTCTCTCCA
CTTAGCAGGAATTTCTTCCATCCTTGGAGCTATCAACTTCATTACAACTATTATTAACAT
GAAACCCCCTGCCATCTCTCAATACCAAACCCCTCTGTTCGTGTGGGCAGTCCTGATTA
CTGCTGTACTTCTTCTGCTCTCCCTACCCGTCCTGGCTGCTGGCATTACAATGCTTCTTA
CAGACCGAAACCTTAACACCACCTTCTTCGACCCCGCTGGAGGAGGTGACCCAAT
>Rhinogobius_sp 5_SZ189
GACATTGGCACCCTTTATCTTGTATTTGGTGCCTGAGCTGGAATAGTGGGCACGGCCTT
GAGCCTCCTTATTCGAGCCGAGCTAAGCCAGCCCGGAGCCCTTCTGGGCGATGACCAA
ATTTATAATGTTATCGTTACAGCTCATGCTTTCGTAATAATTTTCTTTATAGTAATACCAAT
CATGATTGGGGGTTTCGGAAACTGGCTTATTCCCCTAATAATTGGTGCGCCAGATATAGC
CTTCCCTCGGATAAATAACATAAGCTTTTGACTTCTGCCCCCATCCTTCCTTCTTCTACT
GGCTTCTTCGGGAGTTGAAGCCGGGGCGGGTACCGGATGGACTGTATATCCCCCACTA
GCCGGGAATCTTGCCCATGCCGGCGCCTCTGTTGACTTAACCATCTTCTCCCTTCATCT
GGCTGGTATTTCCTCCATCCTCGGGGCCATTAACTTCATCACAACTATTATTAACATGAA
ACCTCCTGCAATCTCACAATACCAAACCCCACTTTTCGTGTGAGCCGTCCTAATTACAG
CCGTACTCCTTCTCCTCTCACTTCCGGTTCTCGCTGCCGGTATTACAATGCTTCTCACAG
ACCGGAACCTAAATACAACCTTCTTTGATCCGGCAGGTGGAGGAGACCCCAT
>Rhinogobius_sp 5_SZ284
GACATTGGCACCCTTTATCTTGTATTTGGTGCCTGAGCTGGAATAGTGGGCACGGCCTT
GAGCCTCCTTATTCGAGCCGAGCTAAGCCAGCCCGGAGCCCTTCTGGGCGATGACCAA
ATTTATAATGTTATCGTTACAGCTCATGCTTTCGTAATAATTTTCTTTATAGTAATACCAAT

CATGATTGGGGGTTTCGGAAACTGGCTTATTCCCCTAATAATTGGTGCGCCAGATATAGC
CTTCCCTCGGATAAATAACATAAGCTTTTGACTTCTGCCCCCATCCTTCCTTCTTCTACT
GGCTTCTTCGGGAGTTGAAGCCGGGGCGGGTACCGGATGGACTGTATATCCCCCACTA
GCCGGGAATCTTGCCCATGCCGGCGCCTCTGTTGACTTAACCATCTTCTCCCTTCATCT
GGCTGGTATTTCCTCCATCCTCGGGGCCATTAACTTCATCACAACTATTATTAACATGAA
ACCTCCTGCAATCTCACAATACCAAACCCCACTTTTCGTGTGAGCCGTCCTAATTACAG
CCGTACTCCTTCTCCTCTCACTTCCGGTTCTCGCTGCCGGTATTACAATGCTTCTCACAG
ACCGGAACCTAAATACAACCTTCTTTGATCCGGCAGGTGGAGGAGACCCCAT
>Rhinogobius_sp 6_SZ177
GACATTGGCACCCTTTATCTTGTATTTGGTGCCTGAGCTGGGATAGTGGGCACGGCCTT
GAGCCTCCTTATTCGAGCCGAGCTTAGCCAGCCCGGAGCCCTTCTGGGCAATGACCAA
ATCTATAATGTTATCGTTACAGCTCATGCTTTCGTAATAATCTTCTTTATAGTAATACCAAT
TATGATTGGGGGTTTCGGAAACTGGCTTGTTCCCCTAATGATTGGTGCACCAGACATAG
CCTTCCCTCGAATAAATAATATAAGCTTTTGACTTCTCCCCCCATCCTTCCTTCTTCTATT
GGCTTCTTCGGGAGTTGAAGCCGGGGCAGGTACCGGATGAACTGTATATCCCCCACTA
GCCGGGAATCTTGCCCATGCCGGCGCCTCTGTTGACTTAACCATCTTCTCCCTCCATCT
GGCTGGTATTTCCTCCATCCTCGGGGCCATTAACTTCATCACAACTATTATTAACATGAA
ACCTCCTGCAATCTCACAATACCAAACTCCACTTTTCGTGTGAGCCGTCTTAATTACAG
CCGTACTTCTACTCCTCTCACTTCCGGTTCTCGCTGCCGGTATTACAATGCTTCTCACAG
ACCGAAACCTAAATACGACCTTCTTTGATCCGGCAGGTGGAGGAGACCCCAT
>Xenocypris_sp_SZ441
GACATTGGCACCCTTTATCTTGTATTTGGTGCCTGAGCCGGAATAGTGGGAACCGCTCT
AAGCCTTCTCATTCGAGCCGAACTAAGTCAACCCGGATCACTTCTGGGCGATGACCAA
ATTTATAATGTTATTGTTACTGCCCATGCCTTCGTAATAATTTTCTTTATAGTAATACCAAT
TCTTATTGGAGGGTTTGGAAATTGACTCGTTCCACTAATAATTGGAGCGCCTGATATGG
CATTCCCACGAATAAACAACATAAGCTTCTGACTTCTACCTCCTTCTTTCCTCCTGCTAT
TAGCCTCTTCCGGAGTCGAGGCCGGAGCTGGGACAGGATGAACAGTTTACCCGCCACT
CGCAGGCAACCTTGCCCATGCTGGAGCATCCGTAGACCTAACAATTTTCTCACTTCACC
TAGCAGGTGTATCATCAATTCTAGGGGCAATTAACTTCATCACTACAACTATTAACATGA
AACCACCAGCCATTTCCCAATACCAAACACCTCTGTTCGTCTGAGCTGTACTTGTAACA
GCCGTACTTCTTCTCCTATCACTACCAGTCCTAGCTGCCGGAATTACAATGCTCCTTACA
GACCGAAATCTTAACACCACATTCTTCGACCCGGCAGGAGGAGGAGACCCAAT
