# Supplementary material for: Unraveling the Drifting Larval Fish Community in a Large Spawning Ground in the Middle Pearl River Using DNA Barcoding
Source: Animals (Basel). 2022 Sep 24;12(19):2555. doi: 10.3390/ani12192555 (PMC9559676; doi:10.3390/ani12192555)
Supplement: Supplementary file 1 [file animals-12-02555-s001.zip › Table S5.pdf]

	Total species	Cyprinidae species	Ratio
Dongta Spawn ground	28	21	75.0%
Larval pool in Lower Hongshui River [1]	14	10	71.4%
Larval pool in Lower Pearl River [2]	37	27	73.0%
Field survey in 2005 [3]	99	46	46.5%
Field survey in 2018 [4]	96	48	50.0%

References

1. Chen, W.; Zhu, S.; Yang, J.; Li, X.; Li, Y., Li, J. DNA barcoding reveals the temporal community composition of drifting fish eggs in the lower Hongshui River, China. *Ecol. Evol.* **2021**, 11(16), 11507-11514.
2. Chen, W.; Li, C.; Yang, J.; Zhu, S.; Li, J.; Li, Y., Li, X. Temporal species-level composition of larvae resources in the lower Pearl River drainage and implications for species' reproductive cycles. *Gene* **2021**, 776, 145351.
3. Li, J.; Li, X.; Jia, X.; Li, Y.; He, M.; Tan, X.; Wang, C., Jiang, W. Evolvement and diversity of fish community in Xijiang River. *J. Fish. Sci. China* **2010**, 17(2), 298-311, (In Chinese with English Abstract)
4. Zhang, Y.; Huang, D.; Li, X.; Liu, Q.; Li, J.; Li, Y.F.; Yang, J.P., Zhu, S.L. Fish community structure and environmental effects of West River. *South China Fish. Sci.* **2020**, 16(1), 42-52, (In Chinese with English Abstract)
